# Three-dimensional controlled growth of monodisperse sub-50 nm heterogeneous nanocrystals

**DOI:** 10.1038/ncomms10254

**Published:** 2016-01-08

**Authors:** Deming Liu, Xiaoxue Xu, Yi Du, Xian Qin, Yuhai Zhang, Chenshuo Ma, Shihui Wen, Wei Ren, Ewa M. Goldys, James A. Piper, Shixue Dou, Xiaogang Liu, Dayong Jin

**Affiliations:** 1 Laboratory of Advanced Cytometry, ARC Centre of Excellence for Nanoscale BioPhotonics, Department of Physics and Astronomy, Macquarie University, Sydney, New South Wales 2109, Australia; 2 Faculty of Science, Institute for Biomedical Materials and Devices, University of Technology Sydney, New South Wales 2007, Australia; 3 Institute for Superconducting and Electronic Materials, Innovation Campus, University of Wollongong, New South Wales 2522, Australia; 4 Institute of Materials Research and Engineering, 3 Research Link, Singapore 117602, Singapore; 5 Department of Chemistry, National University of Singapore, 3 Science Drive 3, Singapore 117543, Singapore

## Abstract

The ultimate frontier in nanomaterials engineering is to realize their composition control with atomic scale precision to enable fabrication of nanoparticles with desirable size, shape and surface properties. Such control becomes even more useful when growing hybrid nanocrystals designed to integrate multiple functionalities. Here we report achieving such degree of control in a family of rare-earth-doped nanomaterials. We experimentally verify the co-existence and different roles of oleate anions (OA^−^) and molecules (OAH) in the crystal formation. We identify that the control over the ratio of OA^−^ to OAH can be used to directionally inhibit, promote or etch the crystallographic facets of the nanoparticles. This control enables selective grafting of shells with complex morphologies grown over nanocrystal cores, thus allowing the fabrication of a diverse library of monodisperse sub-50 nm nanoparticles. With such programmable additive and subtractive engineering a variety of three-dimensional shapes can be implemented using a bottom–up scalable approach.

## Abstract (web summary)

Atomic-level control over size, shape and surface composition of nanoparticles is vital for developing materials with integrated multiple functionalities. Here, the authors probe the different roles of oleate ions and oleic acid molecules and their effects on growth mechanisms for sub-50 nm nanoparticles.

## Graphical abstract

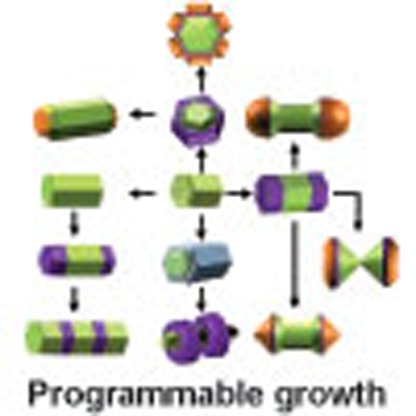

Nanocrystal engineering, design and fabrication of nanocrystals with desirable size, shape^1^,^2^,^3^,^4^,^5^,^6^, surface properties^7^ and composition^8^,^9^ is attracting growing interest due to its essential role in fundamental research and commercial relevance. Rare-earth-doped upconversion nanocrystals have recently emerged as the new generation of functional nanomaterials, because they exhibit exceptional optical, magnetic and chemical properties underpinning their diverse applications. In particular, alkaline rare-earth fluoride (AREF_4_) nanocrystals^10^,^11^,^12^, including hexagonal-phase β-NaYF_4_, β-NaGdF_4_, β-NaNdF_4_ or β-NaLuF_4_ are used in full-colour displays^12^,^13^, photovoltaics^14^, security inks^15^, forensic science^16^, autofluorescence-free biomolecular sensing^17^,^18^,^19^, multimodal *in vivo* bio-imaging (fluorescence, magnetic resonance imaging, X-ray, SPECT and so on.)^20^ and theranostics^17^,^21^,^22^,^23^. A trial-and-error approach is frequently used to produce nanoparticles with spherical, rod-like or other shapes^24^,^25^,^26^ by varying dopant concentrations and/or constituent materials^27^, reaction time and temperature^28^,^29^,^30^,^31^. This random sampling of vast, multidimensional parameter space, needs to be done rationally, with proper understanding of the underpinning growth mechanisms.

Here we find that oleate anions (OA^−^), the dissociated form of oleic acid molecules (OAH), have variable, dynamic roles in mediating the growth of AREF_4_ nanocrystals. This allows us to introduce a molecular approach to tailoring the shape and composition of AREF_4_ nanocrystals. This new method is based on a selective epitaxial core–shell growth process in the presence of oleic acid, commonly used as a surfactant during the synthesis of β-AREF_4_ nanocrystals^32^. Drawing inspiration from the recently discovered co-existence of oleic acid molecules (OAH) and their dissociated form, oleic acid ions (OA^−^) in the binary systems of PbS^33^ and PbSe nanocrystals^34^, we hypothesize that the change in the ratio of OA^−^ to OAH could influence the interaction of these ligands with the particle surface and hence the resulting morphology. Our computational modelling (Fig. 1, Supplementary Figs 1–6, Supplementary Notes 1 and 2 and Supplementary Table 1) and experimental results (Figs 2, 3, 4, Supplementary Figs 7–35, Supplementary Tables 2 and 3 and Supplementary Notes 3–18) demonstrate that the preferential affinity of OAH and OA^−^ to different crystalline facets dictates the formation of nanocrystals of different shape. Importantly, we demonstrate that the precise control over the shell thickness and the particle shape can be achieved by deliberately switching the passivation, additive and subtractive roles of these surfactants.

## Results

### Computational modelling

**Figure 1:**
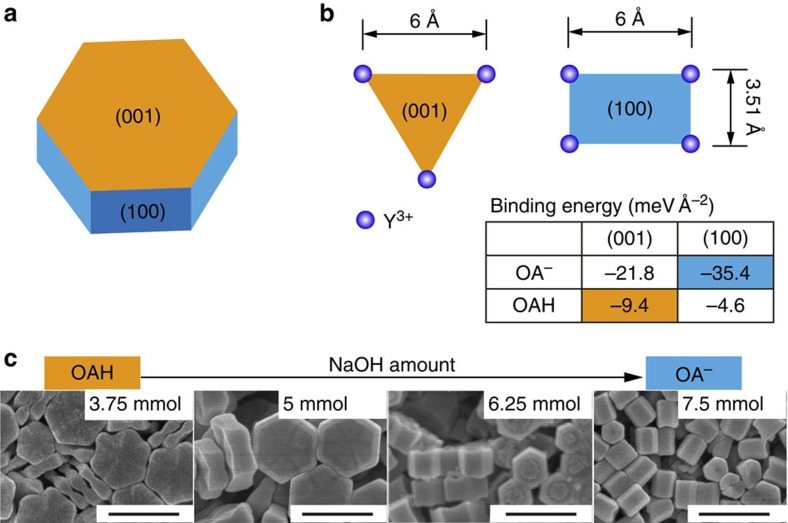
Preferred molecular bonding models of OA^−^ and OAH. (**a**) The schematic shape of a *β*-NaYF_4_ nanocrystal chosen as the core for directional epitaxial growth in this work. The hexagonal cylinder consists of the (001) facets at the ends and identical (100) and (010) facets around the cylinder sides. (**b**) The Y^3+^ arrangements and binding energies (see insert table) of OAH and OA^−^ on the most stable (001) and (100) facets. The Y^3+^ atoms form equilateral triangles with a length of 6 Å in the relaxed (001) surface, while rectangles are observed in the (100) surface with a shorter length of 3.51 or 3.69 Å; (**c**) SEM characterization of submicron-sized nanocrystals synthesized using the hydrothermal route (detailed synthesis is included in the method; scale bar, 500 nm).

To quantify the surface coordination chemistry between β-NaYF_4_ surface and OAH and OA^−^ ligands, we performed first-principles calculations based on density functional theory using CASTEP (CAmbridge Serial Total Energy Package)^35^. As shown in Fig. 1b and Supplementary Fig. 1, we treated the (001) and (100) planes of the *β*-NaYF_4_ nanocrystals terminated with specific atomic arrangement as the most stable facets according to the calculated surface energies. Considering that the oxygen moiety in the ligands has a strong binding affinity to Y^3+^ ions at the particle surface^36^, we modelled the interactions between the OAH and OA^−^ molecules and the Y^3+^ ions under a number of conditions, such as different adsorption configurations (Supplementary Figs 2 and 3 and Supplementary Note 1), ligand chain length and ligand coverage (Supplementary Figs 4 and 5 and Supplementary Note 2). The key conclusion from these simulations is that OA^−^ preferentially binds to RE^3+^ ions exposed on the (100) facet of the hexagonal fluoride nanocrystal, with a much higher binding energy (−35.4 eV) than on the (001) facet (−21.8 eV). It should be noted that the OAH molecule binds with a higher probability to the (001) facet than the (100) facet and has relatively small binding energies of −9.4 eV and −4.6 eV, respectively, on each of these facets (Supplementary Table 1). Our charge analysis (Supplementary Fig. 6) further indicates that such selective binding is attributed to the difference in the atomic arrangements of these two facets (Fig. 1b), giving rise to different charge transfer paths between the ligands and the surface ions.

### Controlled epitaxial growth direction

**Figure 2:**
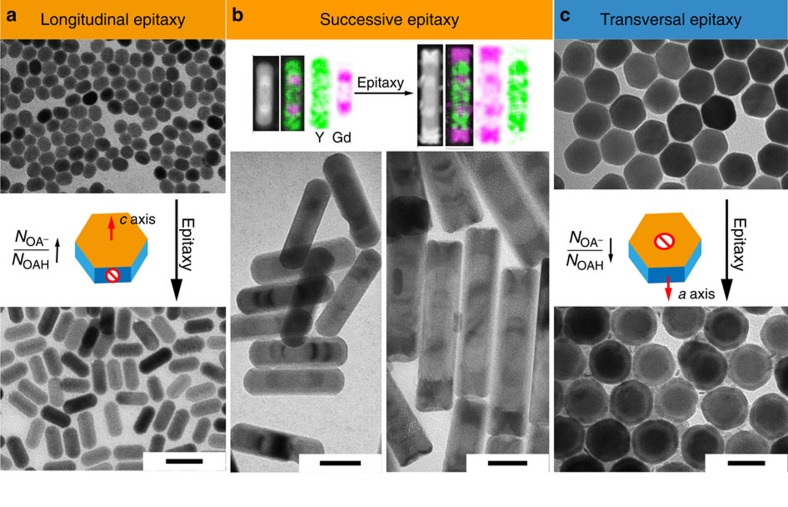
Physical characterization of epitaxial growth of NaReF_4_ NCs. (**a**) NaYF_4_ core and homogenous NaYF_4_ NCs after epitaxial growth of NaYF_4_ in longitudinal direction with 0.5 mmol NaOH and 9.5 mmol OA at 310 °C for 1 h; (**b**) five-section and seven-section ‘bamboo-shaped' NaYF_4_/NaGdF_4_ NRs formed by successive heterogeneous growth of periodical shells of NaGdF_4_–NaYF_4_ and NaGdF_4_–NaYF_4_–NaGdF_4_ onto NaYF_4_ core in the longitudinal direction, with 0.5 mmol NaOH and 0.4 mmol KOH and 9.5 mmol OA at 310 °C. Upper part of the panel shows elemental mapping of Y and Gd; (**c**) NaYF_4_ core and heterogeneous NaYF_4_/NaGdF_4_ NCs after epitaxial growth of NaGdF_4_ in the transversal direction with 0.15 mmol NaOH and 19 mmol OA at 290 °C for 3 h; the dimensions of individual nanocrystal were analysed statistically and included in the Supplementary Figs 10, 20–24. Scale bar, 50 nm.

The binding preferences of OAH and OA^−^ molecules to different facets were first used to induce longitudinal epitaxial growth. We demonstrated (Fig. 1c) that sub-micrometre-sized NaYF_4_ crystals of different aspect ratios could be prepared by tuning the concentration ratio of OA^−^ to OAH in the hydrothermal synthesis system. As shown in Supplementary Fig. 7, higher concentrations of OA^−^ encourage epitaxial growth along a longitudinal direction. A similar effect was observed in the synthesis of sub-50 nm NaYF_4_ nanoparticles prepared by a co-precipitation method. Figure 2a,b show that high concentration of NaOH leads to longitudinally grown nanoparticles because of a large concentration of passivating OA^−^ ions on the (100) facets (Supplementary Figs 8–10). The zeta potential of +20 mV for NaYF_4_ nanocrystals after the removal of ligands (Supplementary Fig. 11) shows that the RE^3+^ cations are more abundant on the crystal surfaces than the F^−^ ions. We further systemically studied other possible factors that could influence the epitaxial shell growth (experimental details in Supplementary Methods), including the reaction temperature (Supplementary Fig. 12 and Supplementary Note 3), the oleic acid concentration (Supplementary Fig. 13 and Supplementary Note 4), the F^−^ ion concentration (Supplementary Fig. 14 and Supplementary Note 5) and the Na^+^ concentration (Supplementary Fig. 15 and Supplementary Note 6). From these results, we confirm that the ratio of OA^−^/OAH is a key factor that determines the epitaxial shell growth direction. However other parameters also have an effect on the growth speed or can change the OA^−^/OAH ratio that indirectly affects the direction of growth. To rule out the effect of OH^−^ on longitudinal growth, we added sodium oleate as the sodium source instead of hydroxide and identical results were obtained (Supplementary Fig. 16 and Supplementary Note 7). Supplementary Figures 17 and 18 further confirm that high ratio of OA^−^/OAH directs longitudinal deposition of heterogeneous shells (NaGdF_4_) on the end surfaces of NaYF_4_ core. Interestingly, subtractive growth (dissolution) is observed from their side (100) surfaces. This results in concurrent decrease of the core width from 26 to 18 nm, thus producing dumbbell-shaped nanocrystals (Supplementary Note 8).

Moreover, we found that the addition of KOH further accelerates longitudinal growth rate (Supplementary Fig. 19 and Supplementary Note 9) due to a higher dissociation constant of KOH than NaOH, which increases the dissociation of OAH producing more OA^−^. With the aid of KOH, heterogeneous ‘bamboo-shaped' nanorods (NRs) with sharp edges were formed in a stepwise manner with a length of up to 173 nm (Fig. 2b, Supplementary Fig. 21 and Supplementary Note 10). The interesting one-dimension architecture of ‘bamboo-shaped' NRs suggests that integrated multiple functionalities can be built. Thus our new platform enables rational design and facile synthesis of multiple sections of rare-earth-doped heterogeneous materials and investigation of their interactions and functions within a single integrated rod. We were also able to induce transversal epitaxial growth by increasing the amount of OAH and reducing the amount of NaOH. At a reaction temperature of 290 °C, the transversal growth was observed and NaGdF_4_ rings of 7-nm-thick around the NaYF_4_ cores formed without a measurable change in the longitudinal direction (Fig. 2c, Supplementary Figs 23 and 24 and Supplementary Note 11). Notably, the dissolution of the (100) facets of the cores took place as well, and the width of the core was, again, reduced from 49 to 30 nm at both ends. The observed dissolution always occurred on the (100) facets in both cases of longitudinal and transversal growth. This is consistent with the strong chelating character of OA^−^ on the (100) facet, and with the fact that NaYF_4_ is dissolved faster than NaGdF_4_ because NaYF_4_ is comparably less energetically stable^12^. To shed more light on this issue, we provided more evidence in the Supplementary Fig. 25 and Supplementary Note 12 to show that the dissolution of core is caused by the thermal stability difference between core and shell materials in presence of OA^−^ which leads to higher binding strength on the side surfaces. By comparing growth of NaTbF_4_ as shell or NaYbF_4_ as shell on a NaYF_4_ core (Supplementary Fig. 25), we demonstrate that the dissolution of the core requires the shell materials to have higher thermal stability than the core material. Larger difference of thermal stability between core and shell result in a higher dissolution rate.

### Controlled migration growth

**Figure 3:**
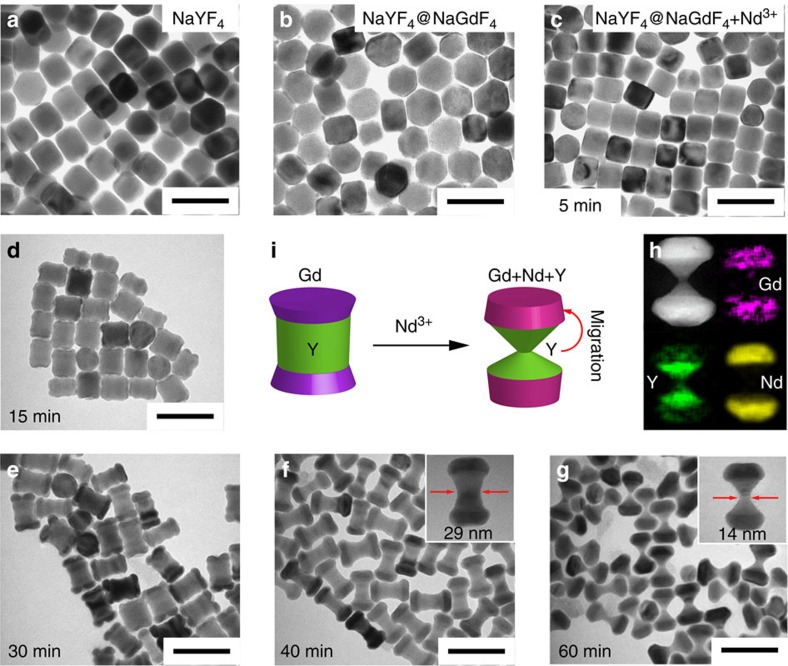
Evolution of morphology and composition in migration growth. (**a**,**b**) NaGdF_4_ growth along the longitudinal direction onto the ends of the NaYF_4_ core; (**c**) transmission electron microscope image of the sample stopped 5 min after reacting with NaGdF_4_/NaYF_4_ nanocrystals in the presence of Na^+^, K^+^, Nd^3+^, OA^−^ and in the absence of F^−^ at 310 °C, dissolution occurs first; (**d**–**g**) real-time monitoring of the epitaxial growth of NaNdF_4_ along the longitudinal direction onto NaYF_4_–NaGdF_4_ nanocrystals, involving the dissolution of NaYF_4_ and NaGdF_4_ from the transversal surfaces of the crystal and their subsequent re-growth onto the NaNdF_4_ nanocrystals in the presence of Na^+^, K^+^, Nd^3+^, OA^−^ and absence of F^−^ ions at 310 °C. (**h**) HAADF-STEM image with elemental mapping results of the samples stopped after 60 min of reaction to confirm the distributions of Y^3+^, Gd^3+^, Nd^3+^ ions within a single NaYF_4_/NaGdF_4_/NaNdF_4_ nanocrystal. (**i**) schematic processes of dissolution of NaYF_4_/NaGdF_4_ and the sequent epitaxial growth of NaNdF_4_ in the longitudinal direction and the migration growth of F^−^, Y^3+^ and Gd^3+^ ions (scale bar, 100 nm).

By combining the approaches of longitudinal and transversal growth and selective dissolution with consideration of lattice mismatch (Supplementary Tables 2 and 3), we synthesized a variety of three-dimensional (3D) hybrid nanostructures (Supplementary Figs 26–34). Figure 3 shows a typical example of real-time evolution of morphology and composition of the NaYF_4_/NaGdF_4_/NaNdF_4_ NCs, including the dissolution process of the NaYF_4_/NaGdF_4_ nanocrystals and subsequent longitudinal growth of NaNdF_4_. The dissolution of NaYF_4_/NaGdF_4_ is initiated by the OA^−^ adsorbed on the surface of the nanocrystals. The concomitant depletion of dissolved F^−^ ions used for longitudinal growth of NaNdF_4_ in the presence of high concentration of OA^−^ facilitates the dissolution of NaYF_4_/NaGdF_4_ nanocrystals and this, in turn, promotes longitudinal growth of NaNdF_4_. Following the dissolution of the Y^3+^ and Gd^3+^ ions from the surface of NaYF_4_–NaGdF_4_ nanocrystals, these ions then participate in the epitaxial growth of NaNdF_4_ nanocrystals, as evidenced by the elemental mapping (Fig. 3h). Moreover, our real-time sampling transmission electron microscope data further confirmed the underpinning mechanism (Fig. 3a–g, Supplementary Figs 26–28). The size of nanocrystal core decreased significantly in the first 5 min, indicating that the dissolution rate of the nanocrystals is faster than their growth rate. After 15 min, new material started to form at the top and at the bottom ends of the core with simultaneous decrease of the nanocrystal core width. This observation rules out ‘surface mobility' (‘atom diffusion') as the possible driving force behind the formation of the final shell, otherwise it is expected that the dissolution of NaYF_4_ and growth of NaNdF_4_ would occur at the same time. The only mechanism which explains the shape of this nanocrystal is that the absence of F^−^ source in the reaction solution at its beginning prevents growth of NaNdY_4_ until the concentration of released F^−^ source exceeds a certain threshold.

Our control experiments (Supplementary Fig. 29 and Supplementary Note 15) further support the mechanism of OA^−^-induced dissolution in which a firm bonding of the surfactant OA^−^ to the surface RE^3+^ cations is the main factor responsible for the removal of the surface crystalline layers (experimental details in Supplementary Methods). As shown in Supplementary Fig. 29, we applied transversal growth approach to first grow a layer of NaGdF_4_ on the side surfaces of NaYF_4_ core. We see that smaller mismatch of NaGdF_4_ versus NaNdF_4_ compared with the NaYF_4_ versus NaNdF_4_ fails to direct the transversal migration growth of the NaNdF_4_ on the side surfaces of NaGdF_4_. Instead, dissolution occurs in the first 10 min of the reaction (Supplementary Fig. 29a,b) and both dissolution from the side surfaces and epitaxial growth of NaNdF_4_ on the end surfaces of NaGdF_4_/NaYF_4_ cores result in a thinner and longer nanocrystal.

Guided by the principle that the ratio of OA^−^/OAH controls the direction of epitaxial shell growth, we further demonstrated (as shown in Supplementary Fig. 30 and Supplementary Note 16) that a low ratio of OA^−^/OAH at a lower temperature directs the migration growth along transverse direction. This enables the formation of heterogeneous NaYF_4_/NaGdF_4_/NaNdF_4_ nanocrystals in the shape of a flower, although in this case the dissolution process on the side surfaces of nanocrystals is much less efficient because there are too few OA^−^ ligands bound to RE^3+^ cations on the (100) facet. Two additional experiments (Supplementary Note 17) demonstrate that well established parameters, such as reagent concentration, can be further applied to fine-tune our programmable protocols for other types of heterogeneous nanocrystals. During the formation of hourglass-shaped nanocrystals, the decrease in the amount of Nd^3+^ source is found to hinder the migration growth process and yield sharper tips (Supplementary Fig. 31), whereas a supply of additional F^−^ ions in the reaction increases the diameter of dumbbell ends with round tips (Supplementary Fig. 32). Such level of fine tuning to grow progressively sharper tips may suggest future rational methods, for example to optimize tip-sensitive physical and biochemical properties of NRs.

**Figure 4:**
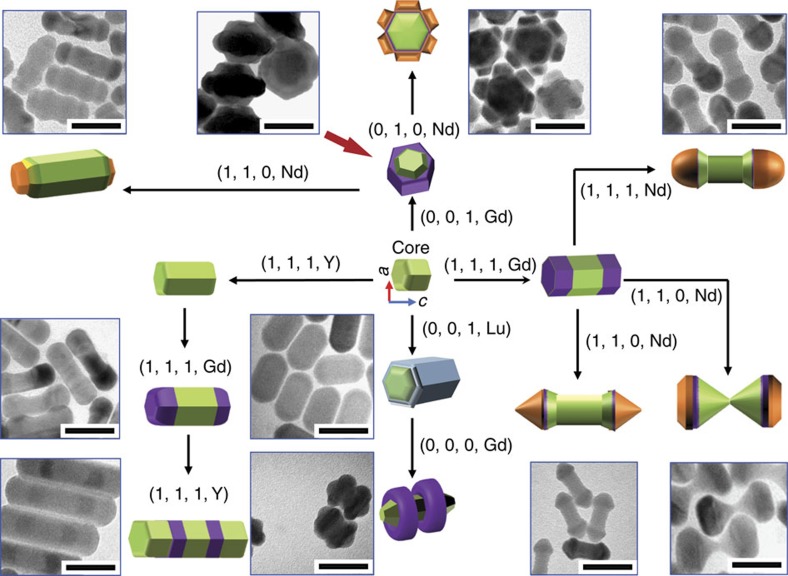
Programmable routes for fabricating 3D nano-architectures. The four digital condition codes (R, T, F and RE) represent different reaction conditions where: *R*=0, represents a low ratio of OA^−^/OAH; *R*=1, represents a high ratio of OA^−^/OAH; *T*=0, where the temperature is at 290 ^o^C; *T*=1, where the temperature is at 310 °C; *F*=0, which designates the absence of an *F*^−^ ion source; *F*=1, indicates the presence of an *F*^−^ ion source; RE=Y, with a rare earth Y^3+^ ion source; RE=Gd, with Gd^3+^ ion source; RE=Lu, with a Lu^3+^ source; RE=Nd, with Nd^3+^ source. By combining these different growth processes into a synthesis procedure, a variety of complex NaREF_4_ nanostructures are fabricated as shown in the transmission electron microscope images, including hourglass shaped NaYF_4_/NaGdF_4_/NaNdF_4_ nanocrystals, NaYF_4_/NaGdF_4_/NaNdF_4_ nano-flowers, NaYF_4_/NaLuF_4_ co-axial nano-cylinders, NaYF_4_/NaLuF_4_/NaGdF_4_ nanoscale spins with double rings, and NaYF_4_/NaGdF_4_/NaNdF_4_ nano-dumbbells with smooth or sharp ends (scale bar, 50 nm).

Figure 4 shows an array of heterogeneous NaREF_4_ nanostructures synthesized by carrying out specific sequences of longitudinal, transversal growth, selective dissolution and directional migration growth of epitaxial shells in the presence of various OA^−^/OAH ratios. To the best of our knowledge, these sub-50 nm nanoparticles are the smallest 3D objects prepared by a bottom–up additive and subtractive process. To illustrate the application of this novel method we designed and synthesized multifunctional NaYF_4_/NaLuF_4_/NaGdF_4_ heterogeneous nanocrystals with two NaGdF_4_ rings on a NaLuF_4_/NaYF_4_ NRs (Supplementary Figs 33 and 34 and Supplementary Note 18). The hexagonal-phase NaYF_4_ nanocrystal is an efficient luminescence upconversion material^37^. The addition of NaLuF_4_ enables X-ray computed tomography^38^, whereas using NaGdF_4_ enables magnetic resonance imaging^39^. To the best of our knowledge, this work presents the first controlled fabrication of sub-50 nm 3D shaped heterogeneous nanocrystals logically programmed by the combinational approaches of OA^−^-assisted longitudinal growth, transversal growth and selective crystalline facet dissolution with consideration of crystallographic mismatch rates.

## Discussion

The nanoscale engineering capability presented in this work enables quantitative studies which are virtually impossible by conventional approaches. We anticipate that optical properties of these nanostructures can be designed to precisely promote or inhibit inter-particle energy transfer. Similarly, magnetic properties may be optimized to enhance magnetic resonance imaging by correlating the morphology with the surface distribution of magnetic signals. In addition, such hybrid nanomaterials may be used as a platform for transporting biologically important molecules across cell membranes. Furthermore, access to a new library of precisely controlled shapes of nanoparticles provide a novel approach for the targeted delivery in nanomedicine where optimized morphologies of these nanoscale molecular carriers will yield greater efficiencies. This process could be further facilitated by harnessing the anisotropic properties of different types of nanoparticles that permit diverse surface functionalizations and multi-modal bio-conjugations. The concept presented in this work may further advance our current capabilities of nanoscale programmable and reproducible engineering of new classes of heterogeneous materials in scalable quantities. Our findings may lead to a new class of multifunctional nanomaterials and provide the groundwork for developing previously unforeseen applications of nanoparticles with complex programmable shapes and surface properties.

## Methods

### Hydrothermal synthesis of NaYF_4_ crystal

The β-NaYF_4_ disks were synthesized via a slightly modified hydrothermal reaction. In a typical experiment, NaOH (3.75 mmol) was first dissolved into 1.5 mL of double distilled water, followed by the addition of OA (7.5 mmol) and ethanol (2.5 mL) while undergoing vigorous stirring. Thereafter, an aqueous solution of NaF (0.5 M; 2 mL) was added to form a turbid mixture. Subsequently, a 1.2 mL aqueous solution of YCl_3_ (Yb^3+^/Tm^3+^=10/0.5 mol%; 0.2 M) was added and the solution was stirred for 20 min. The resulting mixture was then transferred into a 14 mL Teflon-lined autoclave and heated to 220 °C and the temperature maintained for 12 h. After cooling down to room temperature, the reaction product was isolated by centrifugation and washed with ethanol. In this work, different amounts of NaOH were added to adjust the ratio of OA^−^/OAH by its reaction with OAH to form OA^−^.

### NaYF_4_ nanocrystal cores

In a typical procedure, 4 ml of methanol solution of YCl_3_ (2.0 mmol) was magnetically mixed with OA (38 mmol) and ODE (93 mmol) in a 100-ml three-neck round-bottom flask. The mixture was then degassed under the Ar flow and then heated to 150 °C for 30 min to form a clear solution, before cooling to room temperature. 15 ml of methanol solution containing NH_4_F (8 mmol) and NaOH (5 mmol) was added to the solution of YCl_3_ in OA and ODE and stirred for 60 min. The mixture solution was slowly heated to 110 °C and kept at 110 °C for 30 min to completely remove methanol and any residual water. The mixture solution was then quickly heated to the reaction temperature of 300 °C and aged for 1 h. After the solution was left to cool down to room temperature, ethanol was added to precipitate the nanocrystals. The product was washed with cyclohexane, ethanol and methanol for at least 4 times, before the final NaYF_4_ nanocrystals were re-dispersed in 10 ml cyclohexane in preparation for their further use.

### Longitudinal growth of NaYF_4_ NRs

YCl_3_ (0.2 mmol) in 1 ml methanol solution was magnetically mixed with OA (9.5 mmol) and ODE (25 mmol) in a 50-ml three-neck round-bottom flask. The mixture was degassed under Ar flow and heated to 150 °C for 30 min to form a clear solution, and then cooled to room temperature. Methanol solution (5 ml) containing NH_4_F (0.8 mmol) and NaOH (0.5 mmol) was added and stirred for 60 min. The solution was slowly heated to 110 °C and kept at 110 °C for 30 min to completely remove methanol and residual water. The solution was then injected with 0.2 mmol NaYF_4_ of nanocrystals in cyclohexane and the mixture kept at 110 °C for another 10 min to evaporate the cyclohexane. Then, the reaction mixture was quickly heated to 310 °C and aged for 1 h.

### NaGdF_4_/NaYF_4_ nano-dumbbells

GdCl_3_ (0.2 mmol) in 1 ml methanol solution was magnetically mixed with OA (9.5 mmol) and ODE (25 mmol) in a 50-ml three-neck round-bottom flask. The mixture was degassed under an Ar flow and heated to 150 °C for 30 min to form a clear solution, and then cooled to room temperature. Methanol solution (4 ml) containing NH_4_F (0.8 mmol) and NaOH (0.5 mmol) was added to the OA and ODE solution and stirred for 60 min. The solution is slowly heated to 110 °C and kept at 110 °C for 30 min to remove methanol and the remaining water completely. Then, 0.2 mmol of NaYF_4_ core nanocrystals in cyclohexane was injected into the reaction solution. After holding the reaction temperature at 110 °C for further 10 min to evaporate all cyclohexane, the reaction mixture was quickly heated to 310 °C and aged for 1 h.

### NaGdF_4_/NaYF_4_ NRs by adding KOH

GdCl_3_ (0.2 mmol) in 1 ml of methanol solution was magnetically mixed with OA (9.5 mmol) and ODE (25 mmol) in a 50-ml three-neck round-bottom flask. The mixture was degassed under Ar flow and heated to 150 °C for 30 min to form a clear solution, before cooling to room temperature. Methanol solution (5 ml) containing NH_4_F (0.8 mmol), KOH (0.4 mmol) and NaOH (0.5 mmol) was added into the OA and ODE solution and stirred for 60 min. The solution was slowly heated to 110 °C and kept at 110 °C for 30 min to remove the methanol and water completely. The reaction mix was then injected with 0.2 mmol of NaYF_4_ core nanocrystals in cyclohexane, into the reaction solution. After holding the reaction mix at 110 °C for further 10 min to evaporate all cyclohexane, the mixture was heated rapidly to 310 °C before aging for 1 h at this temperature.

### NaYF_4_/NaGdF_4_/NaYF_4_ NCs in a bamboo-like shape

0.2 mmol of YCl_3_ in 1 ml of methanol solution was magnetically mixed with OA (9.5 mmol) and ODE (25 mmol) in a 50-ml three-neck round-bottom flask. The mixture was degassed under Ar flow and heated to 150 °C for 30 min to form a clear solution, and then cooled to room temperature. Methanol solution (5 ml) containing NH_4_F (0.8 mmol), KOH (0.4 mmol) and NaOH (0.5 mmol) was added into the OA and ODE solution and stirred for 60 minutes. The solution was slowly heated to 110 °C and kept at 110 °C for 30 min to remove the methanol and water completely. The reaction solution was then injected with 0.2 mmol of NaYF_4_/NaGdF_4_ NRs in cyclohexane solution. After the reaction at 110 °C for a further 10 min to evaporate all the cyclohexane, the reaction mixture was quickly heated to 310 °C and held at this temperature for 1 h.

### NaYF_4_/NaGdF_4_/NaYF_4_/NaGdF_4_ NCs in a bamboo-like shape

The same procedure for synthesizing NaYF_4_/NaGdF_4_/NaYF_4_ NCs in bamboo-like shape was repeated, and then followed by the injection of 0.2 mmol of the five-section NaYF_4_/NaGdF_4_/NaYF_4_ nano-bamboos which acted as the core, all in cyclohexane solution, into the reaction solution. After holding at 110 °C for a further 10 min to evaporate all cyclohexane, the reaction mixture was quickly heated to 310 °C and held again for 1 h.

### NaYF_4_/NaGdF_4_/NaNdF_4_ NCs in an hourglass shape

NdCl_3_ (0.4 mmol) in 2 ml of methanol solution was magnetically mixed with OA (9.5 mmol) and ODE (25 mmol) in a 50-ml three-neck round-bottom flask. The mixture was degassed under Ar flow and heated to 150 °C for 30 min to form a clear solution, and then cooled to room temperature. Methanol solution (5 ml) containing KOH (0.8 mmol) and NaOH (0.8 mmol) was added and stirred for 60 min. The solution was slowly heated to 110 °C and kept at 110 °C for 30 min to completely remove the methanol and some of the water. It was then injected with 0.1 mmol 50 nm × 60 nm NaYF_4_/NaGdF_4_ nano-prisms particles, in a solution of cyclohexane. After having been kept at 110 °C for another 10 min to evaporate all cyclohexane, the reaction mixture was quickly heated to 310 °C. Samples (500 ul) of the reaction solution were collected each time with a syringe at 5, 15, 30, 40, 50 and 60 min after the start of the reaction.

### Transversal growth of NaGdF_4_ shell onto NaYF_4_ core

GdCl_3_ (0.1 mmol) in 1 ml methanol solution was magnetically mixed with OA (19.0 mmol) and ODE (18.7 mmol) in a 50-ml three-neck round-bottom flask. The mixture was degassed under Ar flow and heated to 150 °C for 30 min to form a clear solution, and then cooled to room temperature. Methanol solution (3 ml) containing NH_4_F (0.4 mmol) and NaOH (0.15 mmol) was added into the OA and ODE solution and stirred for 60 min. The solution was slowly heated to 110 °C and kept at 110 °C for 30 min to remove completely the methanol and water. Then 0.1 mmol of the NaYF_4_ cores in cyclohexane solvent were injected into the reaction mix. After being kept at 110 °C for further 10 min to evaporate all cyclohexane, the reaction mixture was quickly heated up to 290 °C and held at that temperature for 3 h.

### Synthesis of NaYF_4_/NaGdF_4_/NaNdF_4_ NCs in flower shape

NdCl_3_ (0.1 mmol) in 1 ml of methanol solution was magnetically mixed with OA (19 mmol) and ODE (18.7 mmol) in a 50-ml three-neck round-bottom flask. The mixture was degassed under Ar flow and heated to 150 °C for 30 min to form a clear solution, and then cooled to room temperature. Methanol solution (5 ml) containing NaOH (0.6 mmol) was added and stirred for 60 min. The solution was slowly heated to 110 °C and kept at 110 °C for 30 min to completely remove the methanol and some of the water. Then, the reaction mix was injected with 0.1 mmol of 50 nm NaYF_4_/NaGdF_4_ nano-prisms prisms (NaGdF_4_ growing on the lateral faces of NaYF_4_ nanocrystal), suspended in a cyclohexane solution. After holding at 110 °C for another 10 min to evaporate all cyclohexane, the reaction mixture was quickly heated to 300 °C. samples (500 ul) of the reaction solution were collected each time with a syringe after 10, 25 and 45 min of the reaction time.

### Synthesis of NaYF_4_/NaGdF_4_/NaNdF_4_ sharp-end dumbbell

NdCl_3_ (0.1 mmol) in 1 ml of methanol solution was magnetically mixed with OA (9.5 mmol) and ODE (25 mmol) in a 50-ml three-neck round-bottom flask. The mixture was degassed under Ar flow and heated to 160 °C for 30 min to form a clear solution, and then cooled to room temperature. Methanol solution (5 ml) containing KOH (0.2 mmol) and NaOH (0.2 mmol) was added and stirred for 60 min. Note: in this reaction no NH_4_F was added to the solution. The solution was slowly heated to 110 °C and kept at 110 °C for 30 min to remove the methanol and the water completely. It was then injected with 0.1 mmol of NaYF_4_/NaGdF_4_ NR particle in suspended in cyclohexane solvent into the reaction solution. After holding at 110 °C for a further 10 min to evaporate all cyclohexane, the reaction mixture was quickly heated to 310 °C and held at this temperature for a further 30 min.

### Synthesis of NaYF_4_/NaGdF_4_/NaNdF_4_ round-end dumbbell

NdCl_3_ (0.1 mmol) in 1 ml of methanol solution was magnetically mixed with OA (9.5 mmol) and ODE (25 mmol) in a 50-ml three-neck round-bottom flask. The mixture was degassed under Ar flow and heated to 160 °C for 30 min to form a clear solution, and then cooled to room temperature. Methanol solution (5 ml) containing NH_4_F (0.3 mmol), KOH (0.2 mmol) and NaOH (0.2 mmol) was added and the mixture was stirred for 60 min. The solution was slowly heated to 110 °C and kept at 110 °C for 30 min to remove the methanol and the water completely. Then, it was injected with 0.1 mmol of NaYF_4_/NaGdF_4_ NRs suspended in cyclohexane into the reaction solution. After being held at 110 °C for further 10 min to evaporate all cyclohexane, the reaction mixture was quickly heated to 310 °C and held for 30 min at this temperature.

### Synthesis of pure *α*-NaGdF_4_ NCs

Methanol solution (2 ml) of GdCl_3_ (1.0 mmol) was magnetically mixed with OA (19 mmol) and ODE (47 mmol) in a 100-ml three-neck round-bottom flask. The mixture was degassed under Ar flow and heated to 150 °C for 30 minutes to form a clear solution, and then cooled to room temperature. Methanol solution (10 ml) containing NH_4_F (4 mmol) and NaOH (2.5 mmol) was added and stirred for 60 min. Then, the solution was slowly heated to 110 °C and kept at 110 °C for 30 min to remove the methanol and water completely. After that, the reaction mixture was quickly heated to 240 °C and aged for 45 min.

### Synthesis of NaLuF_4_/NaYF_4_ NRs

LuCl_3_ (0.1 mmol) in 1 ml method solution was magnetically mixed with OA (19 mmol) and ODE (25 mmol) in a 50-ml three-neck round-bottom flask. The mixture was degassed under Ar flow and heated to 150 °C for 30 min to form a clear solution, and then cooled to room temperature. Methanol solution (2 ml) containing NaOH (0.15 mmol) and 0.4 mmol NH_4_F was added and stirred for 60 min. The solution was slowly heated to 110 °C and kept at 110 °C for 30 min to completely remove the methanol and some of the water. It was then injected with 0.4 mmol of NaYF_4_ seed particles in a cyclohexane solution. After holding the reaction mix at 110 °C for a further 10 min to evaporate cyclohexane, the reaction mixture was quickly heated to 290 °C and held at that temperature for a further 1 h.

### Synthesis of NaLuF_4_/NaYF_4_ NRs with NaGdF_4_ double-ring

GdCl_3_ (0.1 mmol) in 1 ml methanol solution was magnetically mixed with OA (19.0 mmol) and ODE (18.7 mmol) in a 50-ml three-neck round-bottom flask. The mixture was degassed under Ar flow and heated to 150 °C for 30 min to form a clear solution, and then cooled to room temperature. Methanol solution (2 ml) containing NaOH (at 0.15 mmol) was added and stirred for 60 min. The solution was slowly heated to 110 °C and kept at 110 °C for 30 min to completely remove the methanol and some of the water. It was then injected with 0.1 mmol of the NaYF_4_/NaLuF_4_ seed particles, in a cyclohexane solution, into the reaction solution. After having been held the reaction mix at 110 °C for another 10 min to evaporate cyclohexane, the reaction mixture was quickly heated to 300 °C. It was then, injected with 0.02 mmol of *α*-NaGdF_4_ nanocrystals into the reaction system. This was done every 10 min for 5 times at 300 °C. The reaction mix was held at this temperature for another 10 min after the last injection.

## Additional information

**How to cite this article:** Liu, D. *et al*. Three-dimensional controlled growth of monodisperse sub-50 nm heterogeneous nanocrystals. *Nat. Commun.* 7:10254 doi: 10.1038/ncomms10254 (2016).

## Supplementary Material

Supplementary Information Supplementary Figures 1-35, Supplementary Tables 1-3, Supplementary Notes 1-18, Supplementary Methods and Supplementary References
