# Supplementary material for: Three-dimensional controlled growth of monodisperse sub-50 nm heterogeneous nanocrystals
Source: Nat Commun. 2016 Jan 8;7:10254. doi: 10.1038/ncomms10254 (PMC4729871; doi:10.1038/ncomms10254)
Supplement: Supplementary Information — Supplementary Figures 1-35, Supplementary Tables 1-3, Supplementary Notes 1-18, Supplementary Methods and Supplementary References [file ncomms10254-s1.pdf]

Supplementary Figures

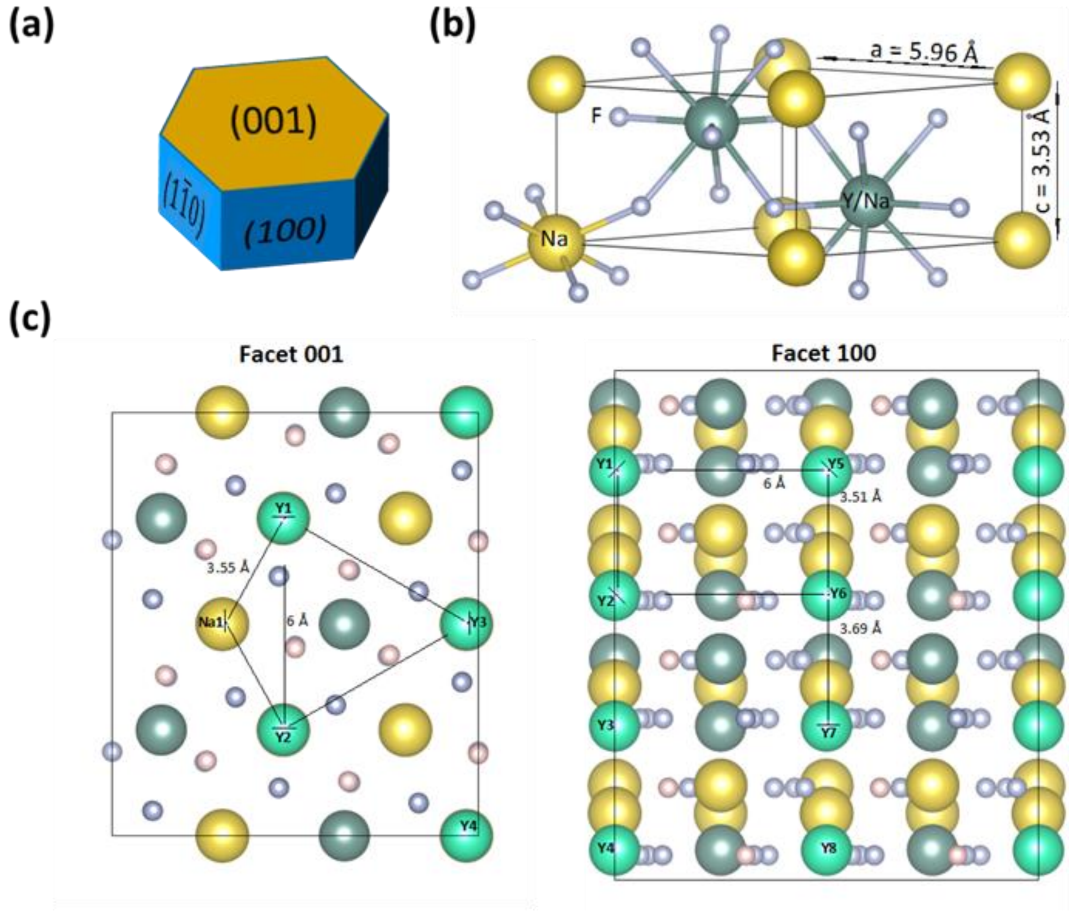

Supplementary Figure 1. Computational models of the crystalline structure of β-$NaYF_4$ facets. (a), the schematic shape of a β-$NaYF_4$ nanocrystal chosen as the core for directional epitaxial growth in this work is illustrated. The hexagonal cylinder consists of the (001) facets at the ends and identical (100) (010) and (1$\bar{1}$0) facets around the cylinder sides. (b), A unit cell of β-$NaYF_4$ structure with detailed atomic arrangement and the lattice parameters; (c), The relaxed atomic configurations of the most stable (001) and (100) facets. The Y^{3+} atoms form equilateral triangles with a length of 6 Å in the relaxed (001) surface, and rectangles are observed in the (100) surface with a shorter length of 3.51 or 3.69 Å; The light green and pink balls represent surface Y^{3+} and F^- atoms, respectively.

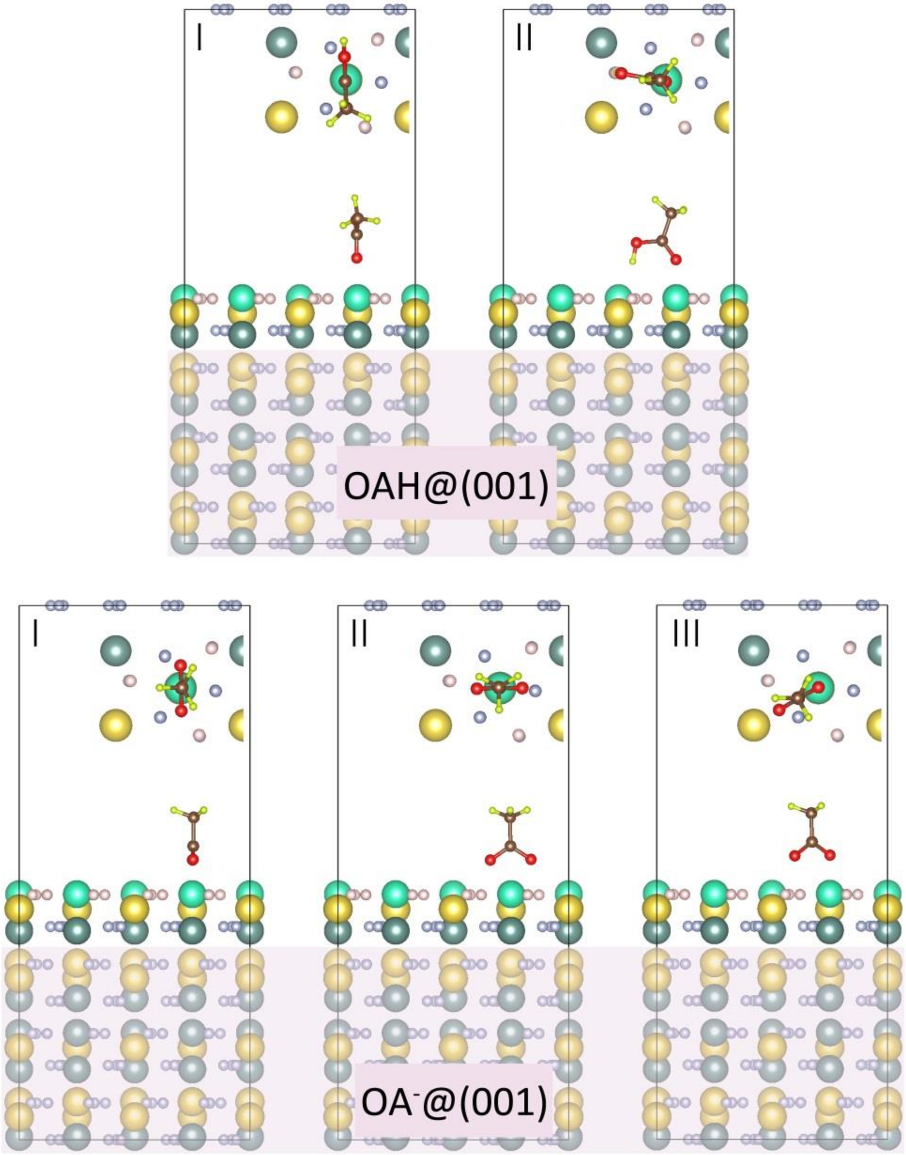

Supplementary Figure 2. Adsorption models of OAH and OA^- ligands on the (001) surface with different initial configurations. Top-view configuration is shown as an inset. The shadowed area represents the fixed atomic layers. The color of the atoms are: Na atoms: yellow, Y atoms: cyan, F atoms: white, C atoms: brown, O atoms: red and H atoms: light yellow. The light green and pink spheres represent the surface Y and F atoms, respectively.

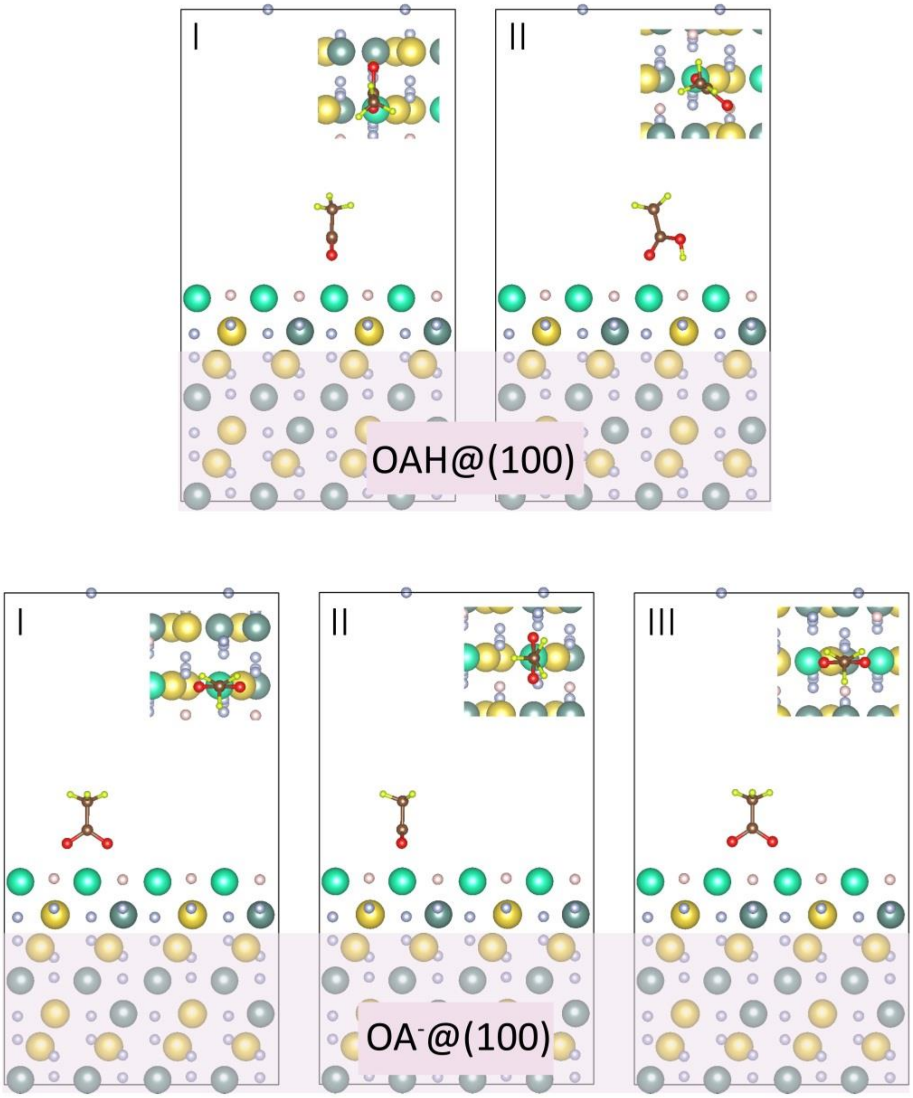

Supplementary Figure 3. Adsorption models of OAH and OA$^-$ ligands on the (100) surface with different initial configurations. Top-view configuration is shown as an inset. The shadowed area represents fixed atomic layers. The color of the atoms are: Na atoms: yellow, Y atoms: cyan, F atoms: white, C atoms: brown, O atoms: red and H atoms: light yellow. The light green and pink spheres represent surface Y and F atoms, respectively.

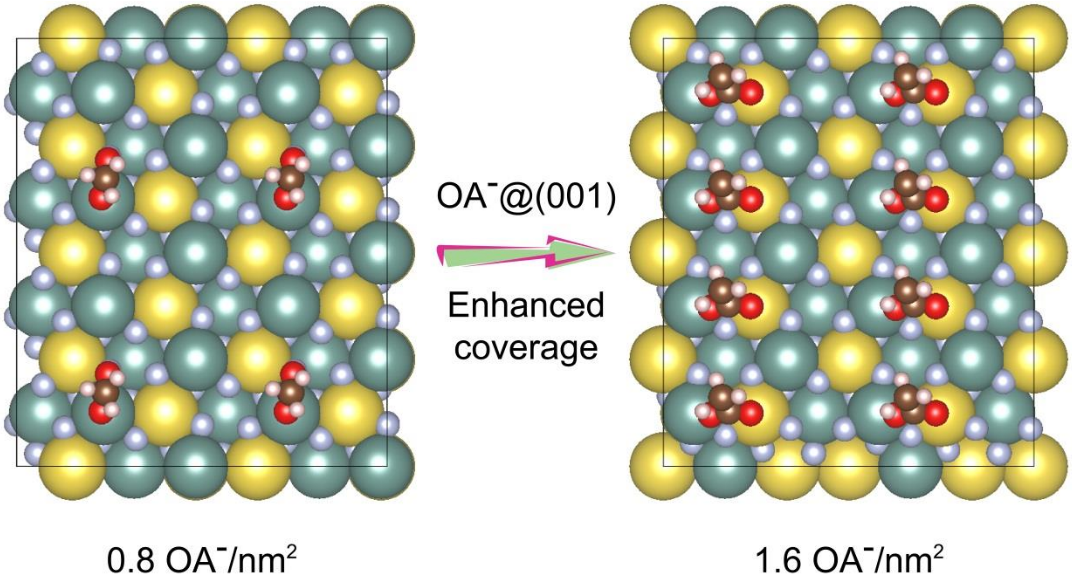

Supplementary Figure 4. Adsorption model of OA^- ligands on the (001) surface with different ligand coverage. The color of the atoms are: Na atoms: yellow, Y atoms: cyan, F atoms: white, C atoms: brown, O atoms: red and H atoms: pink.

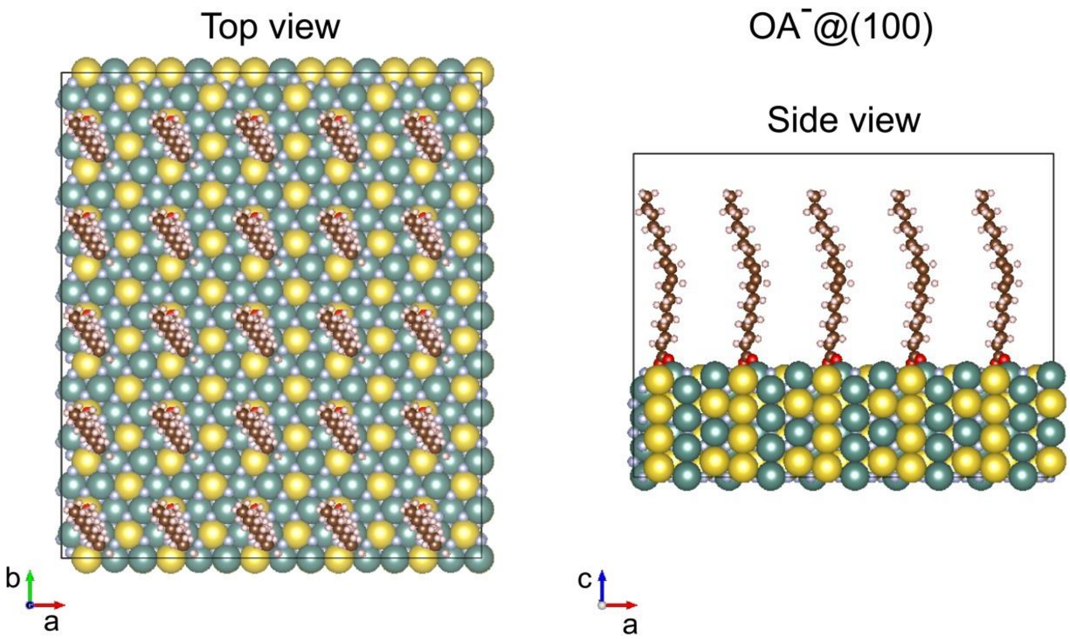

Supplementary Figure 5. Adsorption model of real OA^- ligands (18 C atoms) on the (100) surface. The color of the atoms are: Na atoms: yellow, Y atoms: cyan, F atoms: white, C atoms: brown, O atoms: red and H atoms: pink.

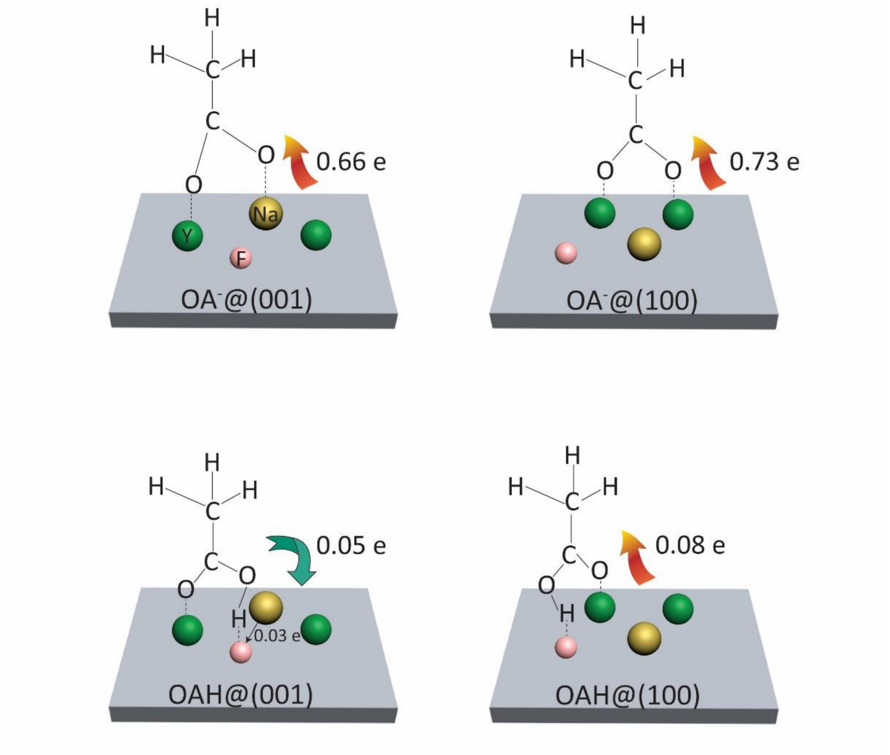

Supplementary Figure 6. Schematic description of the charge transfer between ligands and surfaces. The details of respective mechanisms are shown in Supplementary Table 1. When the OA^- ions approach the exposed Y^{3+} ions they bind to the Y-Y and Y-Na pairs on the (100) and (001) surfaces, respectively. The charge analysis revealed that the Y atoms located on the (100) surface have about 0.35 electrons per atom less saturated than the same atoms located on the (001) surface, although Y ions on both surfaces have the same 6-fold coordination. Therefore, the charge can be easily transferred from the (100) surface to OA^- through a pair of Y-O bonds, giving rise to higher binding strength on that surface compared to (001). In the case of OAH molecule the binding mechanism is less straightforward since the OAH molecules bind to Y-F pairs at both (001) and (100) surfaces. However, our charge analysis reveals that the OAH molecules donate electrons to fluorine atoms located on the (001) surface, but not to the same atoms on the (100) surface. Moreover, adsorption of OAH onto the (001) surface induces a charge transfer from the surface Na atoms to F atoms, resulting in a self-saturation. These two separate modes of charge transfer enhance the stability of the capped system for the (001) orientation. On the other hand, when the OAH approaches the (100) surface, the charge is transferred from the surface F/Y atoms to OAH. In this case, the stability of the capped system deteriorates because the F atoms accept rather than donate electrons.

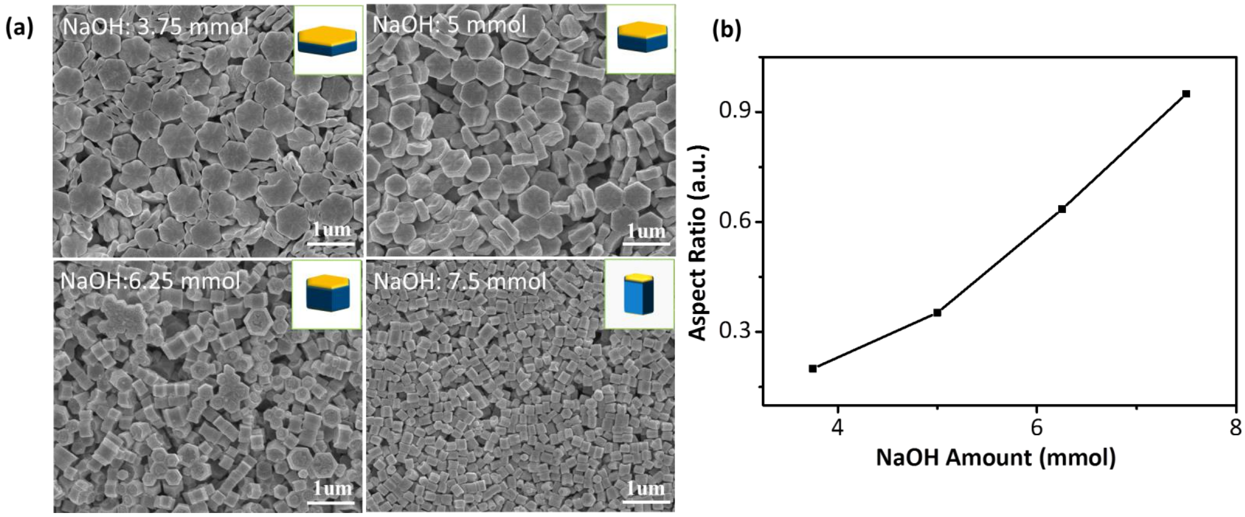

Supplementary Figure 7. Micron-sized $NaYF_4$ crystals. (a) SEM images of the $NaYF_4$ disks and rods synthesized under hydrothermal conditions and with different amounts of NaOH (Scale bars: 1 μm). (b) The observed aspect ratio of $NaYF_4$ crystals is plotted against the amount of NaOH used in the reaction mixture.

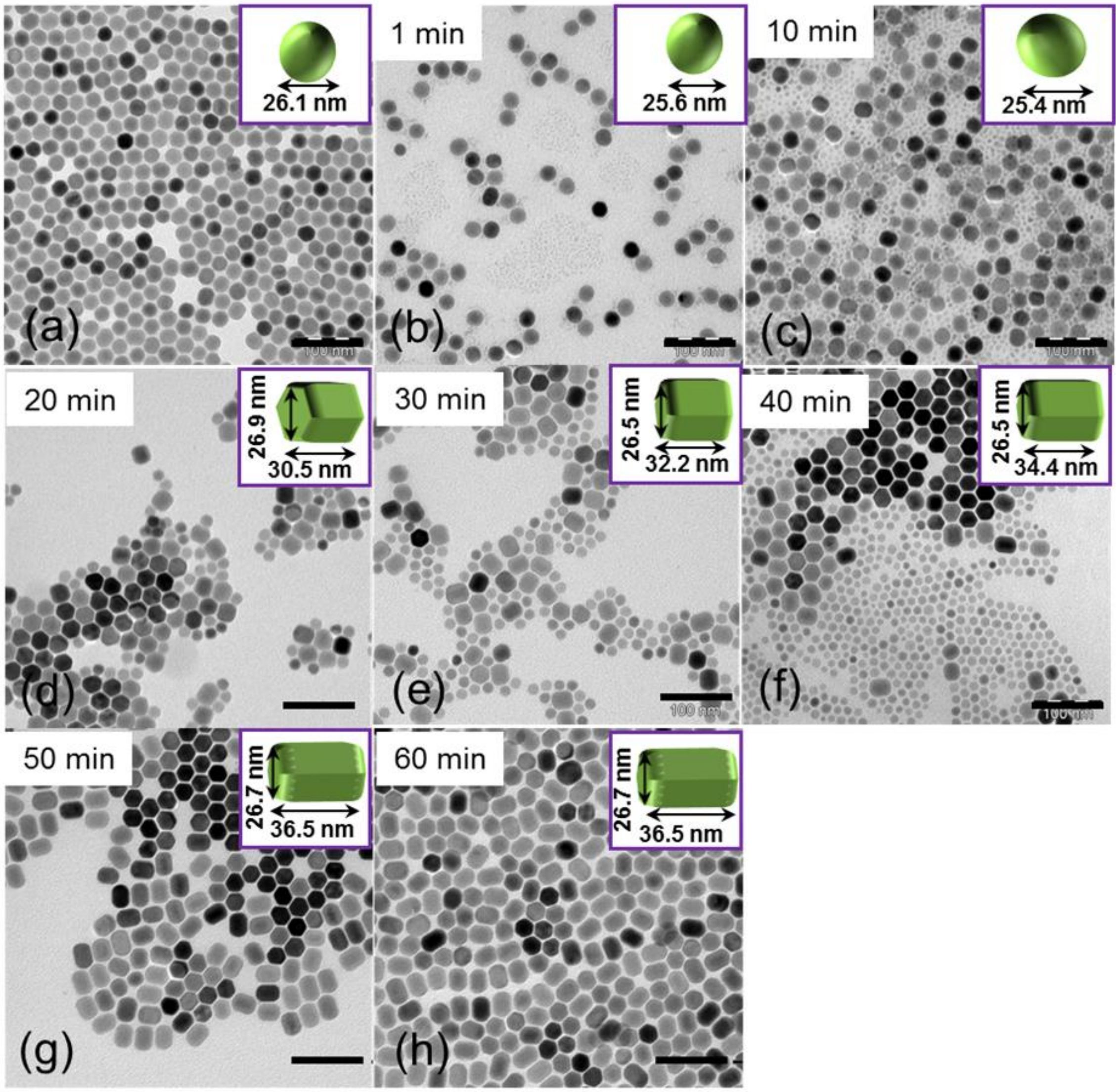

Supplementary Figure 8. Evolution of longitudinal growth $NaYF_4$ nanorods. Real-time monitoring of the epitaxial growth process of crystals from their $NaYF_4$ nanocrystal cores to their longitudinally form as $NaYF_4$ nanorods. TEM images of the $NaYF_4$ core (a) and their products after step-by-step sampling at after 1 minute (b), 10 minutes (c), 20 minutes (d), 30 minutes (e), 40 minutes (f), 50 minutes (g) and 60 minutes (h) of reaction time (Scale bars: 100 nm). The TEM characterization confirmed that the epitaxial growth of shells onto the core was initiated by the formation of α-$NaYF_4$ nanocrystals at the beginning of the epitaxial growth, before these transformed into the stable β-$NaYF_4$ nanocrystals.

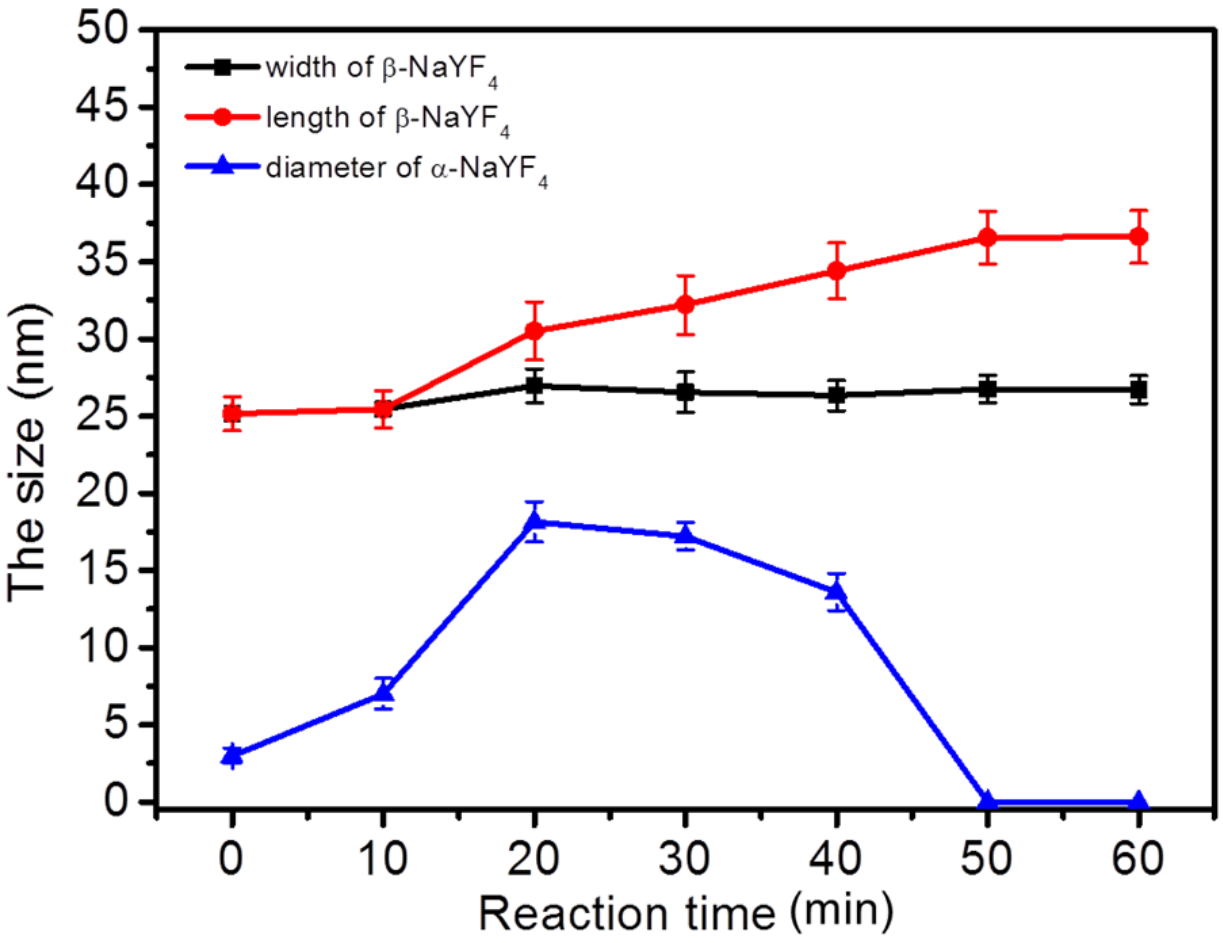

Supplementary Figure 9. Size evolution of both α phase $NaYF_4$ and β-$NaYF_4$ nanorods. The plot shows size evolution of the diameter of the α phase $NaYF_4$ nanocrystals and the length and width of β-$NaYF_4$ nanorods as a function of their reaction time from 0 minutes to 60 minutes. The size of α-$NaYF_4$ nanocrystals increased for the first 20 minutes, and decreased for the following 30 minutes until they completely disappeared after 50 minutes of the reaction. The dissolved α phase $NaYF_4$ nanocrystals released Na, Y and F sources, which was used for the epitaxial growth of the β-$NaYF_4$ cores. At the same time, the length of β-$NaYF_4$ cores gradually increases during the whole reaction process from 25 nm to 35 nm while the width of β-$NaYF_4$ nanocrystals remains constant at about 25-27 nm.

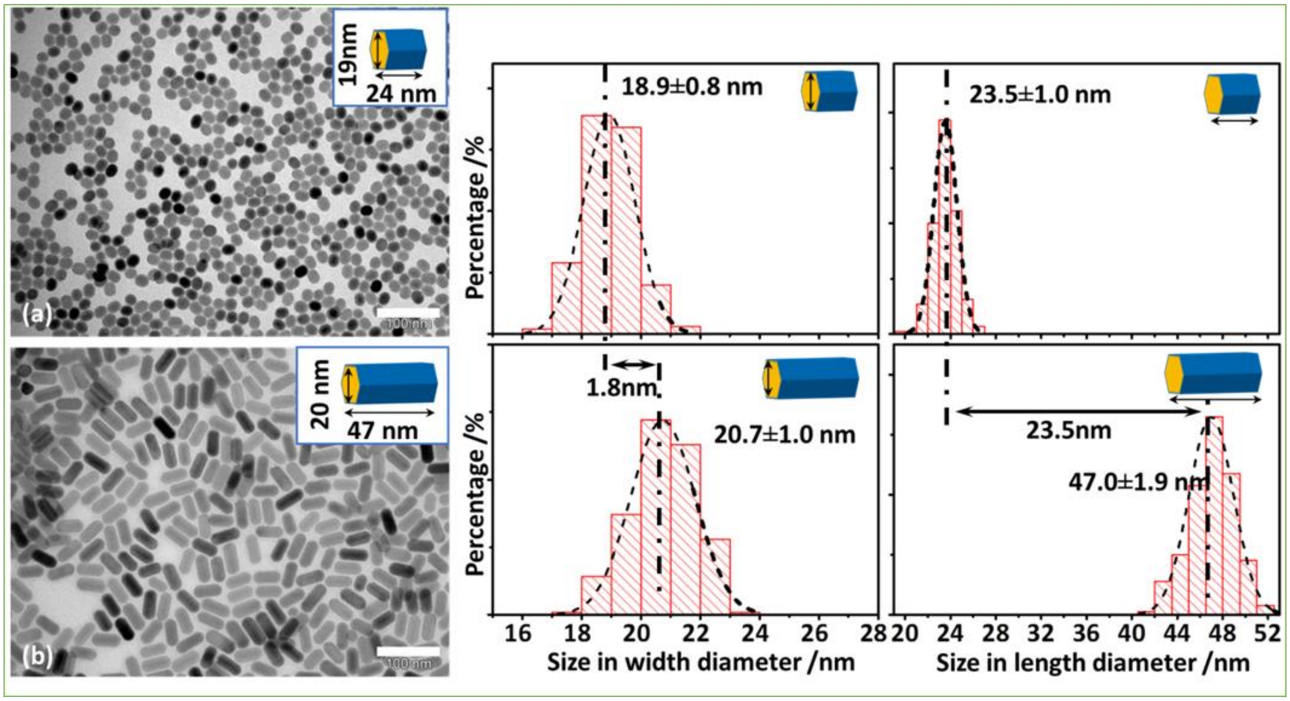

Supplementary Figure 10. Longitudinal growth of $NaYF_4$ nanorods. TEM images and size distributions of the $NaYF_4$ core nanocrystals (a) and the $NaYF_4$ nanorods obtained from a longitudinal epitaxial shell growth process (b). The size of the $NaYF_4$ markedly increases in the longitudinal direction after the secondary growth of $NaYF_4$ shell from 24 nm to 47 nm. This was accompanied by a slight increase (1.8 nm) in their transversal dimension. (Scale bar is 100 nm) This figure further illustrates that mono-disperse nanorods with high aspect ratio (>2) can be stepwise synthesized by secondary epitaxial growth in a high concentration of oleate ions when reacted at high temperature by the co-precipitation method.

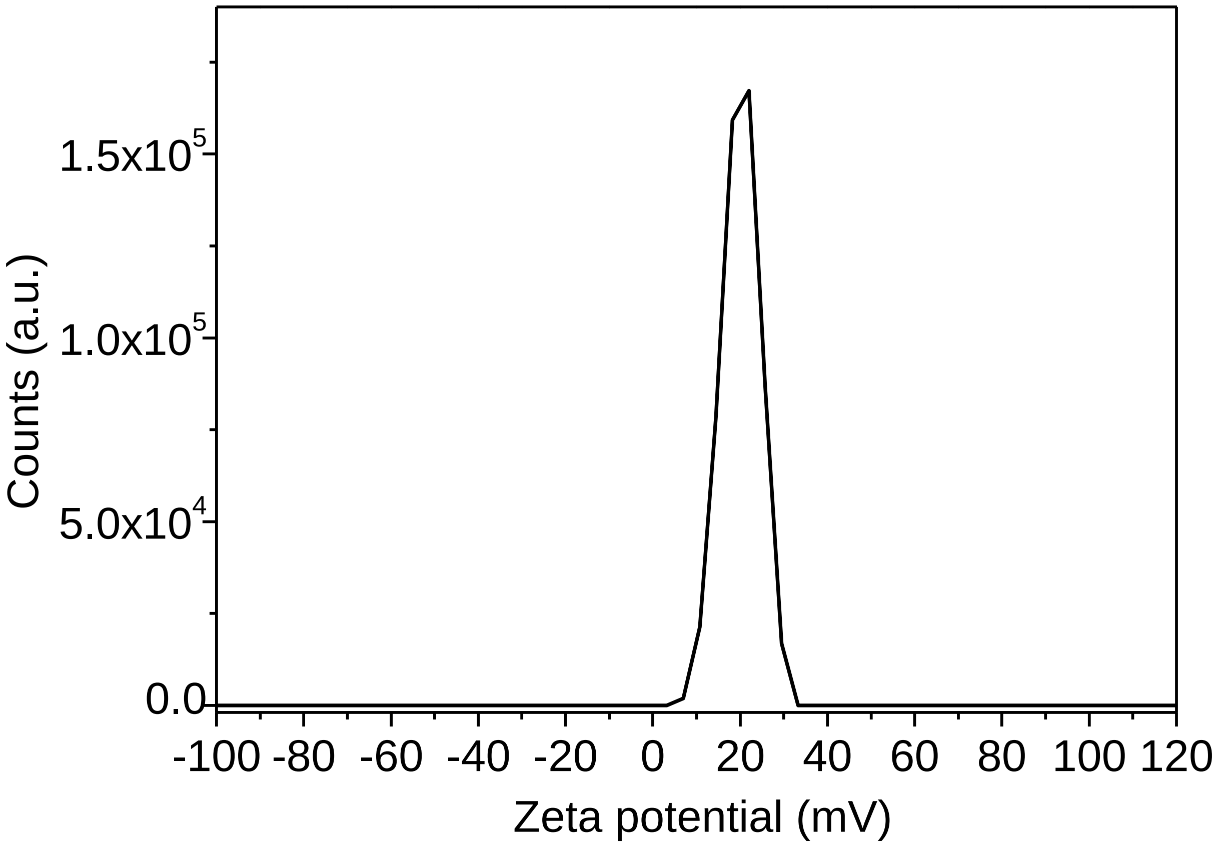

Supplementary Figure 11. Zeta potential graph of ligand-free $NaYF_4$ nanocrystals. The hydrophobic nanocrystals become hydrophilic in water after treatment with diluted HCl solution and after ultrasonication. This indicates that the strongly binding oleate ions (OA^-) reacts with H^+ to form OAH at a lower binding energy, and is removable by the ultrasonic treatment. After both OAH molecules and OA^- Oleate ions have been removed from the crystal facets, the zeta potential of ligand free $NaYF_4$ nanocrystals in MQ water was about +20 mV, which suggests that the naked nanocrystals are positively charged due to exposed rare earth ions on their surface.

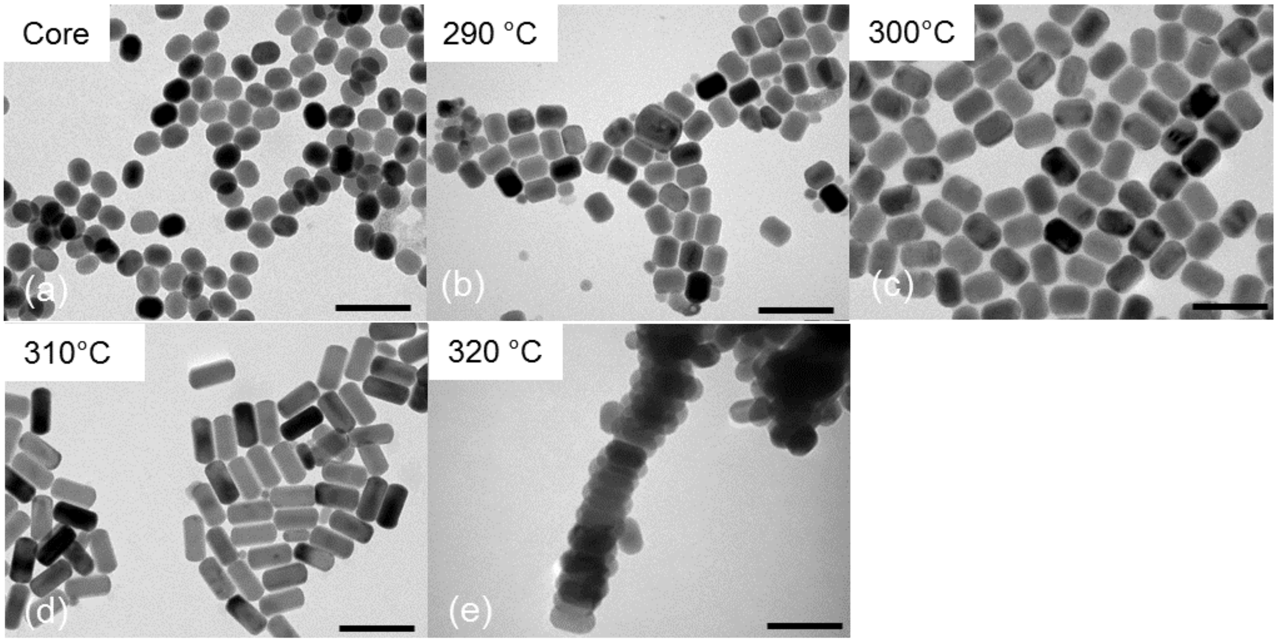

Supplementary Figure 12. Investigating the reaction temperature. TEM images of $NaYF_4$ core nanocrystals (a) and related core-shell nanocrystals synthesized at the high ratio of OA^-/OAH and at the temperature of 290 ˚C (b), 300 ˚C (c), 310 ˚C (d) and 320 ˚C (e). Note, the reaction times were 1 hour for each experiment. (Scale bar: 100 nm)

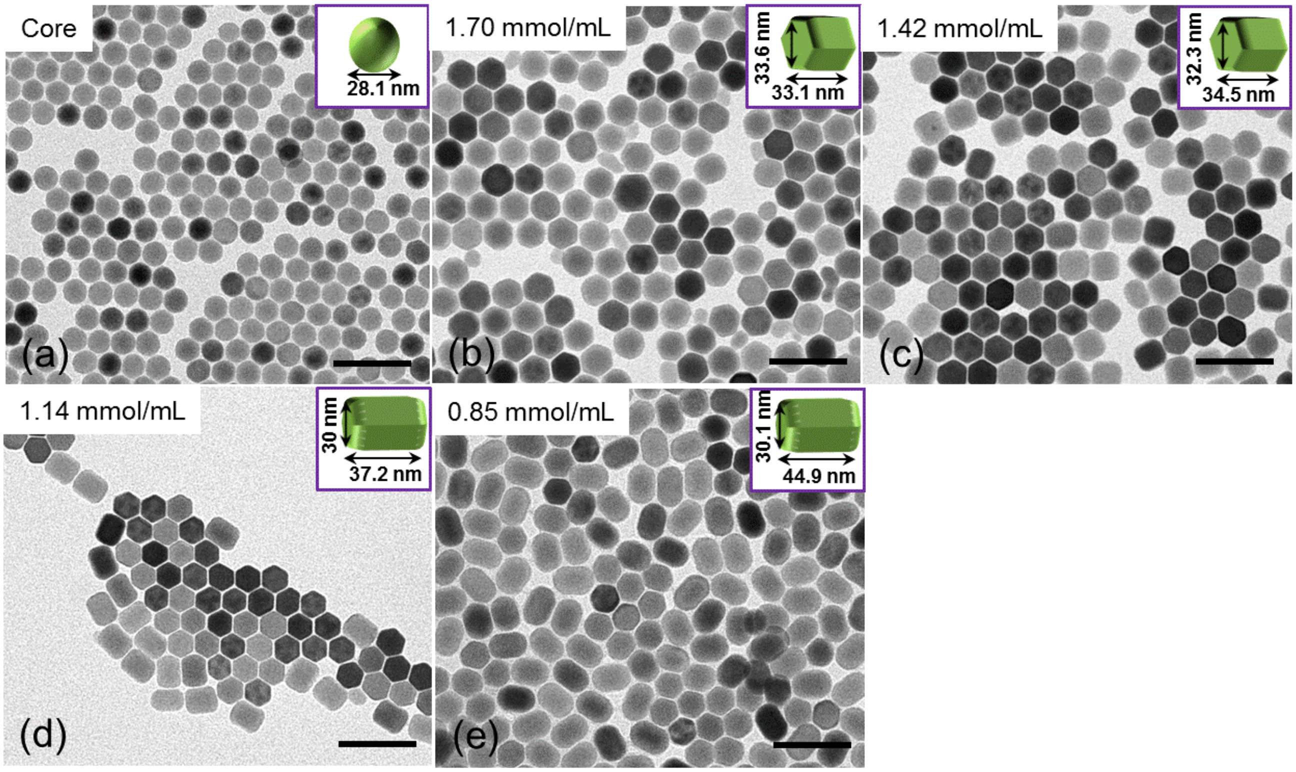

Supplementary Figure 13. Investigating the role of OA concentration. TEM images of $NaYF_4$ core nanocrystals (a) and corresponding core-shell nanocrystals synthesized under OA (including OA^- and OAH) concentration of 1.70 mmol/mL (b), 1.42 mmol/mL (c), 1.14 mmol/mL (d), 0.85 mmol/mL (e). The remaining reaction conditions for the longitudinal growth process were identical. (Scale bar: 100 nm)

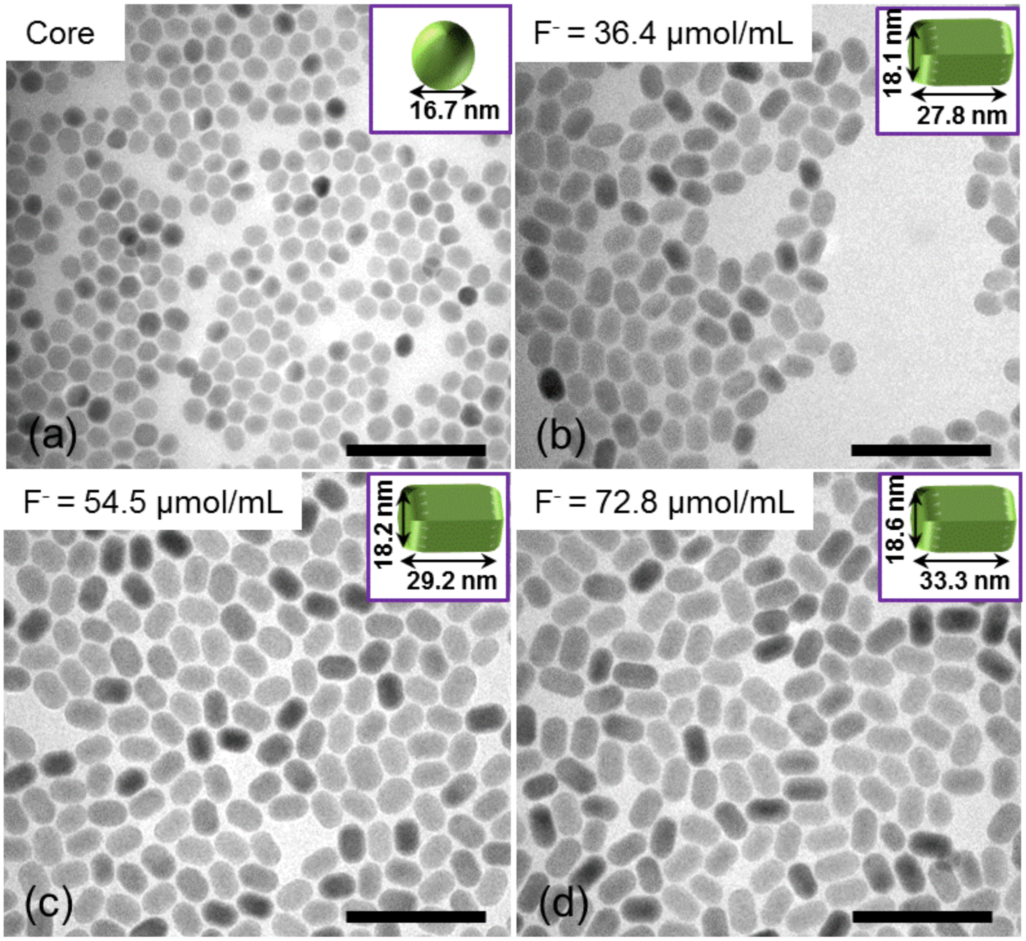

Supplementary Figure 14. **Investigating the role of F^- concentration.** TEM images of $NaYF_4$ core only nanocrystals (a) and nanocrystals grown with $NaYF_4$ shells at varied the concentration of F^-, 36.4 µmol/mL (0.4 mmol) (b), 54.5 µmol/mL (0.6 mmol) (c) and 72.8 µmol/mL (0.8 mmol) (d) The remaining reaction conditions were kept the same as in the longitudinal growth process. (Scale bar: 100 nm)

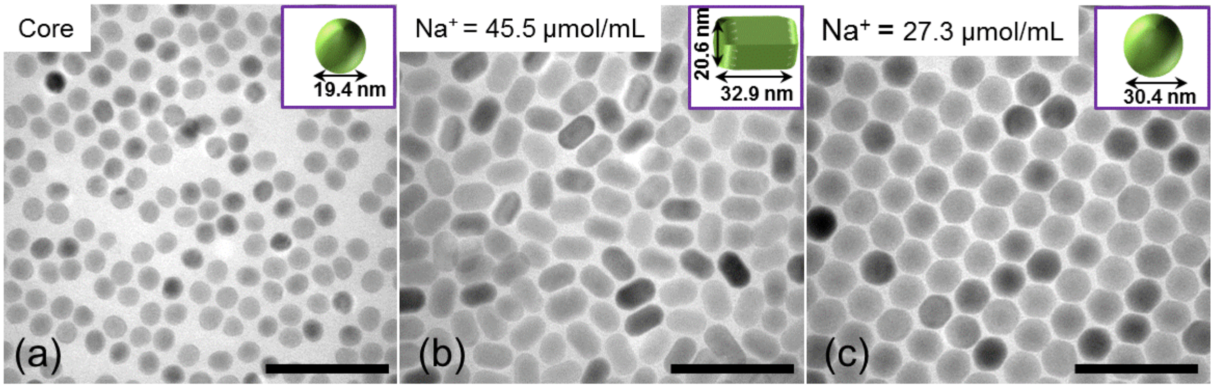

Supplementary Figure 15. Investigating the role of Na^+ concentration. TEM images of $NaYF_4$ core-only nanocrystals (a) and grown with $NaYF_4$ shells at two different concentration of Na^+, 45.5 μmol/mL (NaOH=0.5 mmol) (b) and 27.3 μmol/mL (NaOH=0.3 mmol) (c). The remaining reaction conditions were kept the same as those used in the longitudinal growth process. (Scale bar: 100nm)

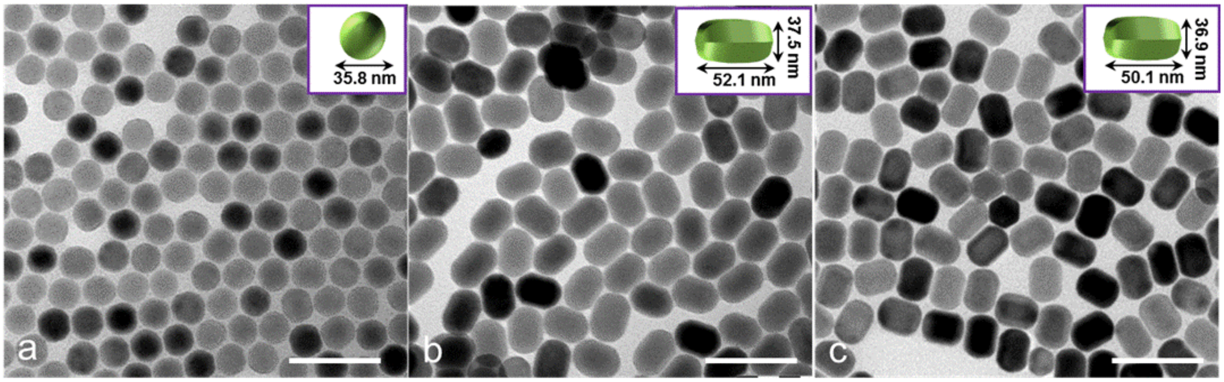

Supplementary Figure 16. Ruling out OH^- as a key factor for longitudinal growth. TEM images of $NaYF_4$ core (a) and $NaYF_4$ coated with $NaYF_4$ shell using NaOA as a sodium source (b) and $NaYF_4$ nanorods synthesized with NaOH as a sodium source (c). (Scale bar = 100 nm) Identical results were obtained with $NaYF_4$ shell grown longitudinally onto the $NaYF_4$ core regardless whether OH^- was present in the reaction or not, thus ruling out OH^- as a key factor for longitudinal growth in the reaction system described here.

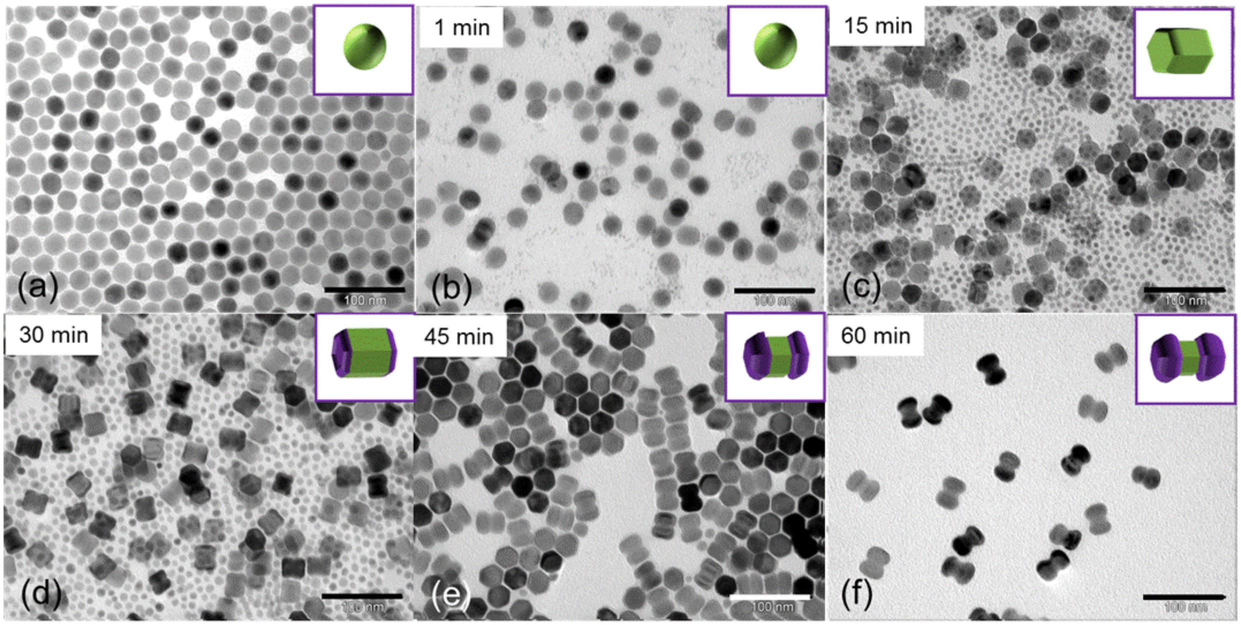

Supplementary Figure 17. **Morphology evolution of longitudinal growth of $NaGdF_4$ shell onto $NaYF_4$ cores**. The TEM images of the $NaYF_4$ cores (a) and the evolution of its heterogeneous products are shown by step-by-step sampling during the reaction at 310˚C after 1 min (b), 15 min (c), 30 min (d), 45 min (e), 60 min (f) of reaction time (scale bars: 100 nm)

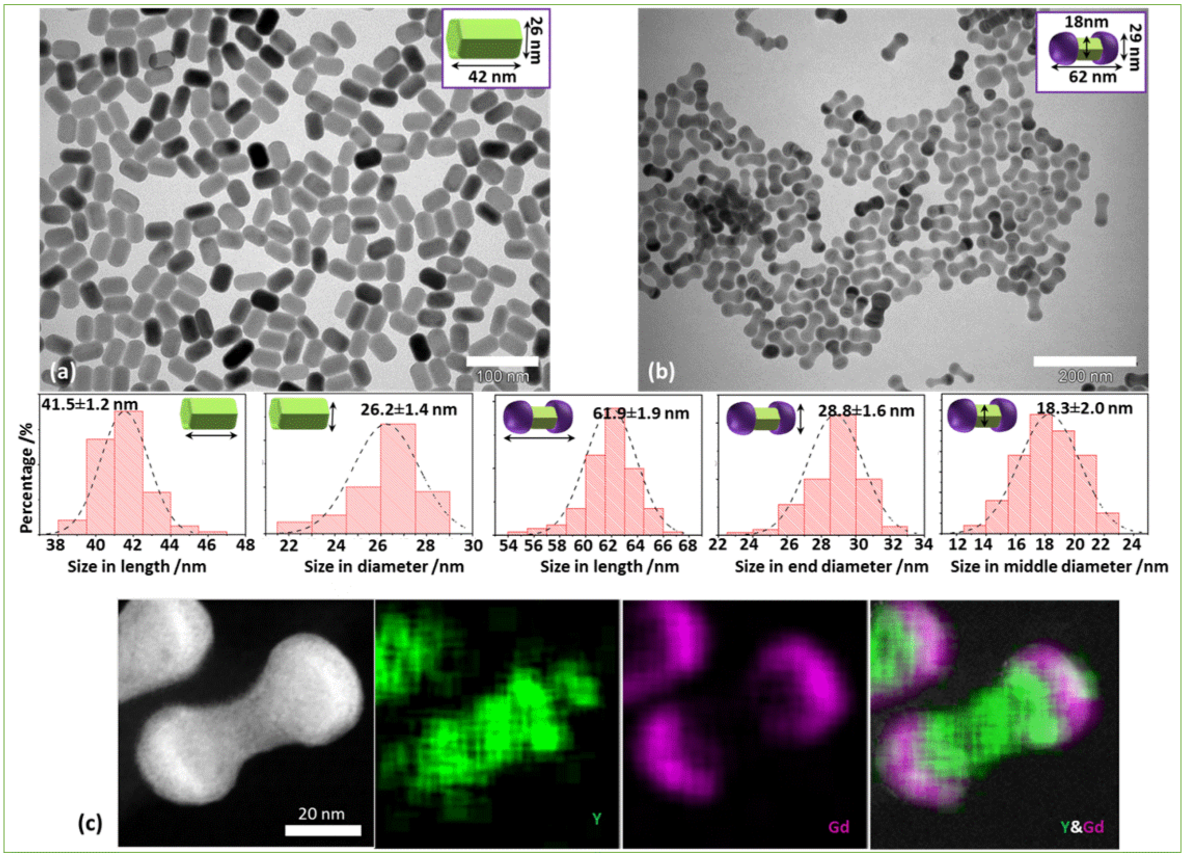

Supplementary Figure 18. $NaGdF_4$/$NaYF_4$ nano-dumbbells. TEM images, size distributions and characterization of elemental compositions of heterogeneous growth of $NaGdF_4$ onto the $NaYF_4$ core nanocrystals. The (a) $NaYF_4$ core nanocrystals; and (b) the $NaGdF_4$/$NaYF_4$ nanocrystals after epitaxial growth of $NaGdF_4$ in the longitudinal direction. The (c) HAADF-STEM image of a single dumbbell shaped $NaYF_4$/$NaGdF_4$ nanocrystal is shown with its elemental mapping images. (Scale bars:100 nm in (a) and (b), 20 nm in (c))

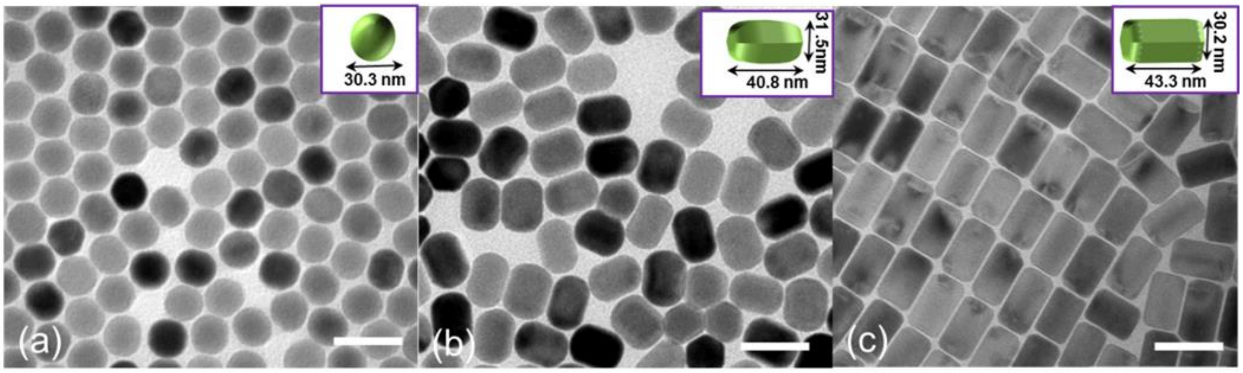

Supplementary Figure 19. $NaYF_4$ nanorods by adding KOH. TEM images of the $NaYF_4$ core (a) and the $NaYF_4$ coated with a $NaYF_4$ shell without KOH present (b) and with 0.4 mmol of KOH present (c) (Scale bars: 50 nm)

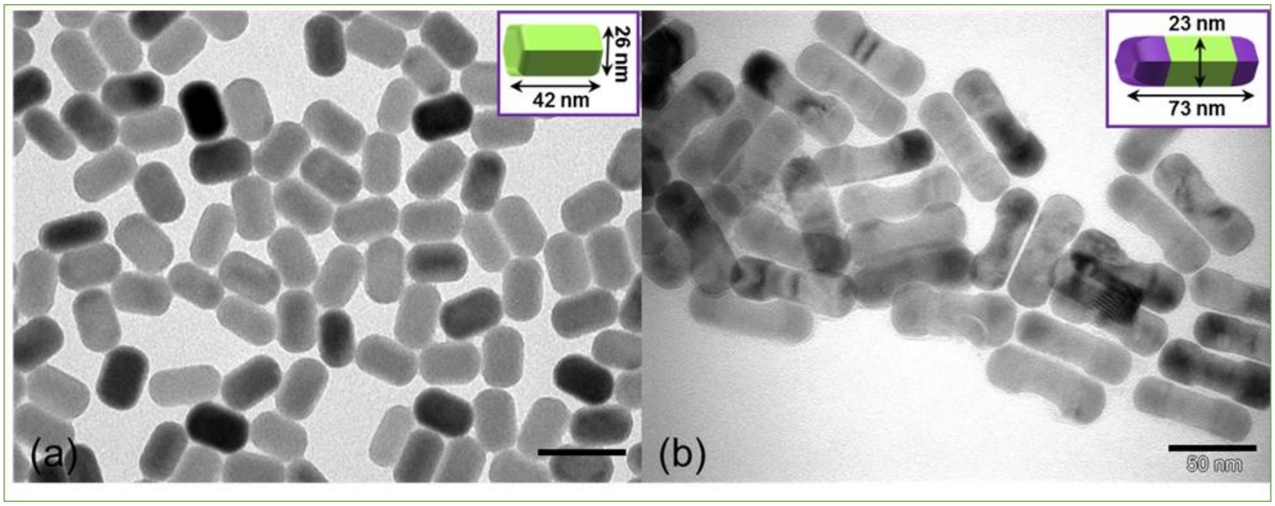

Supplementary Figure 20. **$NaGdF_4$/$NaYF_4$ nanorods by adding KOH.** TEM images of the $NaYF_4$ nanorods acting as the core (a) and the longitudinal growth of the $NaGdF_4$ shell onto the $NaYF_4$ core with 0.4 mmol KOH present in the mix. (Scale bars: 50 nm)

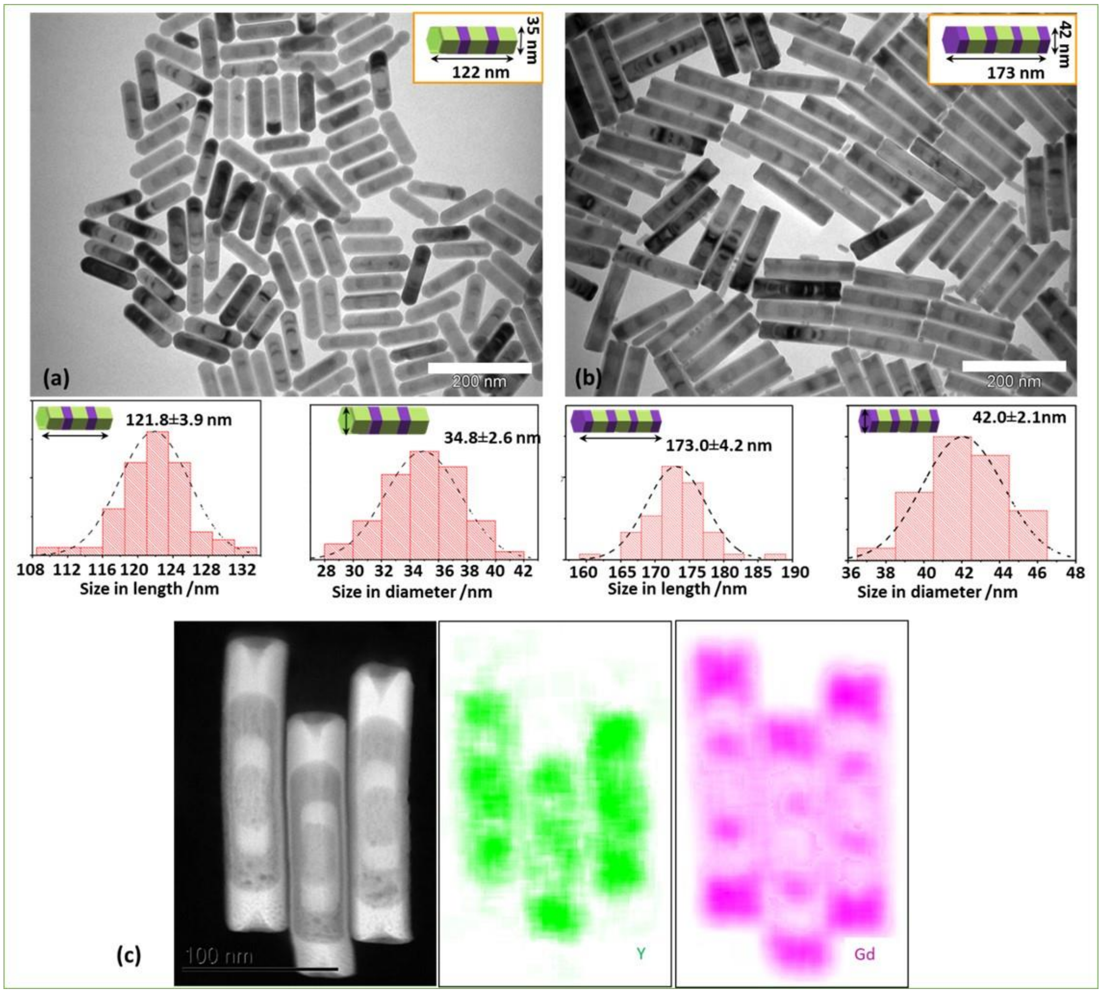

Supplementary Figure 21. $NaYF_4$/$NaGdF_4$ bamboo-like nanorods. TEM images, size distribution and characterization of the elemental composition of periodical $NaYF_4$/$NaGdF_4$ nanocrystals which form a bamboo-like nanostructure. (a) five-section $NaYF_4$/$NaGdF_4$ nanocrystals in a bamboo shape obtained by an epitaxial growth of $NaYF_4$ and $NaGdF_4$ in a longitudinal direction; (b) seven-section $NaYF_4$/$NaGdF_4$ nanocrystals in an bamboo shape; the length of the five-section $NaYF_4$/$NaGdF_4$ nanocrystals can reach 122 nm and its width, 35 nm. With two more sections, the seven-section $NaYF_4$/$NaGdF_4$ nanocrystals grow to 173 nm and the width increases to 42 nm. (c) HAADF-STEM image of three single $NaYF_4$/$NaGdF_4$ nanocrystals and their elemental mapping images. The HAADF-STEM image demonstrates the contrast in density between the $NaGdF_4$ and the $NaYF_4$ as well as their elemental corresponding mapping images, which confirm the distribution of the elements Y and Gd. (scale bars: 200 nm in (a) and (b), 100 nm in (c))

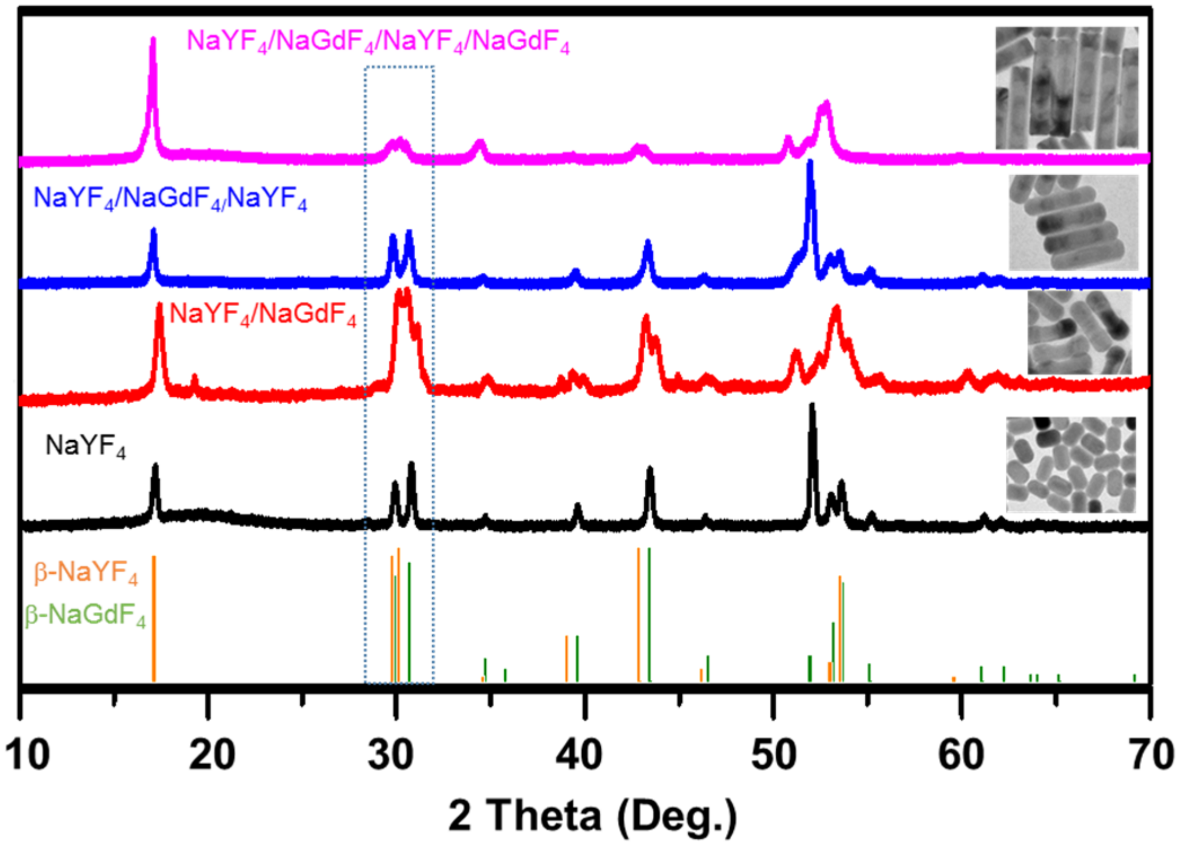

Supplementary Figure 22. The XRD pattern of $NaYF_4$ and different $NaYF_4/NaGdF_4$ nanorods. The XRD for the β-$NaYF_4$ nanorods, $NaYF_4/NaGdF_4$ nanorods, $NaYF_4/NaGdF_4/NaYF_4$ nanorods and the $NaYF_4/NaGdF_4/NaYF_4/NaGdF_4$ nano-bamboos. The figure shows that all these $NaYF_4/NaGdF_4$ hetero nanocrystals were identified as hexagonal phase when compared to the β-$NaYF_4$: JCPDS:16-0334 and β-$NaGdF_4$: JCPDS: 27-0699 file shown). As the marked peaks shown, the two peaks of the $NaYF_4$ and $NaGdF_4$ at approximately 30 degrees, overlap to form a broad peak, which suggests that the nanocrystals contain both β-$NaYF_4$ and β-$NaGdF_4$

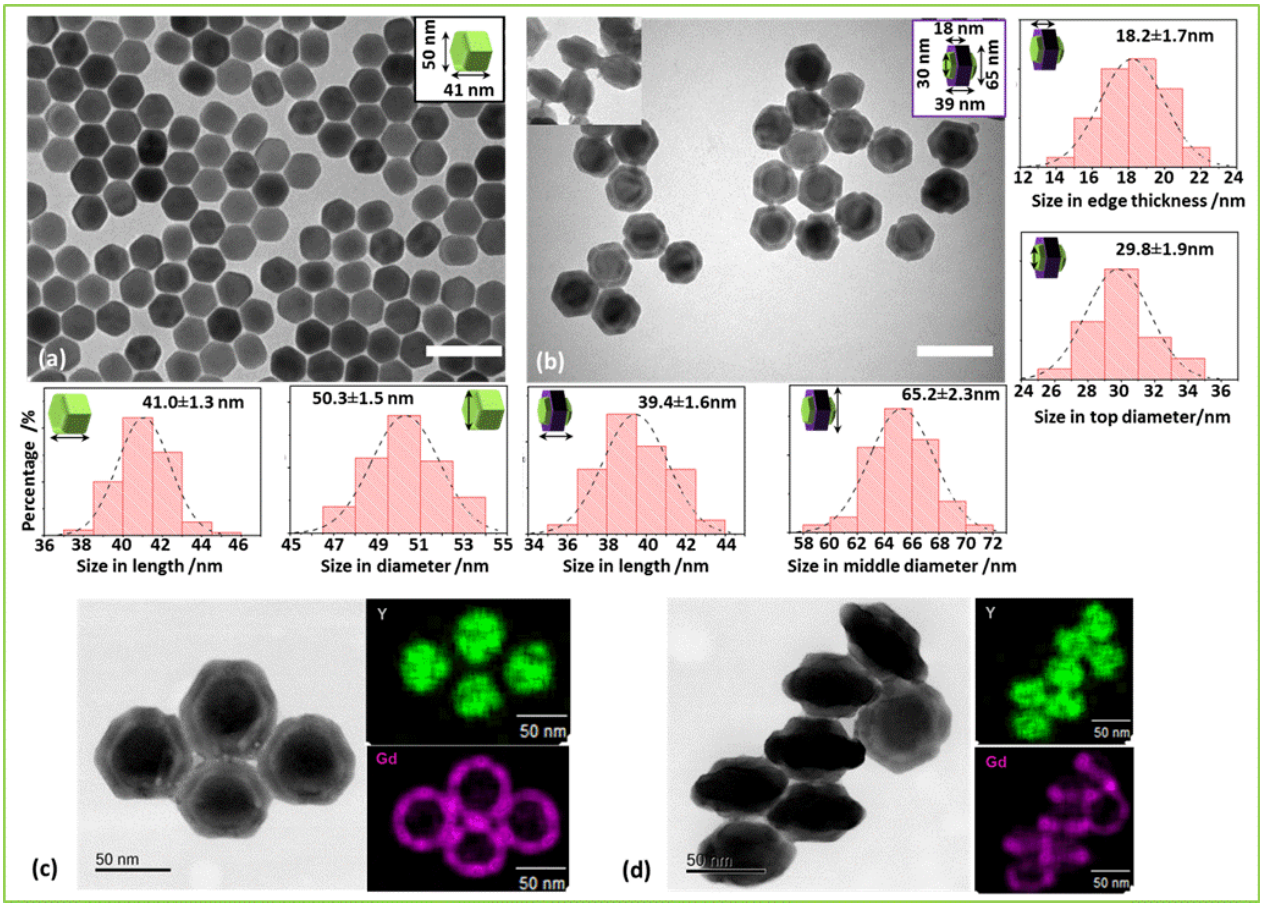

Supplementary Figure 23. Transversal growth of $NaGdF_4$ shell onto $NaYF_4$ core. (a-b): TEM images and size distribution histograms. (a): the $NaYF_4$ core nanocrystals (scale bar is 100 nm). b): the $NaYF_4$-$NaGdF_4$ nanocrystals with transversal growth of $NaGdF_4$ on their side surfaces (scale bar is 100 nm).

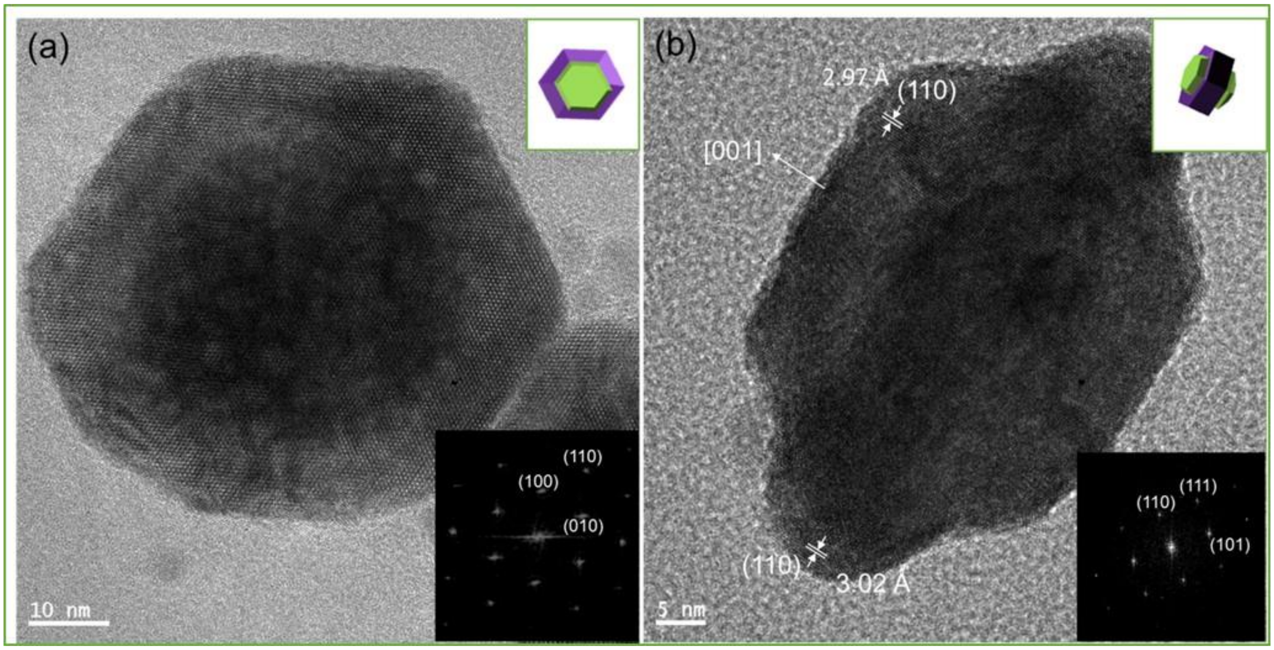

Supplementary Figure 24 Transversal growth of $NaGdF_4$ shell onto $NaYF_4$ core. (a) Top view high-resolution TEM image of a single nanocrystal and its corresponding selected-area electron diffraction pattern (inset) taken in the [001] incidence and in (b) the side view high-resolution TEM image and its corresponding selected area electron diffraction pattern as an insert. (Scale bars: 5 nm)

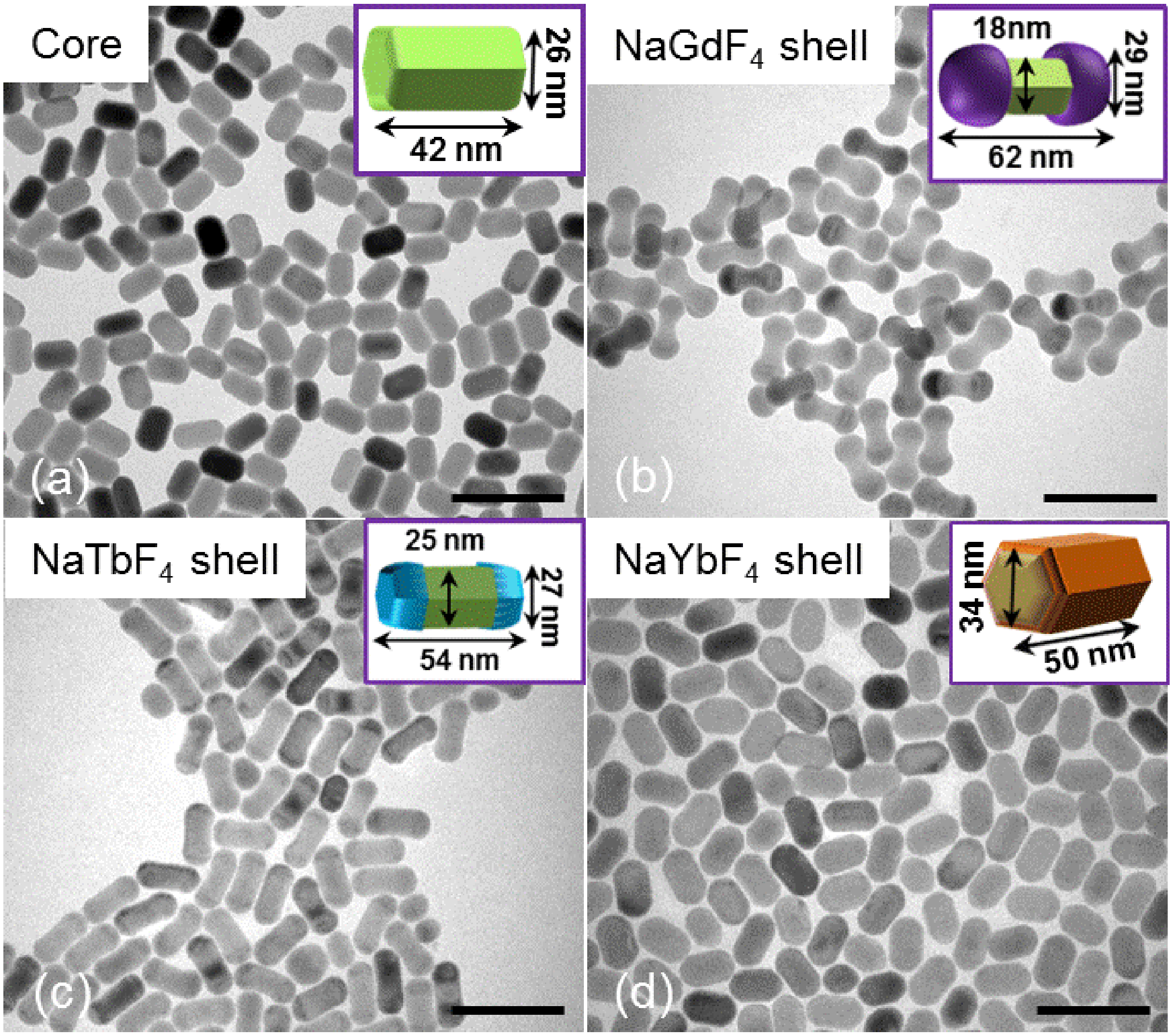

Supplementary Figure 25 Investigating the stability of shell material and its effect on crystal dissolution. TEM images of $NaYF_4$ core only nanocrystals (a) and grown with different $NaLnF_4$ shells, $NaGdF_4$ (b), $NaTbF_4$ (c) and $NaYbF_4$ (d).

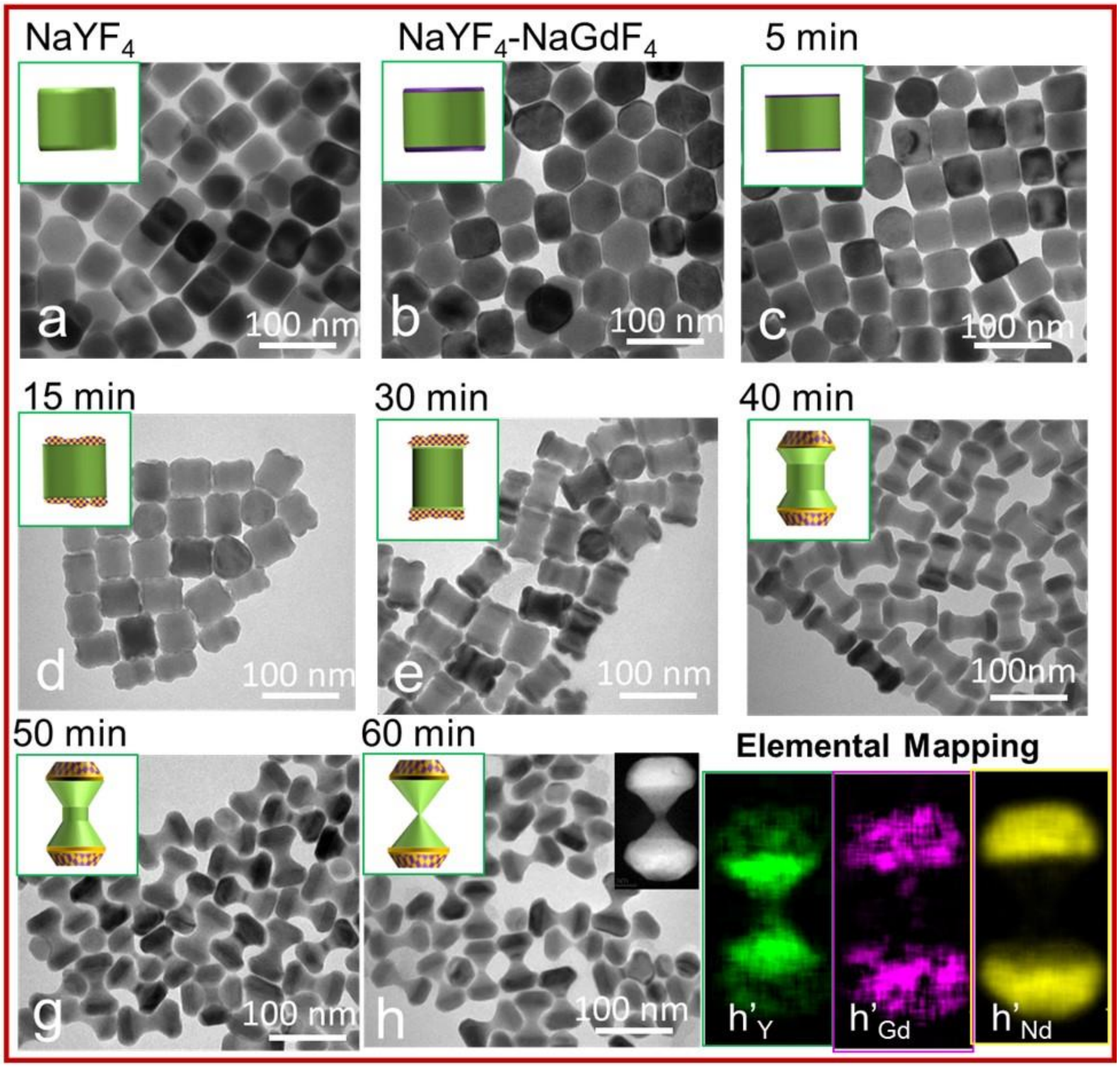

Supplementary Figure 26. **Real-time monitoring the migration growth process of $NaYF_4/NaGdF_4/NaNdF_4$NCs.** TEM images of $NaYF_4$ nanocrystal (a), $NaYF_4/NaGdF_4$ core-shell nanocrystals (b), the migration growth after 5 minutes (c), 15 minutes (d), 30 minutes (e), 40 minutes (f), 50 minutes (g), 60 minutes (h) and the elemental mapping of Y(h'$_{Y}$), Gd (h'$_{Gd}$) and Nd (h'$_{Nd}$) elements.

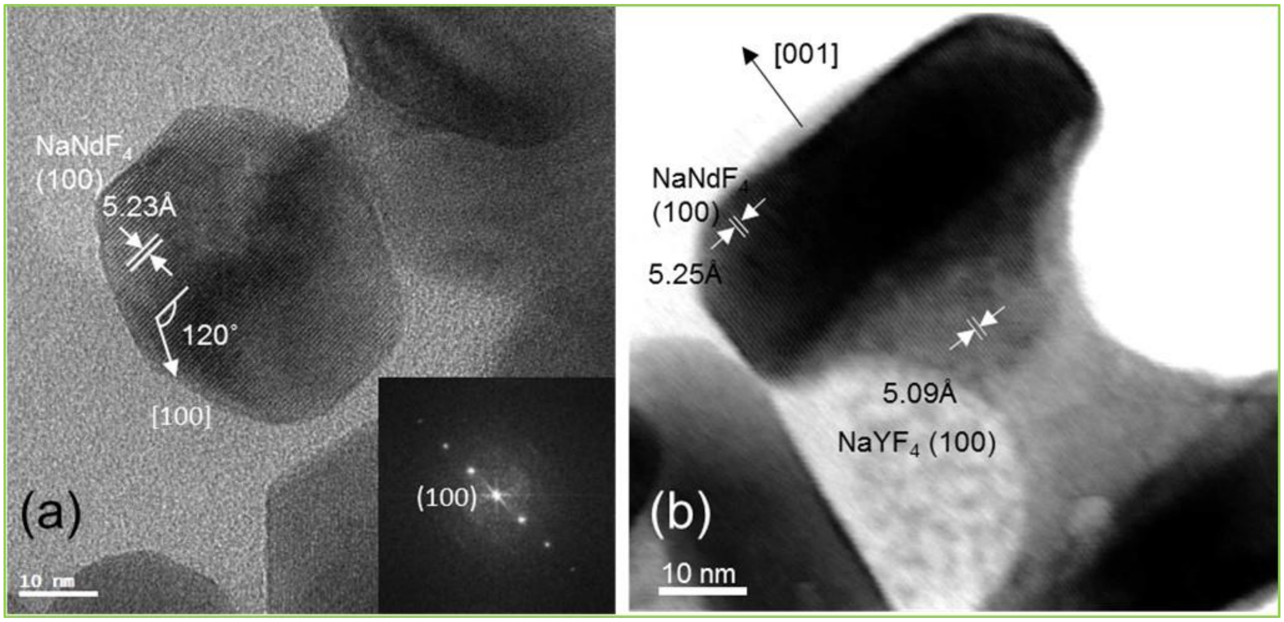

Supplementary Figure 27. High-resolution TEM images of nanoscale hourglass. (a) The top view and (b) the side view of the nano-hourglass. (Scale bars: 10 nm)

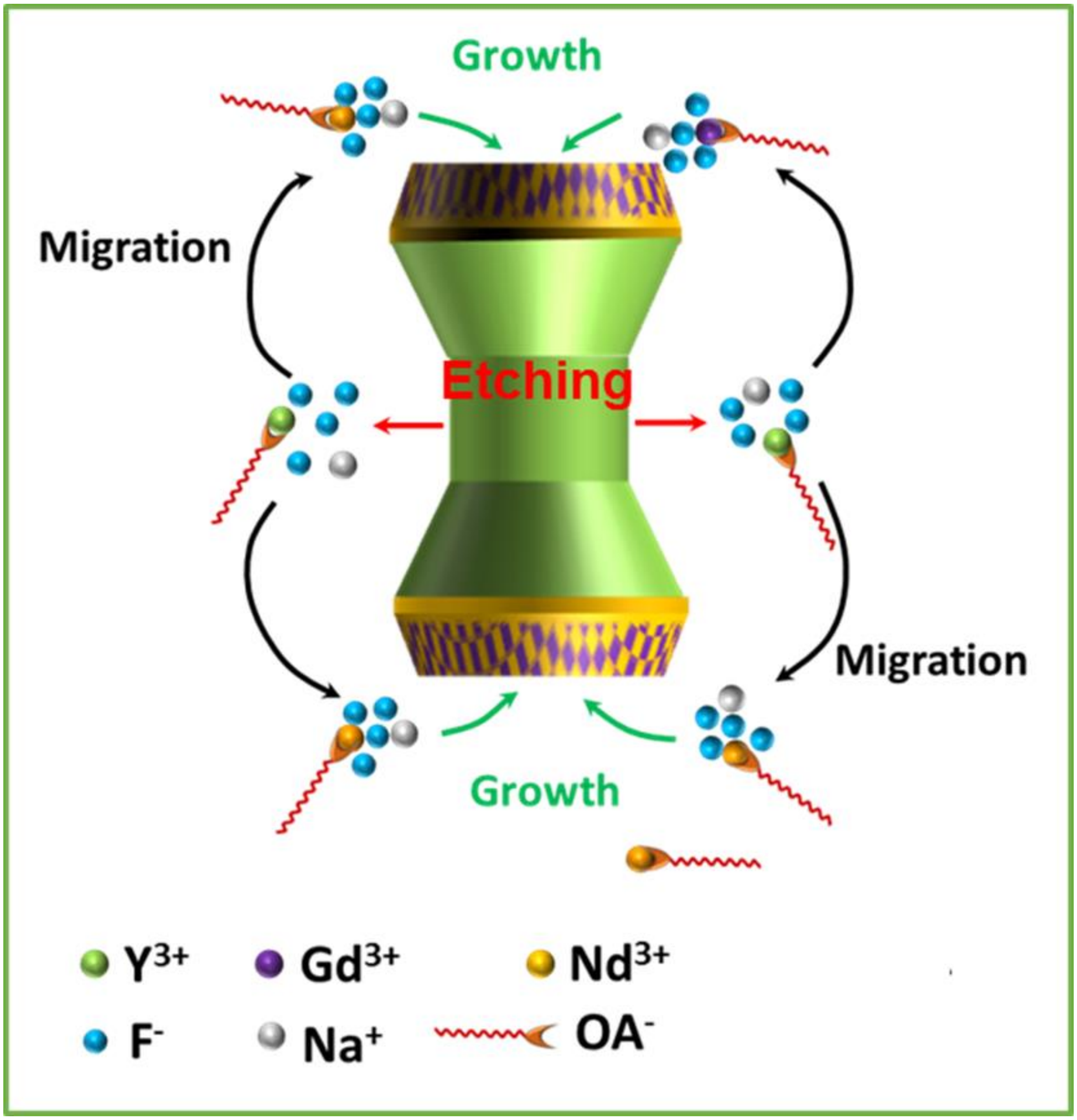

Supplementary Figure 28. Schematic processes of the migration growth. Detailed schematic processes of the simultaneous erosion of the $NaYF_4$-$NaGdF_4$, epitaxial growth of $NaNdF_4$ in the longitudinal direction and the migration growth of F^-, Y^{3+} and Gd^{3+} ions. The etching of $NaGdF_4$-$NaYF_4$ nanocrystals is initially triggered by the OA^- ions strongly bonding to the side surfaces at a high reaction temperature (310 ^oC). As a result, F^-, Na^+ and Y^{3+} ions are released into the solution.

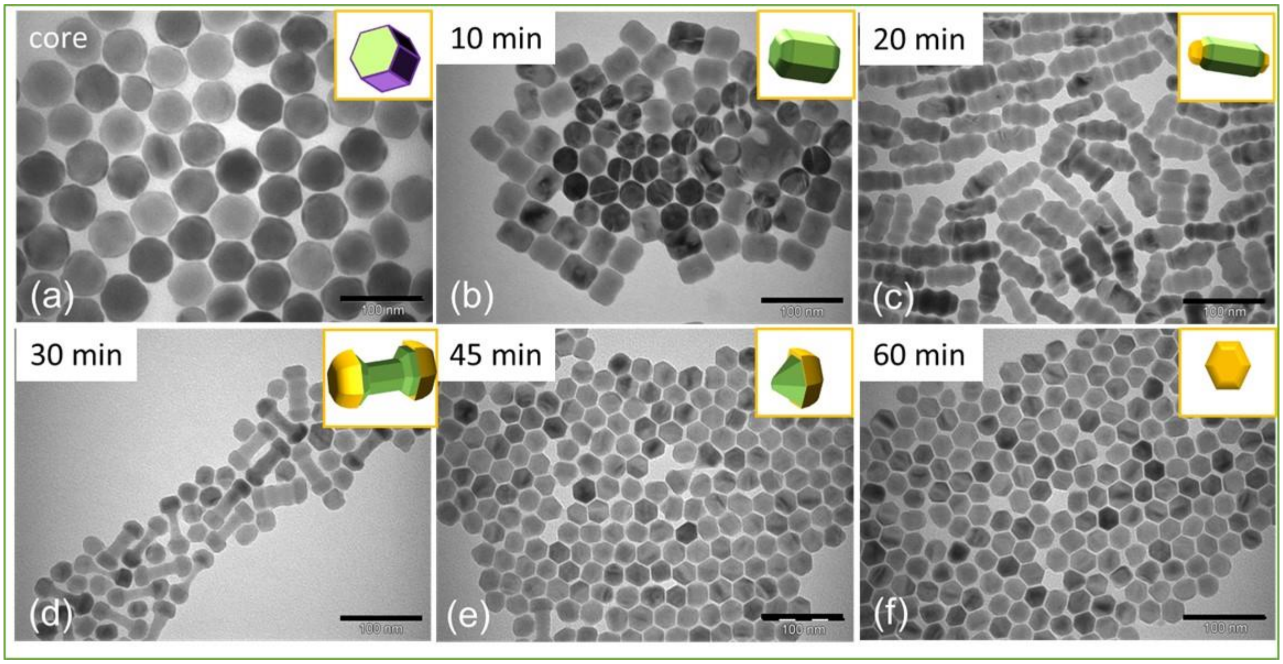

Supplementary Figure 29. Verify the driving force for the selective-surface etching. TEM images of $NaYF_4$/$NaGdF_4$ cores and their migration growth with Nd for 10 min, 20 min, 30 min, 45 min and 60 min after the start of the reaction. (Scale bars: 100 nm)

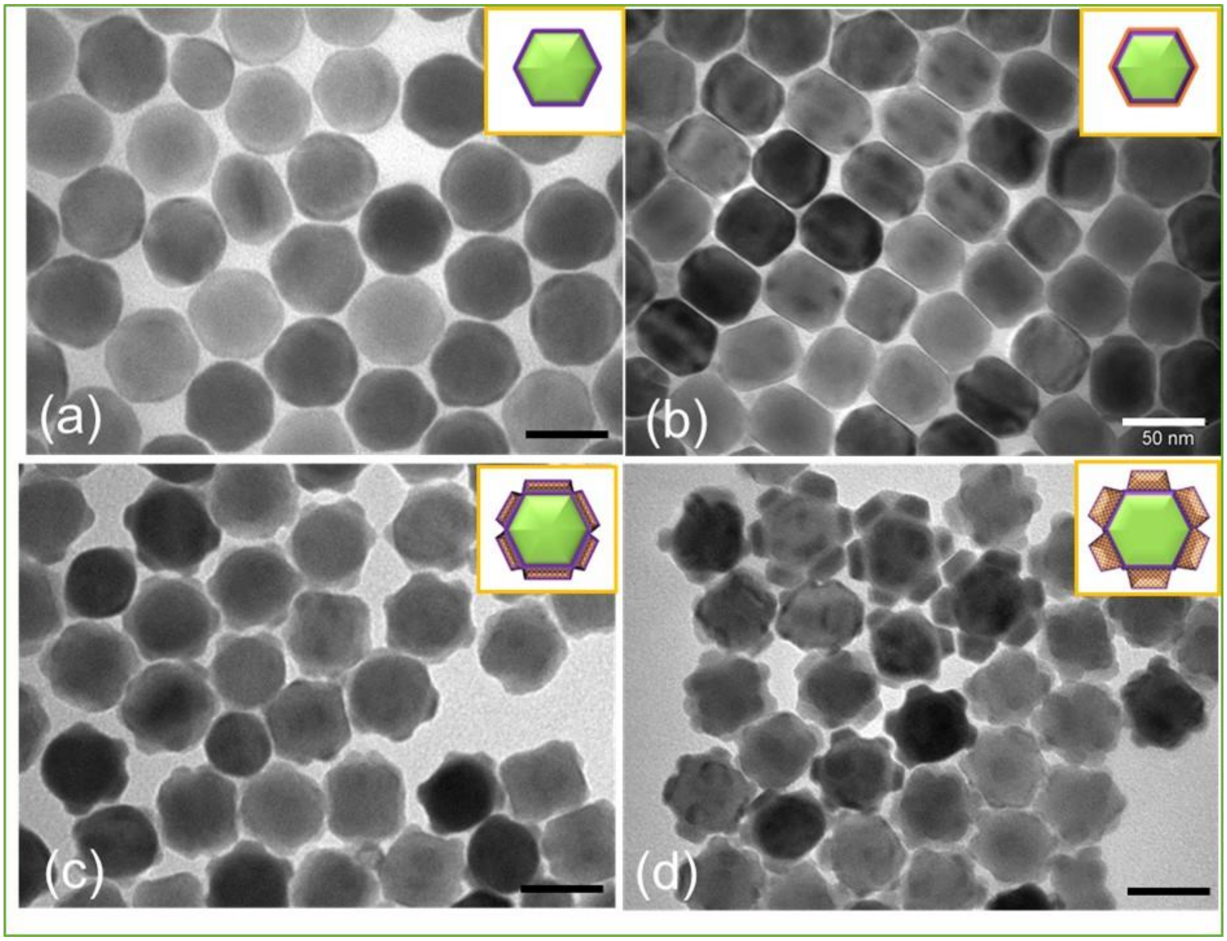

Supplementary Figure 30. $NaYF_4/NaGdF_4/NaNdF_4$ flower-shaped nanocrystals. TEM images of $NaYF_4/NaGdF_4$ nanocrystals as cores, and migration growth with the Nd^{3+} element at 300˚C after 10 minutes, 25 minutes and 45 minutes of reaction time. (Scale bars: 50 nm)

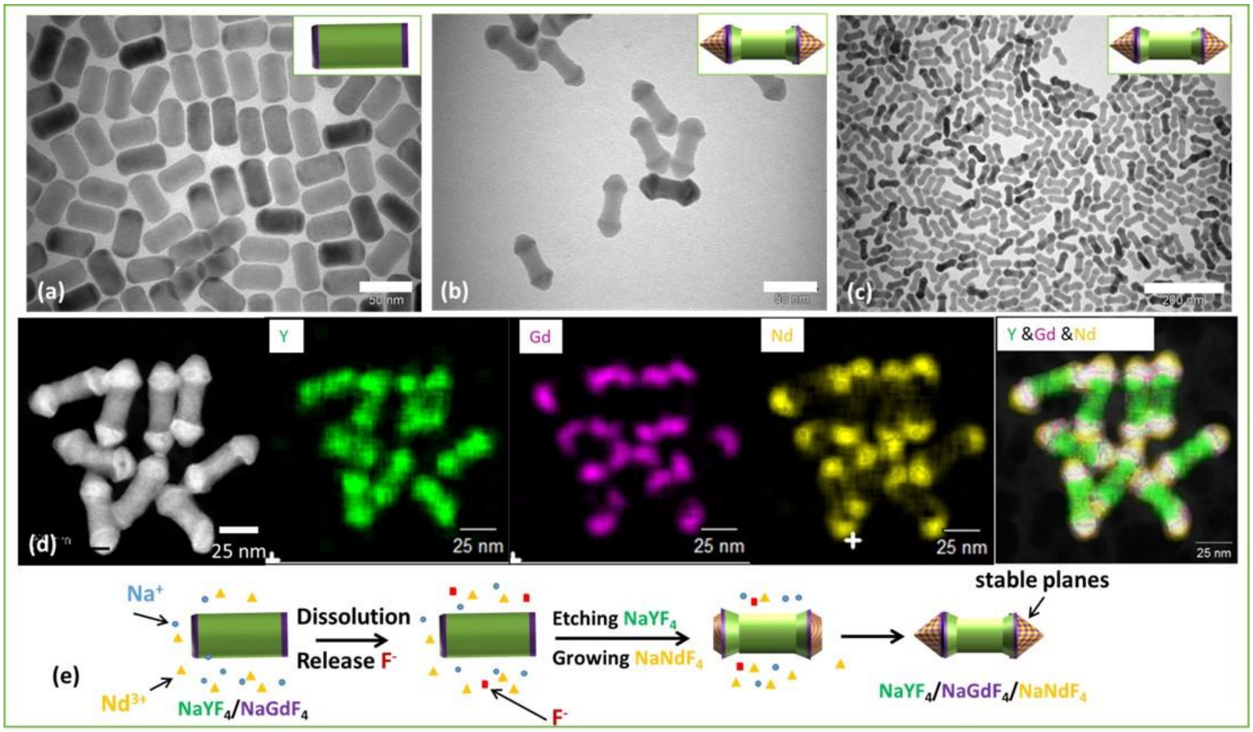

Supplementary Figure 31. $NaYF_4$/$NaGdF_4$/$NaNdF_4$ nanocrystals in sharp-end dumbbell shape. TEM images, elemental composition characterization and schematic illustration of the $NaYF_4$/$NaGdF_4$/$NaNdF_4$ dumbbell-shape nanocrystals with sharp tips via migration growth of $NaNdF_4$ (a) the cores of $NaYF_4$–$NaGdF_4$ nanocrystals with a thin layer of $NaGdF_4$ at the ends of $NaYF_4$ nanorods; (b) the high magnification image and (c) the overview image of the $NaYF_4$/$NaGdF_4$/$NaNdF_4$ dumbbell shape nanocrystals with sharp tips; (d) HAADF-STEM image and elemental mapping images of $NaYF_4$/$NaGdF_4$/$NaNdF_4$ nanocrystals.(e) the schematic illustration of the formation process of $NaYF_4$/$NaGdF_4$/$NaNdF_4$ dumbbell nanocrystals with sharp ends (Scale bar: 50 nm for a and b; 200 nm for c and 25 nm for d)

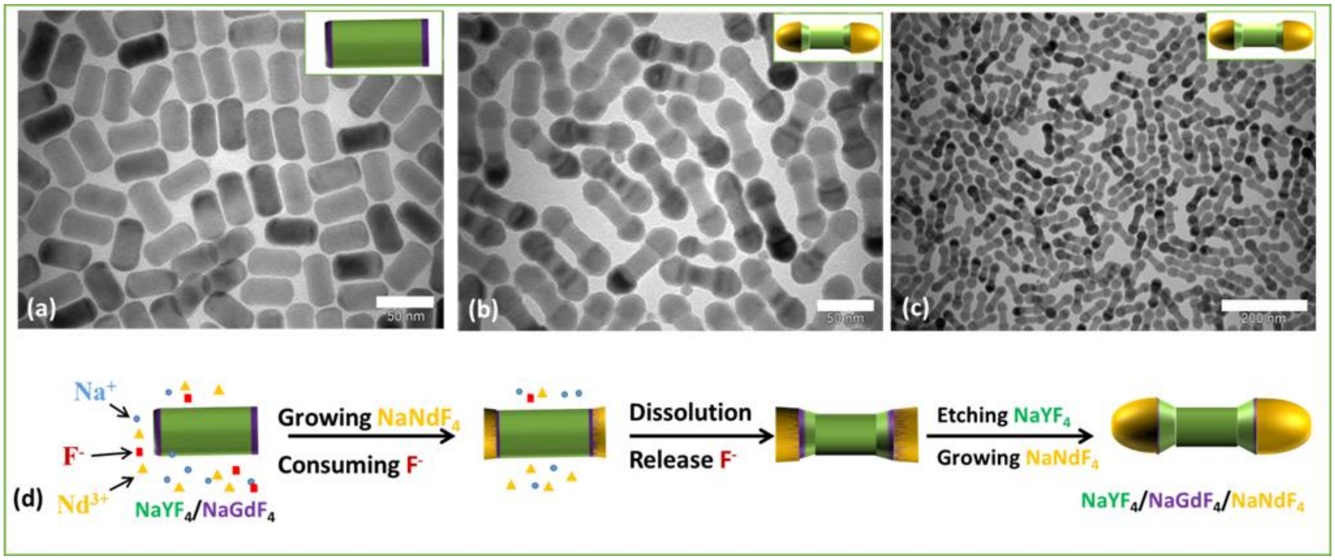

Supplementary Figure 32. $NaYF_4/NaGdF_4/NaNdF_4$ nanocrystals in round-end dumbbell shape. TEM images and schematic illustration of the $NaYF_4/NaGdF_4/NaNdF_4$ dumbbell nanocrystals with round polished tips. (a) $NaYF_4/NaGdF_4$ nanocrystals with a thin layer of $NaGdF_4$ at the ends of $NaYF_4$ nanorods; (b) high magnification image and (c) overview image of $NaYF_4/NaGdF_4/NaNdF_4$ nanocrystals. (d) Schematic illustration of the formation process of the $NaYF_4/NaGdF_4/NaNdF_4$ nanocrystals with round ends. (Scale bar: 50 nm for a and b; 200 nm for c)

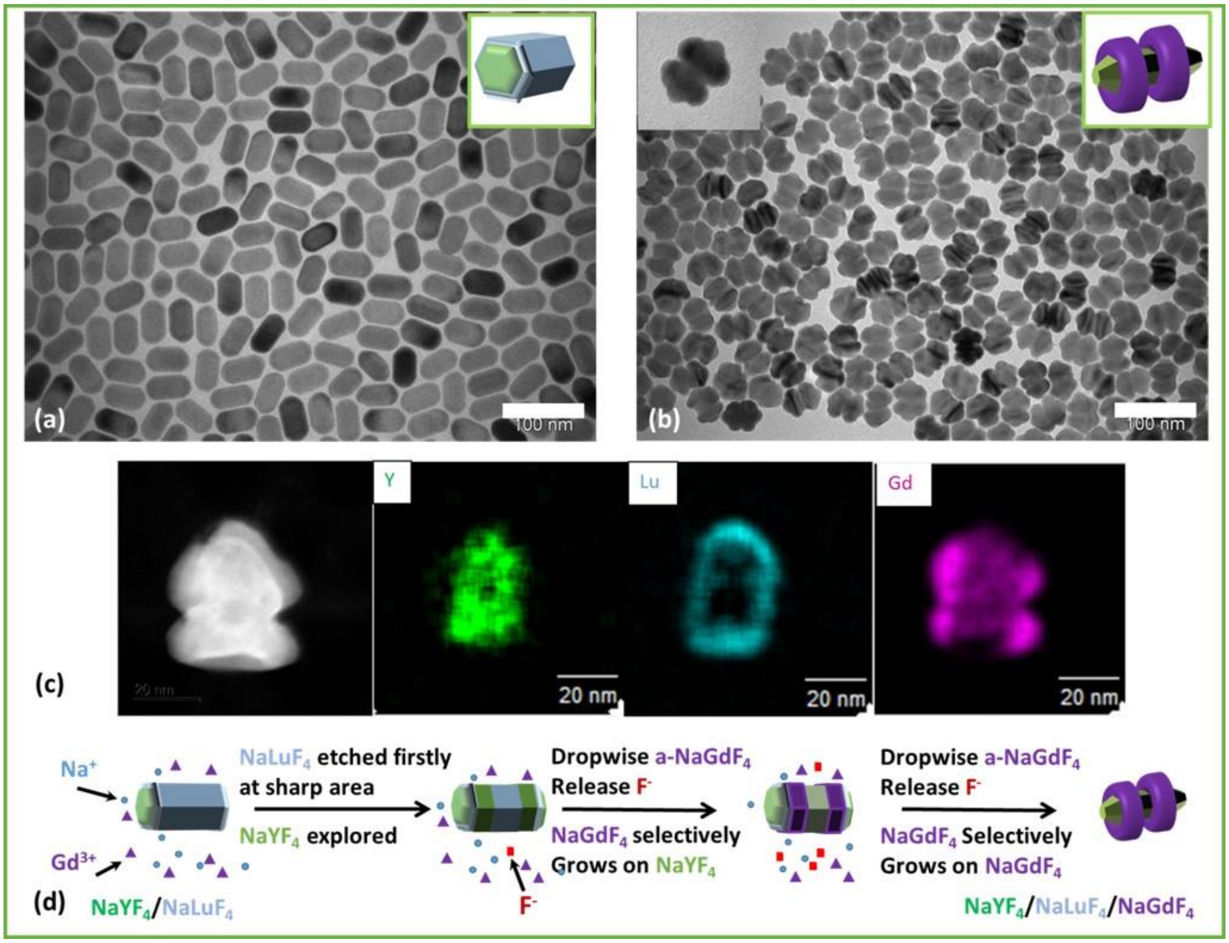

Supplementary Figure 33. **$NaLuF_4$/$NaYF_4$ nanorods with $NaGdF_4$ double-ring structure.** TEM and elemental composition characterization of the $NaYF_4$/$NaLuF_4$/$NaGdF_4$ heterogeneous nanocrystals which form a unique shape of two $NaGdF_4$ rings onto a $NaLuF_4$/$NaYF_4$ nanorod. (a) $NaLuF_4$ nanocrystal shells transversally coated onto $NaYF_4$ core nanocrystals; (b) an overview image of the $NaYF_4$/$NaLuF_4$/$NaGdF_4$ nanocrystals and a single $NaYF_4$/$NaLuF_4$/$NaGdF_4$ nanocrystal (top-left inset); (c) HAADF-STEM image of a single $NaYF_4$/$NaLuF_4$/$NaGdF_4$ nanocrystal and its elemental mapping images. The elemental mapping images confirm the distribution of the Y^{3+} ions in the middle of the rod and the Lu^{3+} ions as a thin layer coating surrounding the nanorod. The Gd^{3+} ions is present as a double ring surrounding the $NaYF_4$-$NaLuF_4$ nanorods in the transversal direction. (d) Schematic illustration of the formation process "selective mask – etching – epitaxial growth" of the $NaYF_4$-$NaLuF_4$-$NaGdF_4$ heterogeneous nanocrystals is provided.

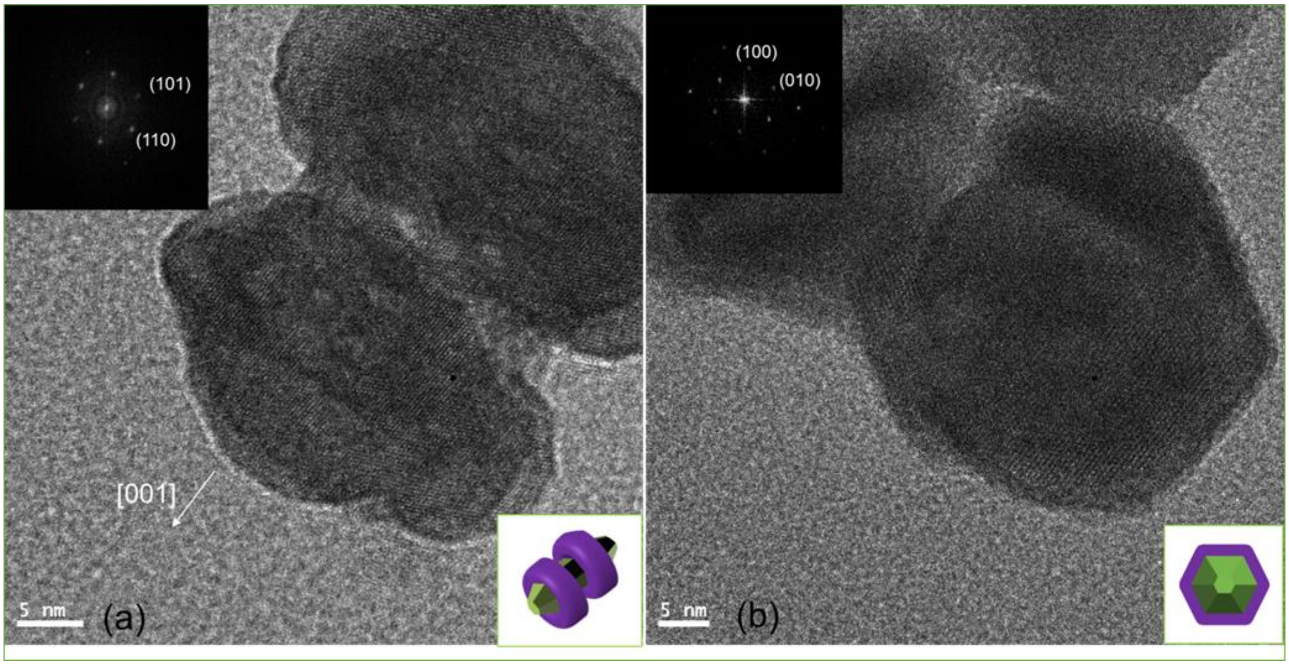

Supplementary Figure 34. High Resolution TEM images of $NaLuF_4$/$NaYF_4$ nanorods with $NaGdF_4$ double-ring structure. (a) A side view high-resolution TEM image and its corresponding selected area electron diffraction pattern (insert) and (b) a top view high-resolution TEM image of a single nanocrystal and its corresponding selected-area electron diffraction pattern (inset)

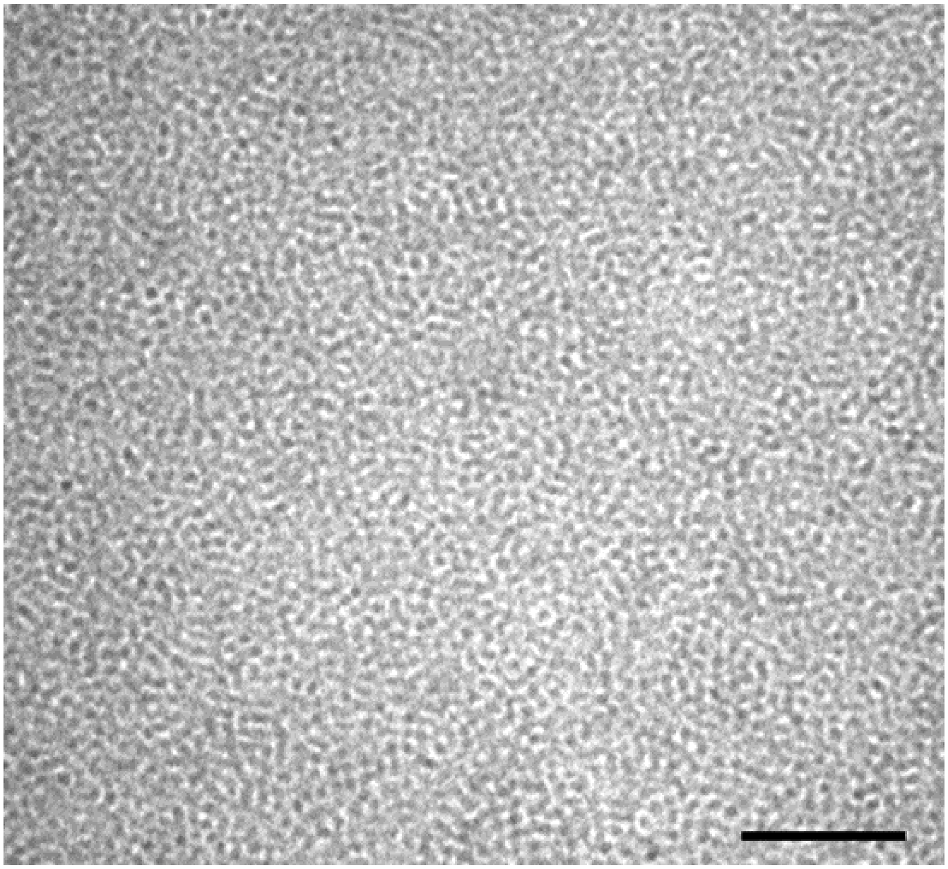

Supplementary Figure 35. α-$NaGdF_4$ nanocrystals. TEM image of α-$NaGdF_4$ nanocrystals used for $NaLuF_4$/$NaYF_4$ nanorods with a $NaGdF_4$ double-ring structure. (Scale bar is 50 nm)

Supplementary Tables

Supplementary Table 1. Binding energy of OA^- and OAH to (001) and (100) planes. The validity of our simplified computational model was confirmed since it displayed the same trend in binding strength observed in the three calculation sets (simplified model, enhanced coverage and real oleic acid model).

	OA^-+001 (eV)	OAH+001 (eV)	OA^-+100(eV)	OAH+100 (eV)
Simplified model	-30.05	-12.23	-37	-6.05
Enhanced coverage	-34.5	-24.5	-73.2	-10.6
Real oleic acid	-21.8	-9.4	-35.4	-4.6

Supplementary Table 2. The crystal lattice parameters of hexagonal phase $NaREF_4$ crystals.

Crytal Host	a & b(Å)	C(Å)
$NaLaF_4$	6.178	3.828
$NaCeF_4$	6.15	3.781
**$NaNdF_4$**	**6.1**	**3.711**
$NaSmF_4$	6.051	3.64
$NaEuF_4$	6.059	3.625
**$NaGdF_4$**	**6.02**	**3.601**
$NaTbF_4$	6.008	3.58
$NaPrF_4$	5.991	3.537
$NaDyF_4$	5.985	3.554
**$NaYF_4$**	**5.96**	**3.53**
$NaErF_4$	5.959	3.514
$NaTmF_4$	5.953	3.494
$NaYbF_4$	5.925	3.471
**$NaLuF_4$**	**5.901**	**3.453**

Supplementary Table 3. Summary of the lattice mismatch for crystallographic parameters *c* for a hexagonal phase $NaREF_4$ crystals. The crystallographic potential for high quality epitaxial growth increases with a change of color from green to red.

	$NaLuF_4$	$NaYbF_4$	$NaTmF_4$	$NaErF_4$	**$NaYF_4$**	$NaDyF_4$	$NaPrF_4$	$NaTbF_4$	**$NaGdF_4$**	$NaEuF_4$	$NaSmF_4$	**$NaNdF_4$**	$NaCeF_4$	$NaLaF_4$
$NaLaF_4$	0.109	0.103	0.096	0.089	0.084	0.077	0.082	0.069	0.063	0.056	0.052	0.032	0.012	0.000
$NaCeF_4$	0.095	0.089	0.082	0.076	0.071	0.064	0.069	0.056	0.050	0.043	0.039	0.019	0.000	
**$NaNdF_4$**	0.075	0.069	0.062	0.056	**0.051**	0.044	0.049	0.037	**0.031**	0.024	0.020	0.000		
$NaSmF_4$	0.054	0.049	0.042	0.036	0.031	0.024	0.029	0.017	0.011	0.004	0.000			
$NaEuF_4$	0.050	0.044	0.037	0.032	0.027	0.020	0.025	0.013	0.007	0.000				
**$NaGdF_4$**	0.043	0.037	0.031	0.025	**0.020**	0.013	0.018	0.006	0.000					
$NaTbF_4$	0.037	0.031	0.025	0.019	0.014	0.007	0.012	0.000						
$NaPrF_4$	0.024	0.019	0.012	0.007	0.002	0.005	0.000							
$NaDyF_4$	0.029	0.024	0.017	0.011	0.007	0.000								
**$NaYF_4$**	0.022	0.017	0.010	0.005	0.000									
$NaErF_4$	0.018	0.012	0.006	0.000										
$NaTmF_4$	0.012	0.007	0.000											
$NaYbF_4$	0.005	0.000												
$NaLuF_4$	0.000													

0-0.03   0.031-0.05   0.051-0.11

Supplementary Notes

Supplementary Note 1 – Surface and absorption models

In order to unravel the underlying mechanism of ligand directed growth of nanocrystals, the density functional theory (DFT) based first principles calculations were performed using CASTP[1] in Material Studio. We simulated the interactions between the surface of β-$NaYF_4$ and selected ligands. Throughout the entire DFT calculations we have implemented a generalized gradient approximation (GGA) using a kinetic energy cut-off of 500 eV for the plane wave. The calculated lattice constants used for the bulk β-$NaYF_4$ were a = 6.001 Å and c = 3.603 Å, which were similar to the experimental values observed (a = 5.96 Å, c = 3.53 Å for β-$NaYF_4$).[2] The surface structure was modeled using a super-cell which consisted of a 10-layer slab separated by a vacuum of 15 Å. In the case of un-passivated surfaces, a more general expression of surface energy, applicable to both stoichiometry and non-stoichiometry surface models, was used:

$$\sigma = \frac{1}{2A}[E_{tot}(Slab) - n_{Na}\mu_{Na} - n_Y\mu_Y - n_F\mu_F][1]$$

Where $E_{tot}(Slab)$ is the total energy of the slab, n_{Na}, n_Y and n_F are the number of Na, Y and F atoms in the slab, and μ_{Na}, μ_Y and μ_F are their corresponding chemical potentials. The (001) and (100) facets of β-$NaYF_4$ with differently terminated atomic configurations were also investigated, and the most stable (001) and (100) surfaces calculated, with the surface energies of 44.58 meV $Å^{-2}$ and 57.43 meV $Å^{-2}$, are shown in **Supplementary Fig. 1**. The surface relaxation was also considered inside the top two layers which are free to relax without constraint, and our simulations were unable to show a significant change in the location of the atoms after relaxation. We calculated the capping of (001) and (100) facets by OAH and OA^- ligands presented in the solution using the adsorption model of ligands onto these two stable surfaces. The spurious interaction between ligands caused by the periodic boundary condition in our case can be ignored since the distance between the ligands is about 10 angstrom, which is a sufficient distance for the quantum simulation. In particular, the long carbon chain is a sufficient distance from the active functional group of OAH and OA^- ligands to make the surface binding energy negligible. Hence, we used two simplified molecules, namely $C_2H_3O_2$ and $C_2H_4O_2$ to mimic the adsorption characters of the OA^- and OAH ligands to the β-$NaYF_4$ facets, respectively. Note that in our simulation, atoms in the top two layers were allowed to relax until the force on each atom was less than 0.02 eV/Å while the other atoms were fixed (shadow areas shown in **Supplementary Fig. 2** and **3**). Since the oxygen atoms of the ligands prefer to bind with Y^{3+} ions in cubic $NaYF_4$, [3] we only considered the interaction of molecule with Y^{3+} ions when testing different initial configurations. The initial adsorption configurations of ligands onto the

(001) and (100) surfaces were considered, and are illustrated in **Supplementary Fig. 2** and **3**, respectively. The binding energy was determined by

$$E_b = E(Combined) - [E(Surf) + E(Mol)] [2]$$

Where $E(Combined)$, $E(Surf)$ and $E(Mol)$ were the energies of the binding complex, the $NaYF_4$ slab and the isolated molecule, respectively. The binding energies of these molecules (first row in Table S1) follow the sequence of $E_b(OA^-) > E_b(OAH)$ both for (001) and (100) surfaces as a result of the chemical activity of the molecules. Furthermore, we also found that the adsorption of OA^- ligands onto (100) facet was stronger than that of (001), whereas the OAH ligands prefer to be adsorbed onto the (001) compared to (100). The different binding strength can be ascribed to the different atomic arrangement of Y^{3+} ions on these two facets as seen in **Supplementary Fig. 1(c)**, which give rise to different charge transfer paths between ligands and surfaces (**Supplementary Fig. 6**).

Supplementary Note 2 – Effect of coverage and steric carbon chain of ligand on binding energy

The aforementioned proposed (001) and (100) adsorption models correspond to the ligand surface coverage of 0.8 and 0.58 ligand nm^{-2}, respectively. In order to clarify the effect of the coverage on the binding energies, we minimized the slab models, giving rise to enhanced coverage of 1.6 and 1.15 ligand nm^{-2} for (001) and (100) facets, respectively. Here the adsorption model of OA^- on (001) with low and high coverage is shown in **Supplementary Fig. 4**. According to the calculated binding energies (row two in **Supplementary Table 1**), we found that the increased coverage that was applied in this example did not affect the binding trend of the original calculation, which was based on a low ligand coverage.

To mimic the experimental situation, we also calculated the interaction between the slabs and the real oleic acid molecules containing 18 carbon atoms (**Supplementary Fig. 5**). The corresponding binding energies (the last row of **Supplementary Table 1**) indicate that the binding trend of the original calculation based on a simplified molecule model is still accurate when the real oleic acid molecules are taken into consideration. It should be noted that a decrease of the absolute values of the binding energy was observed, indicating that the long alkyl tail can weaken the interaction between molecules and the surfaces that were tested.

In summary, the validity of our simplified computational model was confirmed since it displayed the same trend in binding strength observed in the three calculation sets (simplified model, enhanced coverage and real oleic acid model). Since the binding energy of OA^- is much higher than that of OAH in both of (001) and (100) planes, OA^- has a lot stronger passivating effect at the crystal facets. For example, the binding energy of OA^- at (100) planes is about 13.6 eV higher than its binding energy at the (001) planes. This suggests that OA^- has a stronger bond at (100) planes (lateral faces of hexagonal nanocrystals) than at the (001) planes (top and bottom faces of hexagonal nanocrystals) In contrast, OAH has a higher binding energy at the (001) planes than that at the (100) planes, which suggests that OAH has a stronger bond at the (001) planes (top and bottom faces) than that at the (100) planes (lateral faces). Therefore, in the condition where there is a high ratio of OA^-/OAH, OA^- will become the major surfactant that passivates the (100) planes (lateral faces) and promotes crystal growth along the [001] crystallographic direction. On the other hand, where there is a low ratio of OA^-/OAH, OAH will become the major surfactant, which makes the top and bottom faces relatively more passive in comparison to the lateral faces. Therefore the growth direction of nanocrystals can be controlled from longitudinal to transverse by adjusting the ratio of OA^- to OAH.

Supplementary Note 3 – The reaction temperature effect to epitaxial shell growth

As shown in the **Supplementary Fig. 12**, within the wide range of the reaction temperature (290 to 320 ˚C), the epitaxial growth occurred in the longitudinal direction at the high ratio of OA^-/OAH. We noted that the synthesis was accompanied with the newly formed nanocrystals at 290 ˚C, which suggested the shell growth speed at 290 ˚C was too slow to complete the shell growth within 1 hour. At an elevated temperature 310 ˚C, the shell growth speed was increased and longer nanorods were formed. With this obvious trend in the longitudinal growth direction, however, the higher temperature of 320 ˚C unexpectedly resulted in aggregated nanorods. These results indicated that the reaction temperature will only influence the speed of the directional epitaxial growth, but it requires an optimum temperature (310 ˚C) to produce the crystal with a high degree of mono dispersity.

By investigating the temperature effect on the growth direction, these results provide additional evidence to support the mechanism uncovered in this work that is ruled by a binding energy difference between OA^- and OAH onto the (001) and (100) planes. At a low temperature there is not sufficient thermal vibration, so both of the surfaces are well passivated by OA^- and OAH surfactants which results in a slow speed of growth, although it should be noted, there is relatively better passivation to the (100) planes with a greater availability of OA^- surfactants due to the high ratio of OA^-/OAH. Increasing the temperature to 300 ˚C and 310 ˚C increases the thermal agitation that unlocks (or unbinds) the OAH off the (001) plane which then promotes longitudinal growth.

The interesting result of aggregated nanorods shown in **Supplementary Fig. 12 (e)** further suggests that the higher-temperature induced thermal agitation at 320 ˚C may even affect the stability of strong binding of the OA^- to the (100) planes which then leads to the observed aggregation.

Brief conclusion:

The reaction temperature does not alter the direction of epitaxial growth, but does affect the growth speed. At lower reaction temperature (290 ^{o}C), both OA^- and OAH surfactants are stable and passivate the crystal surfaces, which then slows down the epitaxial growth speed; at a higher temperature (300 and 310 ^{o}C), the OAH surfactants become unstable on the (001) planes and the end surfaces become unlocked which then allows the obvious longitudinal shell growth; at a higher temperature (320 ^{o}C), both surfactants become unstable which causes aggregation and a nanorods at a low stability.

Supplementary Note 4 – The OA concentration effect on epitaxial shell growth

As shown in the **Supplementary Fig. 13**, with a decrease in the OA concentration from 1.70 mmol/mL to 0.85 mmol/mL, the epitaxial shell growth direction changed from homogenous to longitudinal. This result provides further support for our proposed mechanism that the ratio of OA^-/OAH determines the epitaxial shell growth direction. The high concentration of OA which equals the high concentration of OAH, can well passivate the (001) planes and subsequently decreases the OA concentration, which itself equivalently increases the ratio of OA^-/OAH. This then leads to a reduction in the passivation effect on the ends surfaces ((001) planes) and encourages epitaxial shell growth along the longitudinal growth direction.

Brief conclusion:

The OAH molecule concentration primarily determines the growth activity on the (001) plane, the OA^- passivates the (100) planes, and the balance between OA^- and OAH (the ratio of OA^-/OAH), determines the epitaxial shell growth direction.

Supplementary note 5 – The role of F^- concentration to shell growth.

As shown in **Supplementary Fig. 14**, by increasing the concentration of F^- from 36.4 μmol/mL to 54.5 μmol/mL, and to 72.8 μmol/mL, the nanocrystals grew only along the longitudinal direction, but with an obvious trend that higher concentration of F^- leads to longer nanocrystals.

The observation of epitaxial shell growth only along the longitudinal direction is consistent with the longitudinal growth condition at fixed ratio of OA^-/OAH; Varying the F^- concentration only affects the speed of epitaxial shell directional growth, which is expected since the higher concentration of F^- ions supplies a sufficient amount of ions and participate faster growth.

Using very low concentration of F^- ions enhanced the crystal dissolution effect and therefore produced a range of uniquely shaped nanocrystals, which was thoroughly investigated and presented in controlled migration growth section.

Brief conclusion:

The F^- concentration had little effect on the epitaxial shell growth direction, although higher concentration of F^- resulted in longer nanocrystal rods.

Supplementary Note 6 – The role of Na^+ concentration to shell growth.

As shown in the **Supplementary Fig. 15**, after decreasing the Na^+ concentration from 45.5 μmol/mL to 27.3 μmol/mL, the shape of core-shell nanocrystals changed from nanorods to nanospheres.

The obvious changes in the shape of core-shell nanocrystals suggest that the epitaxial shell growth direction was highly related to the Na^+ concentration. The increase of the Na^+ concentration causes more OAH to react with OA^-, which leads to an increase in the ratio of the OA^-/OAH. A higher ratio of OA^-/OAH leads to longitudinal shell growth under 310 °C. This result further supports our mechanism that the ratio of OA^-/OAH is a key factor that controls the epitaxial shell growth direction.

Brief conclusion:

Variation to the Na^+ concentration changes the epitaxial shell growth direction due to the fact that the ratio of OA^-/OAH is altered. A higher Na^+ concentration (45.5 μmol/mL) leads to the increase of the ratio of OA^-/OAH that results in longitudinal shell growth.

Supplementary Note 7 – Ruling out OH^- as a key factor for longitudinal growth

In order to rule out the interference from OH^- (from NaOH) that may play a role in directional epitaxial growth and to further verify that the oleate ion (OA^-) plays a major role in passivating the side surfaces (100), we carried out an experiment where we used sodium oleate (NaOA) to replace NaOH as the sodium source. As a comparison, in one control experiment we grow the $NaYF_4$ nanocrystals with $NaYF_4$ shell using NaOH as sodium source, and in the second experiment, we used the same molar amount of Na-OA (sodium oleate) as a sodium source and as a replacement for the NaOH.

As shown in **Supplementary Fig. 16 (b) and (c)**, identical results were obtained with $NaYF_4$ shell grown longitudinally onto the $NaYF_4$ core no matter if OH^- was present in the reaction or not, ruling out OH^- as a key factor for longitudinal growth in the reaction system described here.

Brief conclusion:

The ratio of OA^- to OAH was a key factor in directing longitudinal growth of the crystals while the amount of NaOH is indirectly related to the ratio of OA^- to OAH.

Supplementary Note 8 – Longitudinal growth of heterogeneous $NaYF_4$/$NaGdF_4$ core/shell

The process of longitudinal growth of $NaGdF_4$ shell onto the $NaYF_4$ core shown in **Supplementary Fig. 17** is similar to that seen in the$NaYF_4$/$NaYF_4$ nanorods structure (**Supplementary Fig. 8)**. Small α phase $NaGdF_4$ nanocrystals were formed at the beginning of the reaction step. After 15 minutes the shape of $NaYF_4$ cores changed from spherical to hexagonal. After 30 minutes, thin layers of $NaGdF_4$ have formed on the bottom and top surfaces (001) of the $NaYF_4$ hexagonal prism and the $NaGdF_4$ layer has also formed along the c axis. At the completion of the reaction, $NaGdF_4$/$NaYF_4$ dumbbell-shape nanocrystals have formed which we attributed to the selective growth of the $NaGdF_4$ shells along the longitudinal direction. The concentration of shell source reagents in the reaction mix decreased rapidly as it was consumed by the growth of the β-$NaGdF_4$ shell and the α-$NaGdF_4$ nanocrystals. As a result of this decrease, α-$NaGdF_4$ nanocrystals were rapidly dissolved and formed the β-$NaGdF_4$ shell.

The results of the characterization (**Supplementary Fig. 18**) of another validation experiments showed that the heterogeneous nanocrystals had a dumbbell shape with $NaGdF_4$ nanocrystals at the two ends. The dissolution (etching) phenomenon from the side surfaces (100) of $NaYF_4$ core was observed, which was attributed to the strong binding of the oleate ions (OA^-) to the exposed rare earth ions on the side surfaces of the particles. Detailed investigations of this interesting phenomenon were carried out and are described in part 3 of this supplementary material. The length of the $NaYF_4$/$NaGdF_4$ nanocrystals was observed to visibly increase in the longitudinal direction from 42 nm to 62 nm, while the width of $NaYF_4$ in the middle of each nanorods decreased by 8 nm, due to the dissolution of the $NaYF_4$ crystal during growth of the $NaGdF_4$. The width of the newly formed $NaGdF_4$ crystals at each end was 29 nm, which was similar to the original width of the $NaYF_4$ core nanocrystals.

The HAADF-STEM images (**Supplementary Fig. 18 (c)**) demonstrate the density contrast of the $NaGdF_4$ at the ends and the $NaYF_4$ in the middle and the elemental mapping images confirm the distribution of the elements Y and Gd. The combined elemental mapping image confirms that the two compositions are well aligned.

Brief conclusion:

At a high concentration of oleate ions (OA^-), the deposition and epitaxial growth of heterogeneous shells ($NaGdF_4$) still prefer the bottom and top surfaces (001) of $NaYF_4$ crystal core.

Supplementary Note 9 – Accelerated longitudinal growth by adding KOH

KOH has a higher dissociation constant than NaOH. We thus postulated that adding additional KOH increases the amount of OA^- dissociated from OAH and increases the passivation effect on the side surfaces of the particles. This is expected to accelerate the epitaxial growth in longitudinal direction. To verify this hypothesis, we added 0.4 mmol of KOH to the reaction mix.

With the aid of KOH, the longitudinal growth of the $NaYF_4$ and $NaGdF_4$ nanocrystals became faster and new formed nanocrystals on the two ends of cores were longer in contrast to the particles formed when KOH was not present in the mix. The final nanorods (**Supplementary Fig. 19**) or nanocrystal dumbbells (**Supplementary Fig. 20**) have sharper edges compared to the ones produced without the presence of KOH. This suggests that the amount of the OA^- ions available on the side surfaces during the reaction to passivate the side surfaces (100) by acting as surfactants was sufficient.

Brief conclusion:

KOH can rapidly supply sufficient OA^- ions on the side surfaces and therefore it promotes the longitudinal growth of the nanocrystals. KOH reacts with Oleic Acid (OA) and forms KOA, which significantly increasing the ratio of OA^- to OAH. The higher ratio of OA^-/OAH promotes longitudinal growth.

Supplementary Note 10 – Multiple-section $NaYF_4$/$NaGdF_4$ Nanorods

This experiment further verified the mechanism of the longitudinal shell growth. The core width is determined by the equilibrium of shell growth rate and core dissolution rate on lateral faces. Due to strong passivation from OA^- ions onto the lateral faces, the shell growth rate on lateral faces is a lot of slower compared with the growth rate on the two ends. The core dissolution rate is determined by the thermal stability difference between core and shell: If the shell materials have a higher thermal stability than the core material, the crystal dissolution at the core will occur resulting in the decrease of core width, as shown in **Supplementary Fig. 21**; in contrast, if the shell materials have a lower thermal stability than the core material, the core crystal dissolution will stop and shell growth will dominate on lateral faces.

The synthesis approaches in **Supplementary Fig. 21**and **Supplementary Fig. 22** are the same, in fact the structure showed in **Supplementary Fig. 21** ($NaGdF_4$/$NaYF_4$/$NaGdF_4$) was used as the precursor core for the five section bamboo (**Supplementary Fig. 22(a)**), which then was used as the core for the sequential growth of the seven section bamboo structure (**Supplementary Fig. 22(b)**). The High-angle annular dark-field (HAADF) scanning transmission electron microscope (STEM) image (**Supplementary Fig. 22(c)**) clearly recorded this process.

When the ($NaGdF_4$ - $NaYF_4$- $NaGdF_4$) structure was formed, as shown in **Supplementary Fig. 21**, the $NaYF_4$ core was dissolved but with a thin layer of $NaGdF_4$ shell grown on the lateral faces (see **Supplementary Fig. 22(c)** HAADF-STEM image); when the five section ($NaYF_4$- $NaGdF_4$ - $NaYF_4$- $NaGdF_4$- $NaYF_4$) structure was formed, the lateral faces were terminated and protected by the $NaGdF_4$ crystal surfaces, so that dissolution stop with additional $NaYF_4$ grown to increase the core width, which is due to the fact that $NaGdF_4$ is more stable than $NaYF_4$; when the seven section ($NaGdF_4$ - $NaYF_4$- $NaGdF_4$ - $NaYF_4$- $NaGdF_4$- $NaYF_4$- $NaGdF_4$) structure was formed, shown **Supplementary Fig. 22(b) (c)**, clearly the $NaGdF_4$ was epitaxial grown onto the lateral faces as well.

Brief conclusion:

It is well known that the co-precipitation method is useful for the synthesis of sub-100 nm nanocrystals, while the hydrothermal method is suitable for synthesizing larger sized nanocrystals in the micron size range.[9,10] Our current experiments show that with the aid of high concentration of NaOH or KOH in the reaction $OA^{-,}$ ions can passivate effectively the side surfaces (100) (010) of nanorods and so grow very long homogeneous or heterogamous nanocrystals with some control.

Supplementary Note 11 – **Transversal growth of heterogeneous $NaYF_4/NaGdF_4$ core/shell**

To develop a set of approaches for the programmable growth of arbitrary shapes of nanocrystals, and according to the selective preference of OAH molecules and OA^- ions to different facets of nanocrystal cores, we identified the condition of the lower ratio of OA^-/OAH at slightly lower temperature (290 ^{o}C vs. 310 ^{o} C) which better passivate the top and bottom surfaces (001) of the nanocrystal cores so that the epitaxial shell growth will be based on the side surfaces (100) along their transversal direction.

TEM characterization results shown in **Supplementary Fig. 23** and **Supplementary Fig. 24** confirm a core-shell structure when viewed from the top of the nanocrystals, while the insert image (**Supplementary Fig. 23 (b)**) and high resolution image of (**Supplementary Fig. 23 (d)**) reveal a core-ring structure when viewed from the side, with the shell grown only around the side surfaces of the nanocrystal cores.

The length of the nanocrystal slightly reduces from 41 nm to 39 nm after transversal growth, while the size in diameter increases from 50 nm to 65 nm, indicating that the thickness of $NaGdF_4$ ring layer was 7.5 nm. The height of $NaGdF_4$ ring was measured and found to be 18.2 nm.

It is worth noting that the diameter of the $NaYF_4$ core nanocrystals at its two ends was reduced from 49 nm to 30 nm, suggesting a disillusion (etching) phenomenon occurred on the end edges during the epitaxial growth of $NaGdF_4$ rings in the middle part of the core. The elemental mapping images in **Supplementary Fig. 23 (c)** and **(d)** demonstrate that the ion Gd^{3+} was deposited within the rings with the ion Y^{3+} deposited in the core.

From the high resolution TEM images in **Supplementary Fig. 24**, we can confirm that the growth direction of $NaGdF_4$ shell was vertical to the [001] direction. The (110) plane distance at the top edge area was 2.97 Å while the (110) distance at the middle area was 3.02 Å, which is consistent with the standard parameters of the β-$NaYF_4$ from pdf #16-0334 and the β-$NaGdF_4$ from pdf #27-0699.

Brief conclusion:

According to the computational modeling results performed in this work, the binding energy of OAH on the (001) facet was two times stronger than that calculated on the (100) facets, and the relatively higher concentration of OAH molecules generated a stronger passivation effect to block the access for epitaxial growth of shells onto the end surfaces. Under relatively low reaction temperature that facilitates the binding of OAH molecules, the difference in binding strength of the surfactants covering on each

facet caused the anisotropic shell formation. Even though the difference was smaller than that observed in the longitudinal growth, the result was still very clear when a shorter reaction was performed.

With a lower ratio of OA^{-}/OAH (which equates to a relatively low OA^{-} concentration) and at lower reaction temperature, the OAH molecules were the main surface ligands preferably on the top and bottom surfaces of nanocrystal cores, inhibiting the end surfaces (001) and therefore promoting the epitaxial growth of $NaGdF_4$ shell in its transversal direction.

The etching phenomenon observed on the ends of the side surfaces (100) of the $NaYF_4$ was due to the strong binding of the oleate ions (OA^{-}) to the exposed rare earth ions on the side surfaces of the particles.

Therefore, for the longitudinal growth, 310 ˚C was selected. On the other hand, with a lower ratio of OA^{-} and OAH, OAH will become the major surfactant, which attach itself to the (001) plane with a higher bonding energy than that the (100) planes, but a much lower bonding energy then the OA^{-} attached to the (100) planes at higher ratio of OA- and OAH. To keep the binding of OAH to the (001), 290 ˚C was used for encourage transversal growth.

Supplementary Note 12 – The effect of shell's stability on crystal dissolution.

As shown in the **Supplementary Fig.25**, comparing to $NaGdF_4$ as shell (**Supplementary Fig. 25 (b)**), only slight dissolution was observed during the growth of $NaTbF_4$ as shell (**Supplementary Fig. 25 (c)**), but no dissolution effect was observed when using the $NaYbF_4$ as the shell (**Supplementary Fig. 25 (d)**), in which case, rather than being dissolved from the core, the overall size of the nanorods was increased.

These results suggest that the dissolution of core is intrinsically caused by the thermal stability between core and shell materials, for example, $NaGdF_4$ shell is more stable than $NaYF_4$ core [2] so that the core was dissolved. The degree of thermal stability of the different $NaLnF_4$ nanocrystals affects the dissolution rate. Comparing the dissolution rates using $NaTbF_4$ as shell and $NaGdF_4$, the dissolution in the case of $NaGdF_4$ was more obvious. But because the $NaYbF_4$ is less stable than the core $NaYF_4$, the core was stable without any observed dissolution. A similar result was observed when growing $NaYF_4$ onto $NaGdF_4$; there was no dissolution, but a slightly increase in its diameter (see (**Supplementary Fig. 21**).

Although it has been confirmed in **Supplementary Fig. 14** that the concentration of F^- did not affect the direction of the epitaxial growth (**Supplementary Fig. 15**) as long as the OA^-/OAH ratio remained unchanged, the concentration of F^- can be used to kinetically control the degree of dissolution in the core because of the gradient in the reagent concentrations used. The dissolution only occurred slowly at high concentration of F^- source (usually 72 μmol/ml; see **Supplementary Fig. 17-27**), but it became much more apparent and was completed sooner when the epitaxial growth was conducted without F^- source (see the main text Figure 3, the hourglass shaped nanocrystals). When the concentration of F^- source was adjusted to a medium level, the dissolution rate and amount from the core nanocrystals was accordingly controlled.

Brief conclusion:

The dissolution of core was caused by a thermal stability between core and shell materials. Dissolution required that the shell materials have a higher thermal stability than the core material to ensure the crystal dissolution at the core. The larger difference in thermal stability between the core and its shell will result in a higher dissolution rate.

Supplementary Note 13 – Crystal lattice mismatch of β-$NaREF_4$ family crystals.

Rare earth doped nanocrystals have emerged as a means to provide exceptional optical, magnetic and physical-chemical properties, for photon upconversion, background-free biological assays, multimodal *in vivo* bio-imaging (fluorescence, MRI, X-ray, SPECT, etc.), targeted drug delivery as carriers, cancer therapy, full color displays, infrared upconversion photovoltaic and photo catalysis for energy management, security inks, and photonics. As a logically extensive of its these applications, integrating multiple functions in single nanoparticles can provide more cross-linking options and new possibilities in as yet unrealized frontier areas. Multi-functionalizing heterogeneous nanoparticles present an extremely attractive research topic that is motivated by both physical-chemistry researches and biomedical life science applications. To realize the myriad of these opportunities, readily scalable, hybrid materials should be synthesized through an integrated approach so that its different structural constituents can collectively contribute to the same challenge.

To facilitate scalable synthesis protocols for multifunctional heterogeneous nanostructures with high degree of controls in size, shape, surface and composition, we have further investigated the lattice mismatch for crystallographic parameters *c* and *a* for hexagonal phase $NaREF_4$ crystals.

Supplementary Table 2 shows the crystal lattice parameters of hexagonal phase $NaREF_4$ crystals. The difference between the two crystallographic parameters (*a* & *b*, and *c*) can affect the epitaxial growth of subsequent layers. For example, the $NaNdF_4$ crystals can be more easily grown over $NaGdF_4$ than over $NaYF_4$ due to the close similarities in their crystallographic parameters; likewise $NaGdF_4$ crystals prefer to grow over the $NaYF_4$ crystal lattice than over the $NaLuF_4$ crystal lattice.

By calculating the mismatch rate for the difference in the crystal lattice units, we can quantify the possibility of direct epitaxial growth of different crystal types onto the core crystal when forming a heterogeneous rare-earth doped single nanocrystals. **Supplementary Table 3** summarizes the lattice mismatch for crystallographic parameters *c* for hexagonal phase $NaREF_4$ crystals. The green area shown represents the pair crystals with a lattice mismatch that is less than 3%, which suggests a close crystal lattice match, while the yellow and the red areas mark a higher lattice mismatch rate of either 3.1% to 5% or 5.1% respectively, indicating greater difficulty in the direct epitaxial growth of a heterogeneous nanocrystal from these two constituents.

Supplementary Note 14 – Investigation on the migration growth mechanism

Our first example is to show the design and synthesis of $NaYF_4/NaGdF_4/NaNdF_4$ heterogeneous nanocrystals to form a heterogeneous nanoscale hourglasses. In this experiment, we observed for the first time a new epitaxial shell growth process and a migration growth pattern, which consists of the dissolution of $NaYF_4$ with the subsequent growth of the $NaNdF_4$ on the another facet. By real-time monitoring of the migration growth process, **Supplementary Fig. 26** provided direct evidence that migration growth consists of a two-step reaction, (1) the dissolution of $NaYF_4$ with subsequent (2) growth of $NaNdF_4$ on the another facet. The size of nanocrystal core decreased significantly in the first 5 minutes, but without forming new component crystal on the crystal surface, indicating that the speed of dissolution of the nanocrystals was faster than crystal growth speed in this reaction step; After 15 minutes, new crystals started to form onto the top and bottom ends of the core as the width of the nanocrystal cores decreased. This result directly rules out "surface mobility" ("atom diffusion") as the possible driving force, since if this was the case the decrease of $NaYF_4$ and the increase of NaNdF4 would happen at the same time, which was not the case with the sample taken after 5 minutes. The only mechanism that could explain the 5 minute sample was that the absence of the F^- ion source in the reaction mix at beginning makes $NaNdY_4$ growth impossible until the concentration of released F^- ions reaches a certain threshold.

Supplementary Fig. 26 shows the evolution process of the $NaYF_4/NaGdF_4$ cores as they form the $NaYF_4$-$NaGdF_4/NaNdF_4$ heterogeneous nanoscale hourglasses. In the modified protocol for longitudinal growth of heterogeneous nanocrystal rods, the absence of an F^- ion source with unbalanced F^- ion concentration causes the accelerated erosion of the $NaYF_4$ rods and the selective trimming off the $NaYF_4$ and the $NaGdF_4$ from the (100) facets on the side of the nanorods particles.

The elemental mapping images in **Supplementary Fig. 26 (h)** further reveals the distributions of the three ions Y^{3+}, Gd^{3+} and Nd^{3+} within one single nanocrystal, with the majority of the Y^{3+} ions in the middle, the Gd^{3+} ions as a bridge, and the minority of the Y^{3+} and Gd^{3+} ions migrating to the end section with the Nd^{3+} ions found only on the end of each nanocrystal.

Supplementary Fig. 27 (a) shows the appeared crystal lattice, which was confirmed as a $NaNdF_4$ (100) plane by the analysis which matched the lattice distance. From the side view, the lattice of (100) planes were also presented. The small difference of lattice distance at different areas was also measured, the lattice distance in the dark area was 5.25 Å that is close to the standard value for the $NaNdF_4$ (100) plane distance, while the lattice distance in the grey area was 5.09 Å which is close to the standard value of the $NaYF_4$ (100) plane distance. This result is consistent with the STEM data showed in the **Supplementary Fig. 26** .

Brief conclusion:

Oleate ions (OA^-) acting as surfactant ligands firmly bond to the rare earth ions on the side surfaces (100) facet planes; in the absence of an F^- source ion and at a high temperature (310 ^oC), the nanocrystal side surfaces become unstable and there is an observed accelerated site-selective erosion phenomenon. With the release of the F^- ions, the migration growth occurs when $NaYF_4$/$NaGdF_4$ nanocrystals as cores are fabricated in the condition for their longitudinal growth with $NaNdF_4$, but without a F^- ion source.

Supplementary Note 15 – Verify the driving force for the selective-surface etching

The observed dissolution (etching) phenomenon could possibly be caused by the relative crystal stability difference between $NaYF_4$ and $NaGdF_4$. To provide further insight into its formation, we designed the following experiment which selectively protected the side surface of $NaYF_4$ nanocrystal using a more stable $NaGdF_4$ nanocrystal shell. We provide experimental evidence that the surfactant oleate ions (OA^-) firmly bonds to the side-surface rare earth ions as the main factor which causes the loss of $NaYF_4$ and $NaGdF_4$ from the side surface of the particles.

By a transversal growth approach, $NaGdF_4$ shell was first grown on the side surfaces of $NaYF_4$, as shown in **Supplementary Fig. 29 (a)**. **Supplementary Fig. 29 (b)** to **(f)** recorded the evolution process of the epitaxial growth of the $NaNdF_4$ nanocrystals onto the $NaGdF_4$/$NaYF_4$ nano-prisms in the absence of the F^- ions and at a high temperature (310 ^oC). The a reduced diameter of $NaGdF_4$/$NaYF_4$ was observed by comparing **Supplementary Fig. 29 (a)** and **(b)** which clearly show that the dissolution (etching) only happens on the side surfaces for the first 10 minutes of the reaction. **Supplementary Fig. 29(c)** shows simultaneous etching from the side surfaces and epitaxial growth of the $NaNdF_4$ onto the end surfaces of the $NaGdF_4$/$NaYF_4$ cores, resulting in a thinner and longer "pupa-like" crystal. This indicates that the etching of $NaYF_4$ was relatively faster than the more stable $NaGdF_4$ nanocrystals, but it should be noted that the etching still mostly occurs on the side surfaces of nanocrystals with the OA^- ligands acting as a surfactant. Once the $NaGdF_4$ removed (as seen in **Supplementary Fig. 29 (d)**), the etching process appears more even on the side surface of $NaYF_4$ with a relatively smooth side surface created between the $NaNdF_4$ nanocrystal caps. Finally, the $NaYF_4$ crystals completely disappears after 60 minutes of reaction with only hexagonal-shape $NaNdF_4$ nanocrystals as the only yield.

Brief conclusion:

Although the side surface of $NaGdF_4$ was much more stable than the $NaYF_4$ and has a smaller mismatch than the $NaNdF_4$, etching still occurs on the side surface and the migration growth direction was still observed to occur from the side and the end surfaces. This suggests that in a highly unstable growth environment, such as a reaction at a high temperature, in absence of an F^- ion source, the strongly binding surfactant oleate ions (OA^-) remove the rare earth ions from the side surfaces of the particles which acts as the main factor driving behind the observed etching.

Supplementary Note 16 – Heterogeneous $NaYF_4/NaGdF_4/NaNdF_4$ NCs in flower shape

Our next example demonstrates the design and synthesis of $NaYF_4/NaGdF_4/NaNdF_4$ heterogeneous nanocrystals to form heterogeneous nanoscale flower-shaped particles. In this experiment, we demonstrate migration growth of the $NaNdF_4$ to the transversal structure rather than to the end surfaces. Using the principle that the direction of epitaxial shell growth can be controlled through adjustment of the ratio of OA^-/OAH, here we demonstrate that a low ratio of OA^-/OAH at relatively lower temperature can direct the migration growth instead along the transverse direction. The design logics of $NaYF_4/NaGdF_4/NaNdF_4$ nanocrystal in a flower shape are shown as below:

1. We first transversally grow $NaGdF_4$ nanocrystal shells onto the side surface of the $NaYF_4$ nanocrystal core;
2. In absence of the F^- ion source, the direct epitaxial shell growth of $NaNdF_4$ was stopped;
3. Considering the weak erosion process due to the low ratio of OA^-/OAH, we increased the reaction temperature from 290 ˚C to 300 ˚C to promote the erosion of $NaYF_4/NaGdF_4$ nanocrystals to release F^- ions. This has the effect of trimming off part of the $NaGdF_4$ shells around the $NaYF_4$ from its side surfaces;
4. A minimum amount of F^- ions slowly released from $NaYF_4/NaGdF_4$ nanocrystals into the reaction solution will help form the $NaNdF_4$ nanocrystals transversally from $NaGdF_4$ crystalline shell on side surfaces of the hexagonal prism;
5. Finally, F^- ions and part of Y^{3+} and Gd^{3+} ions migrate from the $NaYF_4$ to the $NaNdF_4$ section on the lateral faces of heterogeneous nanocrystals, forming a flower shape nanostructure.

By this transversal growth approach, $NaGdF_4$ shell was first grown on the side surfaces of $NaYF_4$, as shown in **Supplementary Fig. 30** (a). **Supplementary Fig. 30 (b) to (d)** record the evolution process of epitaxial growth of $NaNdF_4$ nanocrystals onto $NaGdF_4/NaYF_4$ nano-prisms in absence of F^- ions and at lower ratio of OA^-/OAH. The slightly reduced diameter of $NaGdF_4/NaYF_4$ is compared in **Supplementary Fig. 30 (a) and in (b)** some parts of $NaGdF_4$ was etched from the side surfaces after the first 10 minutes of reaction. **Supplementary Fig. 30 (c)** show the simultaneous etching of the side surfaces and the epitaxial growth of $NaNdF_4$ onto the $NaGdF_4$ area forming "island-shaped" anchors on the side surfaces of $NaGdF_4/NaYF_4$ cores. **Supplementary Fig. 30 (d)** shows that more $NaNdF_4$ was growth around the anchors which form a heterogeneous flower-shape nanocrystal.

Because there was few OA^- ligands on the side surface, OA^- mediated etching process for $NaGdF_4/NaYF_4$ nanocrystals dissolution of the side surfaces becomes very slow at this relatively lower reaction temperature. Despite high concentration of OAH on the end surfaces, no etching phenomenon was observed at the top and bottom end surface of $NaGdF_4/NaYF_4$ of the nano-prisms. This indirectly provides further evidence for the mechanism of OA^- mediated etching process from side surfaces.

Programmable longitudinal growth of heterogeneous nanocrystals was much easier and faster than programmable transversal growth in yielding mono-disperse morphology. More fine-tuning experiments, such as using lower temperature and much longer reaction time, were still required for the identification of the optimum reaction conditions for the fabrication of truly mono-disperse flower-shaped heterogeneous nanocrystals.

Brief conclusion:

The migration growth in transversal direction was made possible through the use of a low ratio of OA^-/OAH and a low reaction temperature, though the etching process was much less efficient at the side surfaces of nanocrystal due to fewer OA^- ligands bounding to rare earth ions to these side surfaces.

Supplementary Note 17 – Heterogeneous $NaYF_4/NaGdF_4/NaNdF_4$ NCs in dumbbell shapes

The design logics of $NaYF_4/NaGdF_4/NaNdF_4$ NCs in dumbbell shapes were described as below: the longitudinal growth direction was determined by adding a higher ratio of oleate (OA^-) to oleic acid (OA) ligands at a higher reaction temperature (310 ^oC), which selectively passivates the nanorods side surface (100) planes. A lower concentration of Nd^{3+} ion source will slow down the epitaxial growth on the ends and lead to a reduction in the diameter of the $NdYF_4$ nanocrystals. Moreover the absence of a F^- ion source will make the tips sharper while the presence of additional a F^- ion source leads to the formation of rounded-polished tips.

A decrease of a F^- and Nd^{3+} ion source in the reaction mix accelerates the etching of the $NaYF_4$. Since the $NaNdF_4$ is relatively more stable than $NaYF_4$, a released F^- ion source will form $NaNdF_4$ nanocrystals with $NaYF_4$ located in the rod middle area continuously becoming dissolved. This migration growth process of dissolving $NaYF_4$ and re-growing $NaNdF_4$ gradually diminishes as the Nd^{3+} ion source is consumed, which leads to the formation of sharp tips.

Supplementary Fig. 31 (c) and **Supplementary Fig. 32 (c)** show highly uniform heterogeneous nanocrystals. The STEM image (**Supplementary Fig. 31 (**d)) illustrates two separate partitions of tips and core particles. The elemental mapping images show the distributions of the Y^{3+}, Gd^{3+} and Nd^{3+} ions The combined elemental mapping image shows three well aligned components, consistent with the formation mechanism already described.

Brief conclusion:

The oleate ions (OA^-) mediated longitudinal growth, transversal growth, etching process and migration growth in consideration of crystal lattice mismatching rates and crystal stability have jointly formed a toolbox for highly controlled nanoscale materials engineering to fabricate rare-earth doped heterogeneous nanocrystals. The switches between these growth mechanisms are externally controllable by fine-tuning the reaction temperature, concentration and ratio of surfactant ligands, and elemental concentrations and balance in the reaction environment.

Supplementary Note 18 – $NaLuF_4$/$NaYF_4$ nanorods with $NaGdF_4$ double-ring structure.

Using the combinational approaches of longitudinal growth and transversal growth as well as taking the consideration of crystallographic mismatch rates, we demonstrate here a series of examples to illustrate our rational design and programmable epitaxial growth techniques for the bottom-up fabrication of 3-dimensional heterogeneous nanostructures.

The hexagonal-phase $NaYF_4$ nanocrystal is acknowledged elsewhere as the most efficient photon upconversion host [1]. Recent pioneer works has revealed that the incorporation of other functions in these nanocrystals can enrich their hybrid applications, for example, by providing X-ray computed tomography imaging through the use of $NaLuF_4$ as a host [1] or Magnetic Resonance Imaging using $NaGdF_4$ as a host [1]. Our first example here is to show the design and synthesis of $NaYF_4$/$NaLuF_4$/$NaGdF_4$ heterogeneous nanocrystals with two $NaGdF_4$ rings on a $NaLuF_4$ /$NaYF_4$ nanorod.

Design logics:

1. We first employed the approach of longitudinal growth to grow $NaYF_4$ nanoparticles to $NaYF_4$ NRs;
2. We then employed the protocol for transversally coating the side surfaces of $NaYF_4$ NRs with a thin layer of $NaLuF_4$. This thin layer of $NaLuF_4$ functions as a mask;
3. The surface of $NaLuF_4$ /$NaYF_4$ NRs at the top edge areas are less stable and are easier to dissolved after the transversal epitaxial shell growth, which leaves the $NaLuF_4$ mask at the end edges also becoming removed so that $NaYF_4$ was exposed;
4. According to Supplementary Table 3, the mismatch rate between $NaGdF_4$ and $NaLuF_4$ was about 4.3% while the mismatch rate between $NaGdF_4$ and $NaYF_4$ was about 2%, which suggests that the $NaGdF_4$ will prefer to grow on $NaYF_4$ rather than on $NaLuF_4$;
5. To facilitate the etching process, in our modified protocol towards transversal growth of $NaLuF_4$ onto the side surfaces of $NaYF_4$ NRs, absence of F source causes a faster erosion occur at the top edge area;
6. Finally, by injecting the α-$NaGdF_4$ nanocrystals with F element source by slowing dissolving it into the solution, β-$NaGdF_4$ nanocrystal shells start to grow onto the exposed $NaYF_4$ areas, forming double rings of $NaGdF_4$ shells transversally grown around the top and bottom edges of $NaLuF_4$/$NaYF_4$ NRs.

The elemental mapping images in **Supplementary Fig. 33 (c)** further confirms that the three elements of Y^{3+} , Lu^{3+} and Gd^{3+} are separated in three partitions within one single nanocrystal, with Y^{3+} only in the inner core of the rod, Lu^{3+} only in the outer shell of the rod, and Gd^{3+} only in the two rings. The high resolution TEM images shown in **Supplementary Fig. 34**, provides further evidence of the direct growth of crystalline structures. The two rings can be seen to grown around the [001] crystallographic direction and the select area electron diffraction pattern has a close match to theβ-$NaYF_4$, β-$NaLuF_4$ and β-$NaGdF_4$.

After a thin layer of $NaLuF_4$ shell is transversally formed onto the $NaYF_4$ core nanorods at 290˚C, the F element source was removed so that $NaLuF_4$ layer can start to dissolve slowly, particularly at the sharp end areas of nanorods where the $NaYF_4$ is firstly exposed. With the drop-wise addition of α-$NaGdF_4$ (as the F source) into the reaction system, $NaGdF_4$ prefer to grow onto the (100) (010) facets of the exposed $NaYF_4$ cores with $NaLuF_4$ shell acting as a mask because of the inherent difference in crystalline mismatch rate between $NaGdF_4$ vs. $NaYF_4$ and $NaGdF_4$ vs. $NaLuF_4$.

Brief conclusion:

We report for the first time a bottom-up programmable controlled fabrication of 3-D shaped heterogeneous nanocrystals using the combinational approaches of an oleate ion (OA^-) assisted longitudinal growth, transversal growth and selective etching which includes controlling for and using the crystallographic mismatch rates.

Supplementary Methods

Materials

Reagents: $YCl_3.6H_2O$ (99.99%), $YbCl_3.6H_2O$ (99.99%), $GdCl_3.6H_2O$ (99.99%), $NdCl_3.6H_2O$ (99.99%), $TbCl_3.6H_2O$ (99.99%), $LuCl_3.6H_2O$ (99.99%), NH_4F (99%), $ErCl_3.6H_2O$ (99.99%), KOH (99%), NaOH (99%), 1-octadecene (ODE) (90%) and oleic acid (OA) (90%) were purchased from Sigma-Aldrich. Unless otherwise noted, all materials were used as starting materials and without further purification.

Method for characterization of rare earth ions on the nanocrystal surface

Rare earth doped fluoride nanocrystals were usually synthesized by reacting lanthanide precursors in organic media in the presence of capping ligands to increase their size and shape uniformity. These nanocrystals are often heavily aggregated in aqueous solutions owing to the hydrophobic nature of the capping ligands; surfactants are typically introduced during the reaction to keep the crystals colloidally stable as they grow in reaction solvent. To test the amount of rare earth ions on their surfaces, we first remove surface capping ligands including both Oleic Acid and Oleate ions from $NaYF_4$ nanocrystals with a well followed procedure which is based on acid treatment [5]. The as-prepared ligands-capped nanocrystals are first dispersed in a 2 mL HCl solution (0.1 M) and ultrasonicated for 30 min, followed by their centrifugation at 14,500 rpm for 10 min and their purification by adding an acidic ethanol solution (at a pH of 4; prepared by adding 0.1 M HCl aqueous solution to the absolute ethanol). The resulting products of ligand-free nanocrystals are further washed with ethanol and deionized water three times, before they are re-dispersed in deionized water.

Methods for investigating the reaction temperature effect

The purpose of this experiment was to assess the role of different reaction temperature on the directional epitaxial growth of shells with the core. We adopted the same synthesis method for longitudinal growth to form $NaYF_4$ nanorods, except we step-wise varied the reaction temperature at 290 ˚C, 300 ˚C, 310 ˚C, 320 ˚C.

Method for investigating the role of OA concentration

The purpose of this experiment was to assess the role of OA (that is the sum of OA^- and OAH) concentration on the directional epitaxial growth of shells surrounding the core. We adopted the same synthesis method for longitudinal growth of $NaYF_4$ nanorods, except we step-wise varied the OA concentrations from 1.70 mmol/mL, 1.42 mmol/mL, 1.14 mmol/mL, and to 0.85 mmol/mL.

To keep other reagent concentrations as same as that used in the previous experiments, the same total reaction solvent volume of the four groups of different amount of OA and ODE, were applied separately.

Method for investigating the role of F^- concentration

The purpose of this experiment was to assess the role of varied F^- concentration to the directional epitaxial growth of shells surrounding the core. We adopted the same synthesis method for longitudinal growth of $NaYF_4$ nanorods, except we step-wise increased the amount of NH_4F from 0.4 mmol to 0.6 mmol, and to 0.8 mmol, with the F^- concentration calculated as 36.4 μmol/mL to 54.5 μmol/mL, and to 72.8 μmol/mL.

***Method* for investigating the role of Na^+ concentration**

The purpose for this experiment was to assess the role of Na^+ concentration in the directional epitaxial growth of shells onto the core. We adopted the same synthesis method for longitudinal growth of $NaYF_4$ nanorods, except we decreased the amount of NaOH from 0.5 mmol to 0.3 mmol, with the Na^+ concentration calculated as 45.5 μmol/mL to 27.3 μmol/mL.

Method for ruling out OH^- as a key factor for longitudinal growth

In this work, to generate longitudinal epitaxial growth of nanocrystals, we adopted a simple approach: Increases in the ratio of OA^- to OAH by increasing the amount of NaOH lead to the conversion of more OAH into OA^- and to changes in the morphology of the crystals. In order to rule out the interference from OH^- (from NaOH) that may play a role in directional epitaxial growth and as well to further verify if it is the oleate ion (OA^-) that do indeed play a major role in passivating the side surfaces (100), we further designed and ran an experiment that used sodium oleate (NaOA) to replace NaOH as the sodium source. As a comparison, in one control experiment we grow the $NaYF_4$ nanocrystals with $NaYF_4$ shell using NaOH as sodium source, and in the other experiment, we used the same molar amount of Na-OA (sodium oleate) as a sodium source and as a replacement for the NaOH.

0.2 mmol YCl_3 in 1 ml methanol solution was magnetically mixed with OA (9.5mmol) and ODE (25mmol) in a 50 mL three-neck round-bottom flask. The mixture was degassed under an Ar flow and heated to 150 ˚C for 30 min to form a clear solution, and then cooled to room temperature. 5 mL of methanol solution containing NH_4F (0.8 mmol) and **sodium oleic acid Na-OA (0.5 mmol)** was added and stirred for 60 min. The solution was slowly heated to 110 ˚C and kept at 110 ˚C for 30 min to completely remove any methanol and any remaining water. Then, 0.2 mmol of $NaYF_4$ in cyclohexane was injected into the nanocrystals in cyclohexaneand the mixture was kept at 110 ˚C for another 10

minutes so that the cyclohexane could be evaporated. Then, the reaction mixture was quickly heated to 310 ˚C and aged before aging for 1 hour.

Method for investigating the stability of shell material and its effect on crystal dissolution

The purpose for this experiment was to assess the stability of shell material and its relation to crystal dissolution. We adopted the same synthesis method for longitudinal growth of $NaGdF_4/NaYF_4$ nano-dumbbells, except $TbCl_3$ or $YbCl_3$ were used as shell precursor materials instead of $GdCl_3$. According to literature[2], the size and dipole polarizability of the lanthanide ion dominated the stability of crystal formation, and the nanocrystals formed with larger lanthanide ions have more stability. Yb^{3+} was chosen because its atomic radii is smaller than Y^{3+}, and the radii of Tb^{3+} is between Y^{3+} and Gd^{3+}. This, in principle, will make the core $NaYF_4$ more stable than the $NaYbF_4$ shell, but not as stable as the $NaTbF_4$ or the $NaGdF_4$ shells. The stability is ranked from high to low as $NaGdF_4$> $NaTbF_4$> $NaYF_4$ > $NaYbF_4$.

Method for verifying the driving force for the selective-surface etching.

0.4 mmol of $NdCl_3$ in 2 mL methanol solution was magnetically mixed with OA (9.5 mmol) and ODE (25 mmol) in a 50 mL three-neck round-bottom flask. The mixture was degassed under Ar flow and heated to 150 ˚C for 30 minutes to form a clear solution, and then cooled to room temperature. 5 mL of methanol solution containing KOH (0.8 mmol) and NaOH (0.8 mmol) was added and stirred for 60 minutes. The solution was slowly heated to 110 ˚C and kept at 110 ˚C for 30 minutes to completely remove the methanol and someof the water. The reaction mix was then injected with 0.1 mmol of 50 nm x 60 nm $NaYF_4/NaGdF_4$ core/shell nano-prisms ($NaGdF_4$ growing on the lateral faces of $NaYF_4$ nanocrystal), suspended in cyclohexane solution, into the reaction solution. After holding at 110 ˚C for a further 10 minutes to evaporate all the cyclohexane, the reaction mixture was quickly heated to 310 ˚C. 500 uL samples of the reaction solution was collected each time with a syringe at 5 minutes, 15 minutes, 30 minutes, 40 minutes, 50 minutes, and 60 minutes after the reaction started.

Transmission Electron Microscopy Characterization

Standard transmission electron microscope (TEM) measurements were performed using a Philips CM10 TEM with Olympus Sis Megaview G2 Digital Camera. The samples were prepared for TEM analysis by placing a drop of a dilute suspension of nanocrystals onto formvar-coated copper grids (300 meshes) and were allowed to dry in a desiccator at room temperature before using.

High resolution transmission electron microscope (TEM) measurements were performed with an aberration-corrected analytical transmission electron microscopy (TEM, JEOL ARM-200F) operated at 80 kV. High-angle annular dark-field (HAADF) scanning transmission electron microscope (STEM)

images and their corresponding element mapping images were collected with the same TEM equipped with a Centurio SSD energy-dispersive X-ray spectroscopy (EDS) detector set at 77 K.

Supplementary References

1 Clark, S. J. *et al.* First principles methods using CASTEP. *Z Kristallogr* **220**, 567-570, (2005).

2 Wang, F. *et al.* Simultaneous phase and size control of upconversion nanocrystals through lanthanide doping. *Nature* **463**, 1061-1065, (2010).

3 Sui, Y. Q., Tao, K., Tian, Q. & Sun, K. Interaction Between Y^{3+} and Oleate Ions for the Cubic-to-Hexagonal Phase Transformation of NaYF4 Nanocrystais. *J Phys Chem C* **116**, 1732-1739, (2012).

4 Wang, F., Deng, R. R. & Liu, X. G. Preparation of core-shell NaGdF4 nanoparticles doped with luminescent lanthanide ions to be used as upconversion-based probes. *Nat Protoc* **9**, 1634-1644, (2014).

5 Wang, F. *et al.* Tuning upconversion through energy migration in core-shell nanoparticles. *Nat Mater* **10**, 968-973, (2011).

6 Chen, D. & Huang, P. Highly intense upconversion luminescence in Yb/Er:NaGdF4@NaYF4 core-shell nanocrystals with complete shell enclosure of the core. *Dalton transactions* **43**, 11299-11304, (2014).

7 Chen, G. Y., Agren, H., Ohulchanskyy, T. Y. & Prasad, P. N. Light upconverting core-shell nanostructures: nanophotonic control for emerging applications. *Chem Soc Rev* **44**, 1680-1713, (2015).

8 Johnson, N. J. J. & van Veggel, F. C. J. M. Sodium lanthanide fluoride core-shell nanocrystals: A general perspective on epitaxial shell growth. *Nano Res* **6**, 547-561, (2013).

9 Li, C., Quan, Z., Yang, J., Yang, P. & Lin, J. Highly uniform and monodisperse β-NaYF4: Ln3+ (Ln= Eu, Tb, Yb/Er, and Yb/Tm) hexagonal microprism crystals: hydrothermal synthesis and luminescent properties. *Inorganic chemistry* **46**, 6329-6337 (2007).

10 Zhang, F. *et al.* Uniform Nanostructured Arrays of Sodium Rare-Earth Fluorides for Highly Efficient Multicolor Upconversion Luminescence. *Angewandte Chemie International Edition* **46**, 7976-7979 (2007).
